# Total Synthesis and Structural Revision of Keenamide A

**DOI:** 10.1021/acs.jnatprod.5c01325

**Published:** 2026-01-05

**Authors:** Lukas Koch, Christoph Wiedemann, Christoph Parthier, Rüdiger W. Seidel, Milton T. Stubbs, Mike Schutkowski, Marat Meleshin

**Affiliations:** † Institute of Biochemistry and Biotechnology, Department of Enzymology, Charles Tanford Protein Center, 9176 Martin Luther University Halle-Wittenberg, Kurt-Mothes-Straße 3a, 06120 Halle, Germany; ‡ Institute of Organic Chemistry and Macromolecular Chemistry, 201001 Friedrich Schiller University Jena, Humboldtstraße 10, 07743 Jena, Germany; § Institute of Biochemistry and Biotechnology, Department of Physical Biotechnology, Charles Tanford Protein Center, 9176 Martin Luther University Halle-Wittenberg, Kurt-Mothes-Straße 3a, 06120 Halle, Germany; ∥ Institute of Pharmacy, 9176 Martin Luther University Halle-Wittenberg, Wolfgang-Langenbeck-Straße 4, 06120 Halle (Saale), Germany

## Abstract

Stereochemical assignments of thiazoline-containing cyanobactins, a family of ribosomally synthesized and post-translationally modified peptides, are often complicated due to the propensity of the exomethine and C-4 chiral centers of thiazolines to epimerize under basic and acidic conditions. In this work, we re-evaluate the proposed configuration of the leucine-thiazoline moiety of the cyanobactin-like cyclopeptide keenamide A. Using a fast and adaptable strategy for the synthesis of thiazoline-containing cyclopeptides, we synthesized four possible keenamide A stereoisomers, one of which was oxidized to mollamide C, the thiazole analogue of keenamide A. Comparison of the NMR spectra of synthetic keenamide A stereoisomers with that of natural keenamide A combined with X-ray crystallographic analysis indicates that the originally proposed (*R*)-configuration of the thiazoline ring of keenamide A must be revised to (*S*)-configuration. This work highlights the power of synthetic methods to inform the structure elucidation and structural revision of natural products, especially in cases where isolation and purification of natural material are not readily achieved.

## Graphical abstract

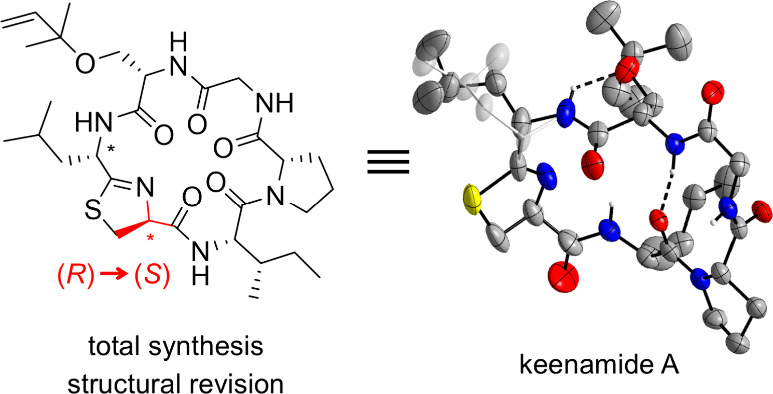

It is not uncommon for natural product structures to be revised in light of (re)-analysis of spectroscopic data, crystallographic studies, or total synthesis.^1–4^ Although revisions are common, most structures, once published, are never revisited, leaving errors unnoticed and uncorrected. This challenge is aggravated by the fact that natural products often appear as families of structurally similar compounds. When new structures are assigned based on spectral similarity to previously reported but incorrect compounds, initial misassignments can cast a long shadow, perpetuating further errors.^4^ The correctness of published structures is of critical importance not only for basic science but also for translational research, as a substantial proportion of newly approved drugs is based on lead structures derived from natural products,^5^ and structures of compounds with promising bioactivities guide medicinal chemists in their search for novel drugs.

Developments in crystallographic methods such as microfocus crystallography,^6^ the use of crystalline sponges,^7^ and microcrystal electron diffraction (MicroED)^8^ have reduced the requirement for large, high-quality single crystals for X-ray crystallography, once a bottleneck for crystallographic structure determination. These methods, however, are applicable only when authentic material of the compound of interest is available. Unfortunately, this condition is often unmet for natural products, which are, in many cases, isolated only in minute amounts or from rare sources.

Computational methods such as the prediction or quantum-chemical calculation of spectroscopic or chiroptical properties of a compound offer complementary approaches to structure elucidation.^9–11^ Yet these approaches can become prohibitively complex for large molecules, although some progress has been made in this direction.^12^

Perhaps the most common are structural revisions after total synthesis.^1^ Although often complicated and laborious to achieve, chemical synthesis of a proposed structure not only furnishes structure elucidation with new information but also enables synthetic access to the compound, facilitating further biological investigation.

Cyanobactins are a structurally diverse family of ribosomally synthesized and post-translationally modified peptides (RiPPs).^13,14^ Widespread among cyanobacteria, cyanobactins were originally isolated from sea slugs and marine ascidians such as *Didemnum molle* or *Lissoclinum patella*, which harbor cyanobacterial symbionts of the genus *Prochloron*.^15^ Most known cyanobactins are small cyclopeptides that usually contain at least one proline residue and one or more thiazoline, oxazoline, or methyloxazoline heterocycles derived from the cyclization of cysteine, serine, or threonine residues, respectively (Chart 1). In many cyanobactins, these heterocycles are further oxidized to their azole counterparts; for instance, lissoclinamide 5 is a thiazole analog of lissoclinamide 4. If not cyclized, serine and threonine residues may be modified by reverse prenylation, as is the case in trunkamide.^16^

**Chart 1:**
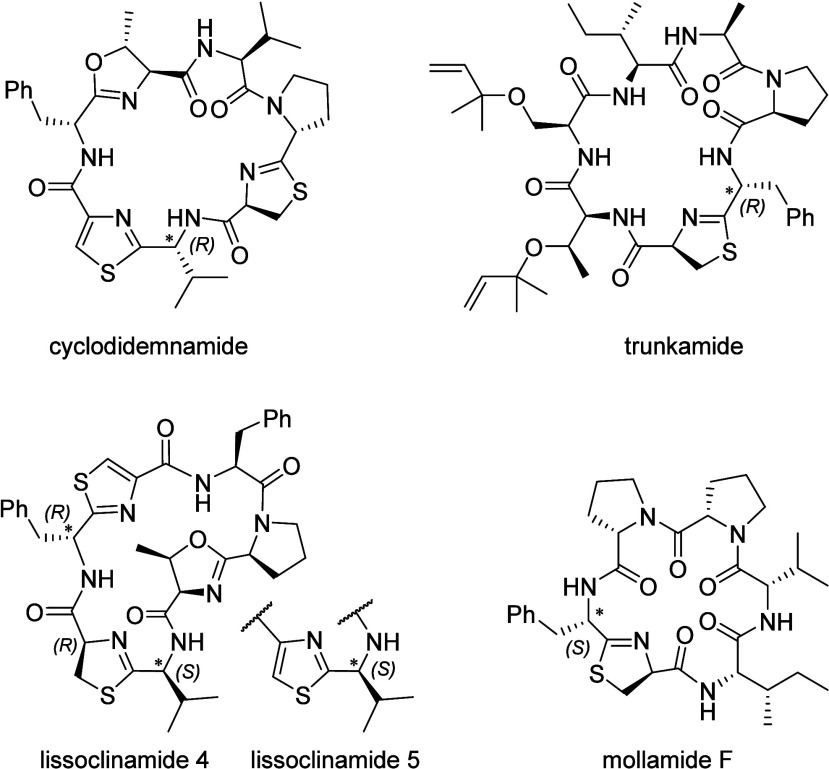
Structures of Selected Cyanobactins with Configuration as Revised after Total Synthesis^a^

a Revised stereocenters are marked with an asterisk.

The presence of sulfur-containing thiazole and thiazoline heterocycles is the main reason that the cyanobactin family has a rich history of structural revisions (Chart 1). First, the stereocenter in the C-4 position of a 4-substituted 2-thiazoline ring is prone to epimerization under basic conditions.^17^ Second, the exomethine stereocenters adjacent to a thiazole or thiazoline ring, i.e., at the C^α^ of the preceding amino acid, are notoriously labile and prone to epimerization under mildly basic conditions^18^ and, more importantly, under the acidic conditions of total hydrolysis.^18–21^ Since acid hydrolysis is usually the first step in chiral amino acid analyses, this brings a significant risk of misassignments. In some cases, acid hydrolysis of thiazole- or thiazoline-containing cyclopeptides unexpectedly yielded the unnatural epimer in excess. For example, the valine residue of lissoclinamide 4 was originally assigned the d-configuration based on a d/l ratio of 1.5 in the hydrolysate.^20,22^ The total synthesis of the proposed structure revealed a spectral mismatch between the synthetic and natural material, ultimately leading to revision of the structures of lissoclinamides 4 and 5 to contain l-Val.^23^ Similarly, the structures of cyclodidemnamide^24,25^ and trunkamide^16,26^ were revised after the synthesis of their proposed structures.

Recently, we reported a rapid synthesis of mollamide F via cyclodesulfhydration of a thioxopeptide precursor, which led to the revision of its configuration.^27^ In this case, the configuration of the thiazoline-exomethine stereocenter had been incorrectly assigned, and the thiazoline-adjacent phenylalanine residue was revised to l-Phe. We demonstrated that both epimers yield the same proportion of d/l-Phe after total hydrolysis, with d-Phe formed in excess. This observation may be explained mechanistically by faster hydrolysis of the unnatural d-Phe epimer, which under acidic conditions is expected to exist in equilibrium with the natural l-Phe epimer, thus altering the d/l ratio in favor of the d-epimer. A similar behavior likely accounts for many structural misassignments of related thiazoline-containing peptides.

The cytotoxic cyclopeptide keenamide A, isolated originally from the marine mollusk *Pleurobranchus forskalii*, displays characteristic structural features of the cyanobactin family, including head-to-tail cyclization, a thiazoline ring, and a reverse prenylated serine residue (Ser­(rPr)).^28^ In a later work, keenamide A was isolated from the ascidian *Didemnum molle* along with its thiazole analog mollamide C (Chart 2).^29^

**Chart 2:**
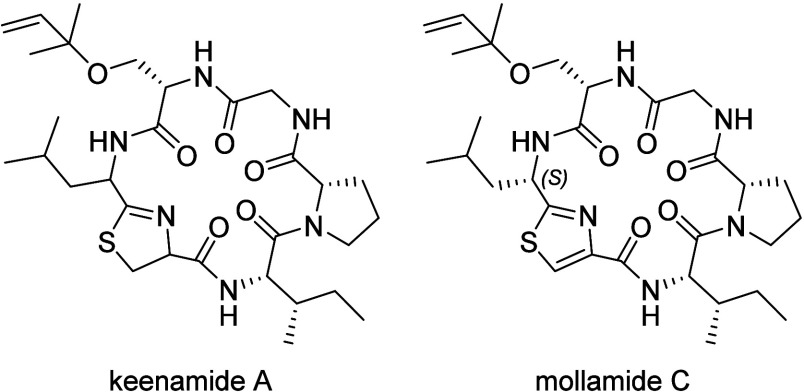
Structures of Keenamide A and Mollamide C

Our experiences with mollamide F led us to question the proposed configuration of two stereocenters in keenamide A. First, the thiazoline exomethine stereocenter, i.e., C^α^ of the leucine residue, was assigned as l/(*S*)-configuration based on an excess of l-Leu over d-Leu detected after acid hydrolysis. Second, the l/(*R*)-configuration was assigned to the stereocenter inside the thiazoline ring, i.e., C^α^ of the cysteine residue from which the thiazoline ring is formed, on the basis that l-Ser, but not d-Ser, was detected after hydrolysis and derivatization, assuming that the thiazoline moiety was degraded to serine during hydrolysis. It is, however, well established that cyanobactin thiazoline rings are typically hydrolyzed to cysteine.^16,20,29,30^ Thus, it is more plausible that l-Ser was solely derived from l-Ser­(rPr) and that cysteine was simply not recovered after hydrolysis and derivatization, leaving the configuration of the thiazoline ring in keenamide A uncertain. Finally, most cyanobactins contain at least one d-amino acid, and the thiazoline-isoleucine motif of keenamide A resembles the d-thiazoline-l-isoleucine motif of mollamide F. We therefore hypothesized that the proposed structure of keenamide A is likely incorrect and warrants revision.

## Results and Discussion

For the synthesis of the proposed structure of keenamide A (**1**), we used a combined solid- and liquid-phase strategy, with thioacyl-cysteine desulfhydration leading to the formation of thiazoline as a key step—a method that proved efficient in our recent total synthesis of mollamide F (Scheme 1).^27^ This approach required two nonstandard Fmoc-protected building blocks: the thioxylated leucine nitrobenzotriazolide Fmoc-d-Leu^S^-NBt, synthesized in three steps from Fmoc-d-Leu-OH using established methods,^31^ and the reverse prenylated Fmoc-Ser­(rPr)-OH.

**Scheme 1:**
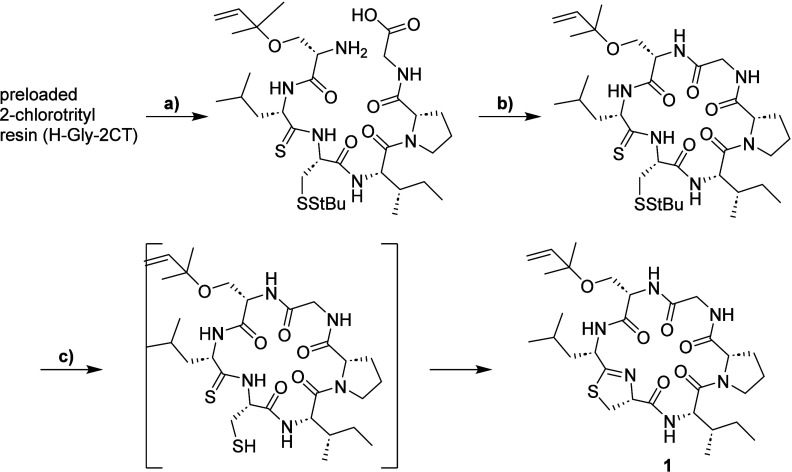
Synthesis of the Proposed Structure of Keenamide A (**1**)­^a^

a (a) (1) Automated Fmoc solid-phase peptide synthesis; (2) HFIP/DCM (1:4 v/v); (b) (1) EDC·HCl, HOBt, DMF/DCM (1:9 v/v); (2) 2-mercaptoethanol; (c) TCEP, K_2_CO_3_, H_2_O/DMF, pH 8–8.5.

For the synthesis of Fmoc-Ser­(rPr)-OH, we employed a strategy based on the nucleophilic ring opening of the *N*-protected 2-aziridinecarboxylic acid ester Trt-Azy-OMe with an alcohol in the presence of BF_3_OEt_2_.^32^ Starting from *N*-trityl serine, we first prepared the *N*-Trt-protected aziridine intermediate, followed by the exchange of the Trt group for an Fmoc group. However, in our hands, the aziridine ring opening of Fmoc-Azy-OMe with 2-methyl-3-buten-2-ol catalyzed by BF_3_OEt_2_ proceeded very slowly and yielded only trace amounts of the desired product Fmoc-Ser­(rPr)-OMe. The major product formed was Fmoc-Ser-OMe, which we attributed to partial dehydration of 2-methyl-3-buten-2-ol and hydrolysis of the starting material by the released water. After testing several variations of the reaction conditions, we found that the addition of activated molecular sieves increased the yield of the product Fmoc-Ser­(rPr)-OMe to 60%.

To hydrolyze the methyl ester in the presence of the base-labile Fmoc group, we tested both the LiOH and NaOH/CaCl_2_ methods.^33^ As partial Fmoc cleavage was observed, we added 2-mercaptoethanol as a dibenzofulvene scavenger during hydrolysis and subsequently reintroduced the partially cleaved Fmoc group using Fmoc-OSu, which increased the yield of Fmoc-Ser­(rPr)-OH to 80% (Scheme 2).

**Scheme 2:**
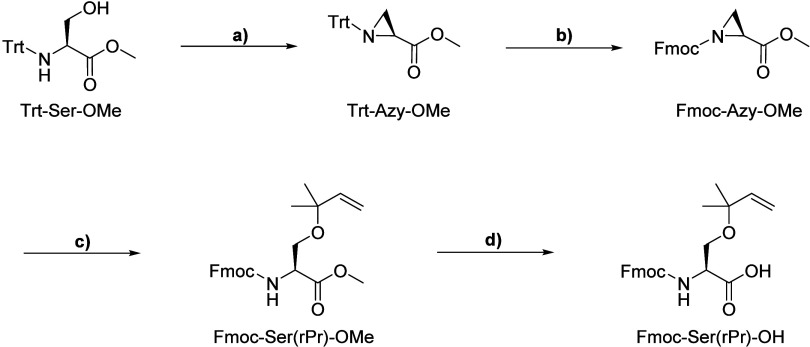
Synthesis of the Reverse Prenylated Serine Building Block Fmoc-Ser­(rPr)-OH^a^

a (a) MsCl, TEA, THF, 100%; (b) (1) TFA, DCM; (2) 2,4,6-collidine, Fmoc-Cl, 96%; (c) 2-methyl-3-buten-2-ol, BF_3_OEt_2_, molecular sieves 4 Å, DCM, 60%; (d) (1) LiOH, 2-mercaptoethanol, THF/H_2_O; (2) pH adjusted to 8–8.5 with citric acid and then Fmoc-OSu, 80%.

The linear precursor was synthesized on a 2-chlorotrityl resin preloaded with glycine using an Fmoc-based automated solid-phase peptide synthesis. Following cleavage from solid support with hexafluoroisopropanol (HFIP)/DCM, the next three transformations (macrocyclization, reduction of the disulfide bond with tris­(carboxyethyl)­phosphine (TCEP), and thiazoline formation under mildly basic conditions) were carried out *in situ* (Scheme 1). After preparative HPLC purification, compound **1** was obtained in 29% overall yield based on resin loading.

The synthesized compound exhibited an absorption maximum at 256 nm, characteristic of thiazolines. ESI-MS, as well as the ^1^H and ^13^C NMR spectra, were in good agreement with the structure of compound **1**. However, comparison of the NMR spectra of **1** with those of natural keenamide A revealed clear discrepancies, particularly in the NH region, indicating that **1** and keenamide A are not the same (Figures 1 and S43). Treatment of **1** with 1,8-diazabicyclo[5.4.0]­undec-7-ene (DBU) afforded clean conversion into an isomeric compound, the ^1^H NMR spectrum of which closely resembled that published for keenamide A, suggesting that keenamide A is a thermodynamically favored stereoisomer of **1**.

**Figure 1:**
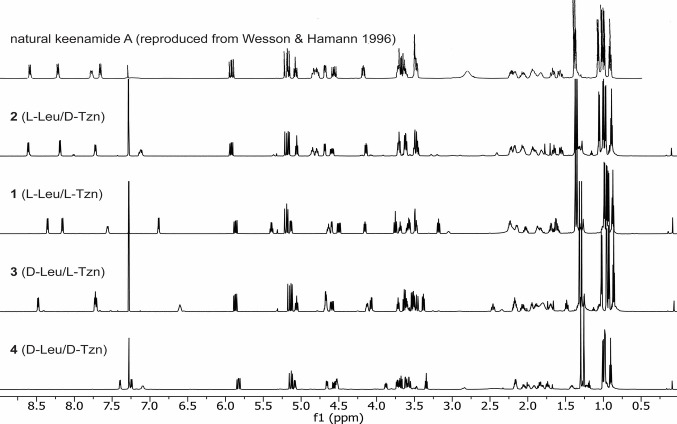
^1^H NMR spectra of natural keenamide A and synthetic peptides **1–** **4** (CDCl_3_, 500 MHz). The spectrum of natural keenamide A was reproduced from that of K. J. Wesson, M. T. Hamann, and Keenamide A, a Bioactive Cyclic Peptide from the Marine Mollusk Pleurobranchus forskalii. *J. Nat. Prod.* **59**, 629–631 (1996), DOI: 10.1021/np960153t. Copyright 1996 American Chemical Society.^28^ The baseline of this spectrum was digitally enhanced to ensure legibility.

To determine the correct structure of natural keenamide A, we synthesized the three additional stereoisomers with opposite configurations at one or both of the two most labile stereocenters within the Leu-Tzn fragment (Chart 3). Using the same reaction sequence described for compound **1**, compounds **2**, **3**, and **4** were prepared in overall yields of 29, 26, and 45%, respectively. Notably, the high efficiency and reliability of this synthetic approach highlight its potential utility for the synthesis of related cyclic thiazoline-containing peptides. The identities of the synthesized compounds were confirmed with UV–vis spectroscopy, HPLC-MS, and ^1^H and ^13^C NMR analysis.

**Chart 3:**
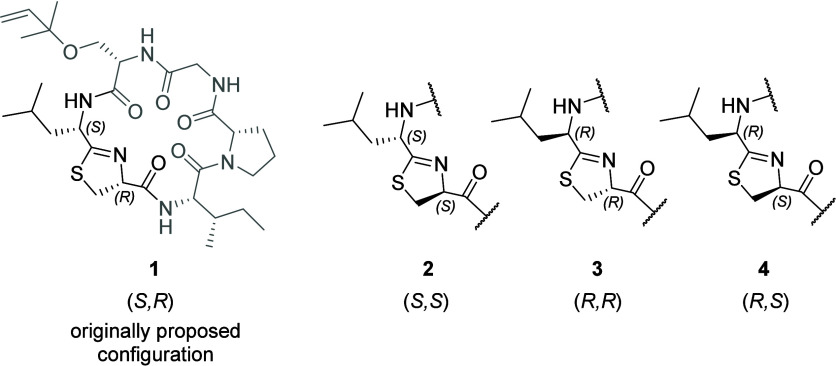
Keenamide A Stereoisomers **1–** **4** with Opposite Configuration at One or Both Stereocenters of the Leu-Thz Fragment

Although all synthesized cyclic thiazoline-containing peptides **1–4** exhibited very similar UV–vis and mass spectra, their NMR spectra and chromatographic behavior in RP-HPLC differed significantly (Figures S9–S35 and Tables S1–S4). Comparison of the NMR spectra demonstrated that the ^1^H NMR spectrum of **2** closely matches that reported for natural keenamide A (Figures 1 and S43), only differing in the chemical shift of the glycine amide proton. No authentic material was available to us for direct comparison. Interestingly, we found variable chemical shifts for the glycine amide proton in different batches of **2**, suggesting that the chemical shift of this proton is highly sensitive to the experimental conditions. We therefore conclude that despite this spectral mismatch, **2** is identical to keenamide A and that the latter must be revised to feature an (*S*)- rather than (*R*)-configuration of the C-4 stereogenic center of the thiazoline ring.

In our recent work, we described that mollamide F was significantly more stable toward acidic hydrolysis than its l-phenylalanine epimer.^27^ Thus, we compared the stability of keenamide A isomers **1–** **4** under acidic conditions and found keenamide A (**2**) to be surprisingly stable at pH 2 and mostly intact even after 24 h, whereas **1**, **3**, and **4** were completely degraded within several hours (Figure S44).

Having synthetic keenamide A in hand, we attempted the synthesis of mollamide C by direct oxidation of the thiazoline ring of **1** to a thiazole ring.^34^ Stirring the solution of **1** with bromotrichloromethane and DBU in DMF at room temperature achieved a smooth conversion to mollamide C (Scheme 3). The yield after purification with preparative HPLC was 40%. The structure of synthesized mollamide C was confirmed by ESI-MS, UV/vis, and NMR spectroscopy.

**Scheme 3:**
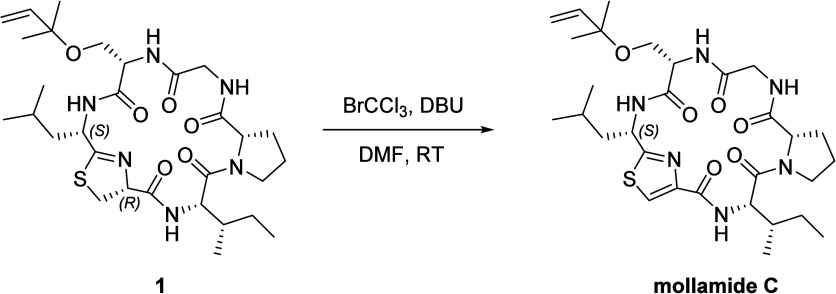
Oxidation of **1** to Mollamide C

Both keenamide A and mollamide C were found to crystallize upon the removal of MeCN from HPLC fractions using a rotary evaporator. This observation prompted us to attempt crystallization of the compounds from MeCN/water mixtures in a hanging-drop setup, allowing structural elucidation using X-ray crystallography. Both compounds adopt similar structures in the crystal, with the hydrophobic amino acid side chains on the convex face of the macrocycle (Figure 2). Most amide hydrogen atoms are buried in the structure or involved in hydrogen bonding to the backbone carbonyl and Ser­(rPr) ether oxygen atoms, except for the glycine amide protons, which are solvent-exposed and hydrogen-bonded to water molecules in the crystal. The latter observation likely explains why we observed variable chemical shifts of the glycine amide proton in the ^1^H NMR spectra. The extensive hydrogen bonding network probably accounts for the observed retention time differences of **1**, **2**, **3**, and **4** in RP-HPLC: an altered configuration of one of the amino acids may result in a conformational change of the macrocycle, presumably leaving more amide hydrogen atoms exposed, which would result in more hydrophilic behavior.

**Figure 2:**
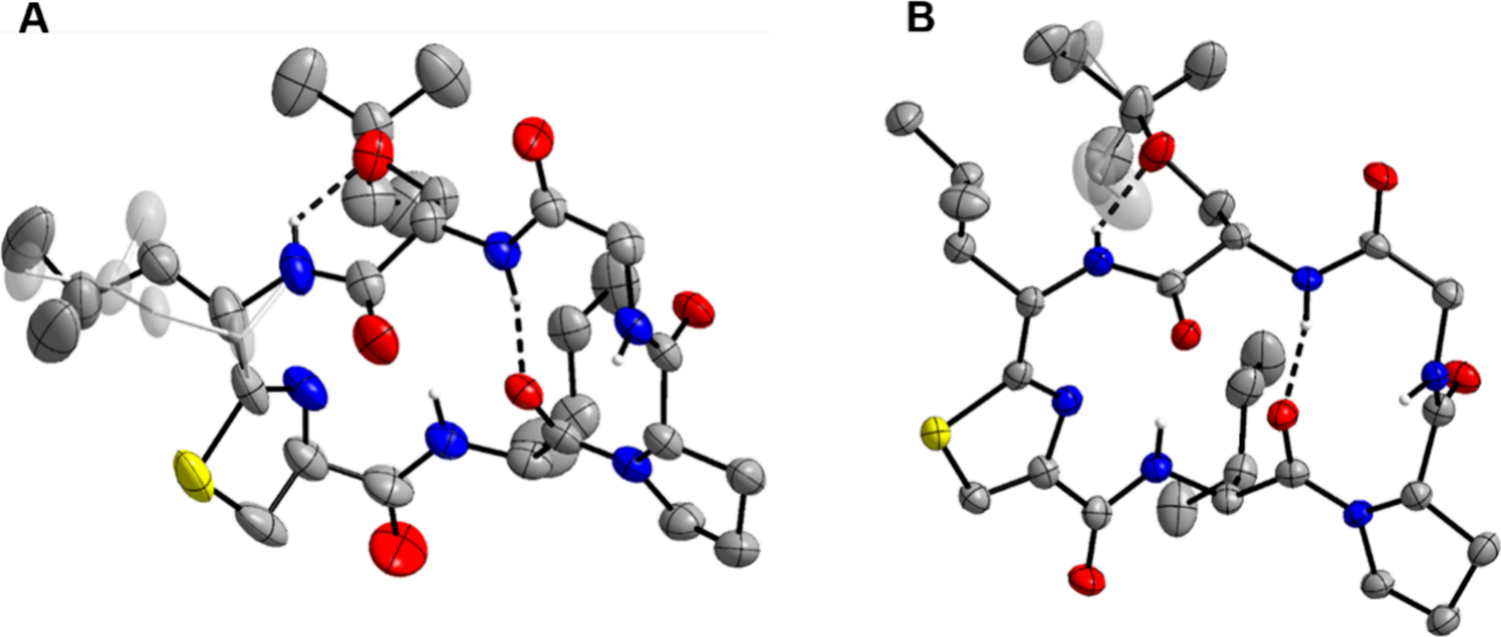
Molecular structures of keenamide A (A) and mollamide C (B) determined by X-ray crystallography. Displacement ellipsoids are drawn at the 50% probability level. Carbon-bound H atoms and solvent water molecules have been omitted for clarity. The minor conformers of the disordered Leu-Tzn iso-butyl group of keenamide A and the prenyl group of mollamide C (ca. 41 and 42% occupancy, respectively) are shaded. Dashed lines represent intramolecular N–H···O hydrogen bonds. Color scheme: C, gray; H, white; N, blue; O, red; S, yellow.

## Experimental Section

### General Experimental Procedures

Fmoc-protected amino acids were purchased from GL Biochem Ltd. (Shanghai, China). DMF, HFIP, and Oxyma were purchased from Iris Biotech (Marktredwitz, Germany). DIPEA, TFA, and DCM were purchased from Carl Roth (Karlsruhe, Germany). Unless stated otherwise, the reagents and solvents were used without further purification. Fmoc-l-Leu^S^-NBt and Fmoc-d-Leu^S^-NBt were prepared as described elsewhere.^31^ All other chemicals were purchased from Sigma-Aldrich/Merck KGaA (Darmstadt, Germany). Toluene, DCM, THF, DMF, and 2-methyl-3-buten-2-ol were stored over 4 Å molecular sieves. TEA was stored over KOH. THF was filtered through activated alumina before use. CDCl_3_ was stored over Ag and filtered through silica gel prior to NMR spectroscopy sample preparation. NMR spectra were recorded on Agilent Technologies DDS (500 MHz) or Bruker Avance III (700 MHz) spectrometers, and chemical shifts are reported in ppm with reference to TMS, whereby residual solvent signals were used as internal standards (CDCl_3_: ^13^C, 77.16 ppm; ^1^H, 7.26 ppm; DMSO-*d*_6_: ^13^C, 39.52 ppm; ^1^H, 2.50 ppm). High-resolution mass spectra were recorded on a Q Exactive Orbitrap mass spectrometer (Thermo Fisher Scientific) as described earlier.^27^ For TLC, silica-coated aluminum plates (silica gel 60, Merck KGaA, Darmstadt, Germany) with fluorescent indicator were used, and compounds were detected under UV light (254 nm). Dry-column vacuum chromatography (DCVC) was performed as described elsewhere using step gradients of heptane/EtOAc mixtures.^35^

### Synthesis of Keenamide A Stereoisomers 1–4

Keenamide A stereoisomers **1–** **4** were synthesized on a 0.1 mmol scale in a Liberty Blue peptide synthesizer (CEM, Kamp-Lintfort, Germany) using the Fmoc strategy starting from an Fmoc-glycine preloaded 2-chlorotrityl resin (100–200 mesh, loading: 1.6 mmol of Cl^–^/g, Iris Biotech GmbH, Marktredwitz, Germany). Fmoc deprotection was performed using 10% piperazine in NMP/EtOH (9:1 v/v) + 0.1 M HOBt for 5 min and then 10 min. Conventional Fmoc amino acids were coupled using a cocktail of Oxyma/DIPEA solution (0.5 mL, 1 M/0.4 M in DMF, 5 equiv Oxyma), DIC (0.5 mL, 1 M in DMF, 5 equiv), and Fmoc amino acid solution (2.5 mL, 0.2 M in DMF, 5 equiv) for 60 min. For the coupling of Fmoc-Ser­(rPr)-OH, the concentration of Fmoc-Ser­(rPr)-OH was reduced to 0.12 M, and the reaction time was extended to 180 min. Fmoc-protected thioxylated leucine residues were introduced by addition of Fmoc-Leu^S^-NBt (**1**,**2**-l-Leu; **3**,**4**-d-Leu; 4 equiv, 0.1 M solution in CHCl_3_) to the resin, followed by the addition of 2,4,6-collidine (3% v/v in DMF, 1 mL) after 2 min with a total incubation time of 47 min. Washing steps were performed with DMF (2 × 2 and 1 × 3 mL). Prior to cleavage of the peptides from the solid support, resins were washed thoroughly with DCM (5 mL) and dried in a stream of nitrogen. Cleavages were performed by treatment of the resins with HFIP/DCM (1:4 v/v) for 2 × 10 min, yielding the linear precursor peptides as off-white solids. Cleavage solutions were evaporated to dryness, and the cleaved precursor peptides were used in the next step without purification.

For macrocyclization, the crude linear precursor peptides (approximately 0.1 mmol) were dissolved in a solution of DMF (3 mL) and DCM (27 mL), and anhydrous HOBt (2 equiv) and EDC·HCl (2 equiv) were added to the stirring solution. Reactions were monitored using analytical HPLC (Phenomenex 5 μm XB-C18 (250 × 4.6 mm, 100 Å) column, 30–95% MeCN/H_2_O + 0.1% TFA, 0.8 mL/min).

After complete conversion of the starting material (10–15 min), DCM was removed under reduced pressure. To quench excess EDC, 2-mercaptoethanol (14 μL, 0.2 mmol, 2 equiv) was added to the residual DMF solution, and stirring was continued for 5 min.

Reductive cleavage of the StBu protection group was then started by addition of buffered TCEP solution (1.0 mL, 500 mM, pH 7.0) as supplied by Sigma-Aldrich/Merck KGaA (Darmstadt, Germany), and the pH was adjusted to 8–9 using K_2_CO_3_ solution (10% in H_2_O, approximately 600 μL). Stirring was continued under argon until complete consumption of the starting material as judged by analytical HPLC. The mixture was evaporated to dryness, and the residue was redissolved in MeCN/H_2_O for purification using preparative HPLC (Lichrospher 100 RP-8 column, 5 μm, 250 × 25 mm; eluent A: 10 mM Et_3_NH^+^Ac^–^/H_2_O, eluent B: 10 mM Et_3_NH^+^Ac^–^/MeCN/H_2_O (9:1); 40–90% B in 45 min). Repeated lyophilization of pure fractions afforded keenamide A stereoisomers **1**-**4** as white solids. l-Leu/l-Tzn (**1**) 18.3 mg (29%), ESI-HRMS: *m*/*z* 621.3428 [M + H]^+^; l-Leu/d-Tzn (**2**) 18.0 mg (29%), *m*/*z* 621.3428 [M + H]^+^; d-Leu/l-Tzn (**3**) 16.0 mg (26%) *m*/*z* 621.3430 [M + H]^+^; d-Leu/d-Tzn (**4**) 22.6 mg (44.5%), *m*/*z* 621.3430 [M + H]^+^ (calcd for C_30_H_49_N_6_O_6_S^+^, 621.3429).

### Mollamide C

(1) **1** (3.0 mg, 4.8 μmol) was dissolved in dry DMF (150 μL) under an inert gas atmosphere. DBU (2.4 μL, 9.6 μmol) and CBrCl_3_ (1.6 μL, 9.6 μmol) were added. After 2h, the mixture was diluted with H_2_O and subjected to HPLC (Uptisphere strategy C18-HQ column, 5 μm, 250 × 10 mm, eluent A: 0.05% TFA/H_2_O, eluent B: 0.05% TFA/MeCN; 40–70% B in 45 min). Lyophilization of pure fractions yielded mollamide C as a white solid (1.2 mg, 1.9 μmol, 40%).

(2) **1** (15 mg, 0.024 mmol) was dissolved in dry DMF (500 μL) under an inert atmosphere. DBU (7.2 μL, 0.048 mmol) and CBrCl_3_ (4.8 μL, 0.048 mmol) were added, and the mixture was stirred for 4 h. Additional DBU (7.2 μL, 0.048 mmol) and CBrCl_3_ (4.8 μL, 0.048 mmol) were added, and stirring was continued for 20 h. The mixture was diluted with H_2_O and lyophilized. The residue was dissolved in MeCN/H_2_O and purified by preparative HPLC (Hibar RP-8 prepacked column, 5 μm, 250 × 25 mm; eluent A: 0.05% TFA/H_2_O, eluent B: 0.05% TFA/MeCN; 40–70% B in 45 min). After lyophilization of pure fractions, 4.6 mg of mollamide C was obtained as a white solid (7.4 μmol, 31%).

ESI-HRMS: *m*/*z* 619.3267 [M + H]^+^ (calcd for C_30_H_47_N_6_O_6_S^+^, 619.3272).

### Amino Acid Building Blocks

#### Trt-Azy-OMe (Methyl (*S*)-1-tritylaziridine-2-carboxylate)

Methyl trityl-l-serinate (3.14 g, 9.15 mmol) was dissolved in dry THF (30 mL), and TEA (2415 μL, 20.1 mmol, 2.2 equiv) was added. To the stirring solution, mesyl chloride (960 mg, 9.24 mmol, 1.01 equiv) was added dropwise under an inert gas atmosphere. After 30 min, the solution was heated to 66 °C in a screw-capped vial, and stirring was continued for 3 days. Solvents were removed in vacuo, and the residue was dissolved in EtOAc (100 mL). The solution was washed with citric acid solution (10%, 3 × 15 mL) and saturated sodium bicarbonate solution (3 × 15 mL) with back-extraction. The combined organic layers were dried over anhydrous MgSO_4_, filtered, and concentrated to dryness to leave the product as an off-white solid (2.99 g, 100%).

ESI-HRMS: *m*/*z* 344.1636 [M + H]^+^ (calcd for C_23_H_22_NO_2_, 344.1645, low abundance), *m*/*z* 366.1460 [M + Na]^+^ (calcd for C_23_H_21_NaNO_2_, 366.1470, low abundance), *m*/*z* 243.1166 [Ph_3_C]^+^ (calcd for C_19_H_15_, 243.1168); *R*_f_ = 0.66 (EtOAc/petroleum ether, 1:1 v/v); ^1^H NMR (500 MHz, DMSO-*d*_6_) δ 7.40 (dd, *J* = 8.5, 1.4 Hz, 6H), 7.33 (t, *J* = 7.7 Hz, 6H), 7.26 (m, 3H), 3.69 (s, 3H), 2.23 (dd, *J* = 2.8, 1.4 Hz), 1.70 (dd, *J* = 6.2, 2.8 Hz), 1.31 (dd, *J* = 6.2, 1.4 Hz); ^13^C NMR (126 MHz, DMSO-*d*_6_) δ 170.9, 143.3, 128.8, 127.8, 127.1, 73.8, 52.0, 28.1.

#### Fmoc-Azy-OMe (1-(9*H*-Fluoren-9-ylmethyl)-2-methyl (*S*)-aziridine-1,2-dicarboxylate)

Trt-Azy-OMe (500 mg, 1.46 mmol) was dissolved in DCM (15 mL) and MeOH (60 μL, 1.45 mmol). TFA (0.25 mL, 3.22 mmol) was added at 0 °C under a protective atmosphere of argon. After 30 min, collidine (1060 μL, 8 mmol) was added to the mixture at −21 °C. A solution of Fmoc-Cl (396 mg, 1.53 mmol, 1.05 equiv) in DCM (2 mL) was added in one portion. The ice bath was removed after 15 min. After 30 min, a citric acid solution (10%, 10 mL) was added. The organic layer was washed with H_2_O (10 mL) and brine (10 mL), dried over anhydrous MgSO_4,_ and concentrated. The residue was purified by DCVC to give Fmoc-Azy-OMe as a colorless oil (450 mg, 96%).

ESI-HRMS: 324.1227 [M + H]^+^ (calcd for C_19_H_18_NO_4_, 324.1230); *R*_f_ = 0.52 (EtOAc/petroleum ether, 1:1 v/v); ^1^H NMR (500 MHz, DMSO-*d*_6_) δ 7.91 (d, *J* = 7.7 Hz, 2H), 7.68–7.61 (m, 2H), 7.43 (td, *J* = 7.6, 1.0 Hz, 2H), 7.35 (tdd, *J* = 7.5, 3.6, 2.2 Hz, 2H), 4.48 (dd, *J* = 10.3, 6.4 Hz, 1H), 4.33 (dd, *J* = 10.3, 6.8 Hz, 1H), 4.28 (t, *J* = 6.6 Hz, 1H), 3.65 (s, 3H), 3.20 (td, *J* = 5.6, 3.2 Hz, 1H), 3.17 (d, *J* = 5.2 Hz, 1H), 2.43 (dd, *J* = 3.2, 1.5 Hz, 1H); ^13^C NMR (126 MHz, DMSO) δ 168.5, 160.3, 143.4, 140.8, 127.8, 127.2, 125.0, 120.2, 67.6, 52.6, 48.6, 34.3, 31.0.

#### Fmoc-Ser­(rPr)-OMe (Methyl-(2*S*)-2-(((9*H*-fluoren-9-ylmethoxy)­carbonyl)­amino)-3-((2-methylbut-3-en-2-yl)­oxy)­propanoate)

Fmoc-Azy-OMe (2.56 g, 7.92 mmol) was dissolved in DCM (20 mL) and 2-methyl-3-buten-2-ol (20 mL). Molecular sieves (4 Å) were added, and under an inert gas atmosphere, BF_3_OEt_2_ (2.5 mL, 20 mmol, 2.5 equiv) was added dropwise into the solution over a period of 8 days. The solution was then filtered, evaporated to dryness, and loaded onto Celite (12 g). Purification by DCVC afforded Fmoc-Ser­(rPr)-OMe as a slowly crystallizing colorless oil (1.93 g, 60.2%).

ESI-HRMS: *m*/*z* 410.1956 [M + H]^+^ (calcd for C_24_H_28_NO_5_, 410.1962), 432.1775 [M + Na]^+^ (calcd for C_24_H_27_NNaO_5_, 432.1781); *R*_f_ = 0.62 (EtOAc/petroleum ether, 1:1 v/v); ^1^H NMR (500 MHz, DMSO-*d*_6_) δ 7.90 (d, *J* = 7.5 Hz, 2H), 7.74 (d, *J* = 7.1 Hz, 2H), 7.46–7.37 (m, 2H), 7.34 (tt, *J* = 7.4, 1.3 Hz, 2H), 5.80 (dd, *J* = 17.7, 10.8 Hz, 1H), 5.14 (dd, *J* = 17.7, 1.3 Hz, 1H), 5.11 (dd, *J* = 10.8, 1.3 Hz, 1H), 4.29 (m, 2H), 4.23 (m, 1H), 4.11 (q, *J* = 5.3 Hz, 1H), 3.64 (s, 3H), 3.18 (d, *J* = 5.3 Hz, 2H), 1.20 (s, 3H), 1.19 (s, 3H); ^13^C NMR (126 MHz, DMSO-*d*_6_) δ 171.0, 156.0, 143.8, 143.8, 143.5, 140.7, 127.7, 127.1, 125.3, 120.1, 114.1, 75.2, 65.8, 54.8, 51.9, 48.6, 46.6, 25.6, 25.3.

#### Fmoc-Ser­(rPr)-OH ((2*S*)-2-(((9*H*-Fluoren-9-ylmethoxy)­carbonyl)­amino)-3-((2-methylbut-3-en-2-yl)­oxy)­propanoic acid)

(1) By selective hydrolysis of Fmoc-Ser­(rPr)-OMe: Fmoc-Ser­(rPr)-OMe (220 mg, 0.538 mmol) was dissolved in THF (5.4 mL) and cooled to 0 °C. LiOH (26 mg, 1.76 mmol, 2 equiv) was dissolved in ice-cold H_2_O and added dropwise to the THF solution. The mixture was stirred for 2 h at 0 °C, then acidified by the addition of citric acid solution (10%, 10 mL) and extracted with DCM (5 × 15 mL). The pooled extracts were dried over MgSO_4_, filtered, and evaporated to dryness. After trituration with heptane, Fmoc-Ser­(rPr)-OH was obtained as a white crystalline solid (166 mg, 78% crude yield, 107 mg (50%) after flash chromatography).

(2) With reintroduction of the Fmoc protection group: to a solution of Fmoc-Ser­(rPr)-OMe (239 mg, 0.584 mmol) and 2-mercaptoethanol (81.5 μL, 1.17 mmol) in THF (6 mL), a solution of LiOH (56 mg, 2.34 mmol) in H_2_O (6 mL) was added dropwise at 0 °C. The mixture was stirred vigorously for 2 h, after which the pH was adjusted to 8.5 with 10% citric acid solution. Fmoc-OSu (197 mg, 0.584 mmol) was added in one portion, and the solution was stirred at RT for another 2 h. The reaction mixture was then acidified by the addition of 10% citric acid solution (10 mL) and extracted with EtOAc (3 × 10 mL). The combined organic layers were then extracted with 0.5% K_2_CO_3_ solution (5 × 10 mL). The aqueous extracts were combined and acidified to pH 2–3 with a 10% citric acid solution. After extraction with DCM (4 × 10 mL), drying over anhydrous MgSO_4_, and evaporation of solvents, Fmoc-Ser­(rPr)-OH was obtained as a white solid (185 mg, 80%).

ESI-HRMS: *m*/*z* 396.1801 [M + H]^+^ (calcd for C_23_H_26_NO_5_, 396.1806), 418.1620 [M + Na]^+^ (calcd for C_23_H_25_NNaO_5_, 418.1625); *R*_f_ = 0.40 (DCM/MeOH/AcOH, 9:1:0.02 v/v/v); ^1^H NMR (500 MHz, DMSO-*d*_6_) δ 12.74 (s, 1H), 7.89 (d, *J* = 7.5 Hz, 2H), 7.75 (d, *J* = 7.5 Hz, 2H), 7.51 (d, *J* = 8.4 Hz, 1H), 7.42 (t, *J* = 7.3 Hz, 2H), 7.33 (t, *J* = 7.5 Hz, 2H), 5.82 (dd, *J* = 17.7, 10.8 Hz, 1H), 5.14 (dd, *J* = 17.6, 1.3 Hz, 1H) 5.09 (dd, *J* = 10.8, 1.2 Hz, 1H), 4.29–4.19 (m, 3H), 4.12 (dt, *J* = 8.4, 5.3 Hz, 1H), 3.51 (qd, *J* = 9.5, 5.4 Hz, 2H), 1.20 (d, *J* = 2.4 Hz, 6H); ^13^C NMR (126 MHz, DMSO) δ 171.8, 156.0, 143.8, 143.7, 140.7, 127.6, 127.1, 125.4, 120.1, 114.0, 75.1, 65.8, 62.2, 54.8, 46.6, 25.7, 25.3.

### X-ray Crystallography

X-ray data from single crystals mounted in a lithographic cryo-loop were acquired at 101(2) K for mollamide C·H_2_O or at 100(2) K for keenamide A·1.5 H_2_O, respectively, using a Rigaku Synergy R diffractometer, equipped with a Micromax007HF rotating anode source generating mirror-monochromated Cu Kα radiation (λ = 1.54184 Å), a kappa goniometer, and a HyPix-Arc 150° detector. Data collection and reduction were performed using the program CrysAlisPro (Rigaku Oxford Diffraction, Yarton, UK). Multiscan absorption corrections were carried out with ABSPACK in CrysAlisPro. The crystal structures were solved with olex2.solve^36^ and refined with SHELXL2019/3.^37^ Anisotropic atomic displacement parameters (ADPs) were introduced for all non-hydrogen atoms. Hydrogen atoms were placed in geometrically calculated positions and refined with the appropriate riding model, except for the amide and water hydrogen atoms in mollamide C·H_2_O, which were refined semifreely with appropriate distance restraints on the X–H bond lengths. The water hydrogen atoms in keenamide A·1.5 H_2_O could not be located reliably in difference Fourier maps and were therefore excluded from the structural model. An iso-butyl group in keenamide A·1.5 H_2_O and a prenyl group in mollamide C·H_2_O exhibit positional disorder, which was accounted for by split models. The occupancy ratios were each refined by means of a free variable. Similar distance restraints, similarity restraints, and restraints toward isotropy were applied on ADPs (see Supplementary Crystallographic Data). The absolute structures were assigned based on the known absolute configuration of the starting materials in the synthesis and confirmed by Flack x parameters close to zero (Parsons’ quotient method).^38^ Crystal data and refinement details are listed in Table S6. Crystallographic data for the structures reported in this paper have been deposited with the Cambridge Crystallographic Data Centre (keenamide A·1.5 H_2_O, CCDC 2421229; mollamide C·H_2_O; CCDC 2421230). Copies of the data can be obtained, free of charge, on application to the Director, CCDC, 12 Union Road, Cambridge CB2 1EZ, UK (fax: +44-(0)­1223-336033 or e-mail: deposit@ccdc.cam.ac.uk), or via www.ccdc.cam.ac.uk/structures.

## Supplementary Material



## Notes

NMR spectra (^1^H, ^13^C for all compounds; COSY, HMBC for cyclopeptides); MS/MS spectra of keenamide A and mollamide C with additional information on MS experiments; HPLC traces of cyclopeptides, spectral comparison of natural keenamide A, and synthetic keenamide stereoisomers; and crystal data and refinement details for keenamide A and mollamide C (PDF)

## ⊥

Freie Universität Berlin, Institute of Pharmacy, Department of Pharmaceutical Biology, Königin-Luise-Straße 2+4, 14195 Berlin, Germany

## Notes

NMR raw data of all described compounds were uploaded to nmrXive (10.57992/nmrxiv.p92); MS/MS spectra of keenamide A and mollamide C are available as MassIVE data sets MSV000095644 (doi:10.25345/C5R49GM9B) and SV000095643 (doi:10.25345/C5VX06F4T).
