# Supplementary material for: Total Synthesis and Structural Revision of Keenamide A
Source: J Nat Prod. 2026 Jan 5;89(2):483–91. doi: 10.1021/acs.jnatprod.5c01325 (PMC12954850; doi:10.1021/acs.jnatprod.5c01325)
Supplement: Supplementary file 1 [file np5c01325_si_001.pdf]

Supporting Information to

Total Synthesis and Structural Revision of Keenamide A

*Lukas Koch[a], Christoph Wiedemann[b], Christoph Parthier[c], Rüdiger W. Seidel[d], Milton T. Stubbs[c], Mike Schutkowski[a], Marat Meleshin[a]**

[a] Martin Luther University Halle-Wittenberg, Institute of Biochemistry and Biotechnology, Department of Enzymology, Charles Tanford Protein Center, Kurt-Mothes-Straße 3a, 06120 Halle, Germany;

[b] Friedrich Schiller University Jena, Institute of Organic Chemistry and Macromolecular Chemistry, Humboldtstraße 10, 07743 Jena, Germany;

[c] Martin Luther University Halle-Wittenberg, Institute of Biochemistry and Biotechnology, Department of Physical Biotechnology, Charles Tanford Protein Center, Kurt-Mothes-Straße 3a, 06120 Halle, Germany;

[d] Martin Luther University Halle-Wittenberg, Institute of Pharmacy, Wolfgang-Langenbeck-Straße 4, 06120 Halle (Saale), Germany.

*Email: marat.meleshin@biochemtech.uni-halle.de

Table of Contents

Supplementary data for methyl 1-tritylaziridine-2-carboxylate (Trt-Azy-OMe) S2

Supplementary data for 1-(9*H*-fluoren-9-ylmethyl) 2-methyl aziridine-1,2-dicarboxylate (Fmoc-Azy-OMe) S4

Supplementary data for methyl (2S)-2-(((9*H*-fluoren-9-ylmethoxy)carbonyl)amino)-3-((2-methylbut-3-en-2-yl)oxy)propanoate (Fmoc-Ser(rPr)-OMe) S6

Supplementary data for ((2S)-2-(((9*H*-fluoren-9-ylmethoxy)carbonyl)amino)-3-((2-methylbut-3-en-2-yl)oxy)propanoic acid (Fmoc-Ser(rPr)-OH) S8

Supplementary data for cyclo[Ser(rPr)-Leu-Tzn-Ile-Pro-Gly] (1, originally proposed structure of keenamide A) S10

Supplementary data for cyclo[Ser(rPr)-Leu-D-Tzn-Ile-Pro-Gly] (2, keenamide A) S17

Supplementary data for cyclo[Ser(rPr)-D-Leu-Tzn-Ile-Pro-Gly] (3) S26

Supplementary data for cyclo[Ser(rPr)-D-Leu-D-Tzn-Ile-Pro-Gly] (4) S34

Supplementary data for cyclo[Ser(rPr)-Leu-Tzl-Ile-Pro-Gly] (mollamide C) S41

Comparison of chemical shifts of keenamide stereoisomers with original spectral data S49

Supplementary crystallographic data S51

References S52

* meleshin@biochemtech.uni-halle.de

Supplementary data for methyl 1-tritylaziridine-2-carboxylate (Trt-Azy-OMe)

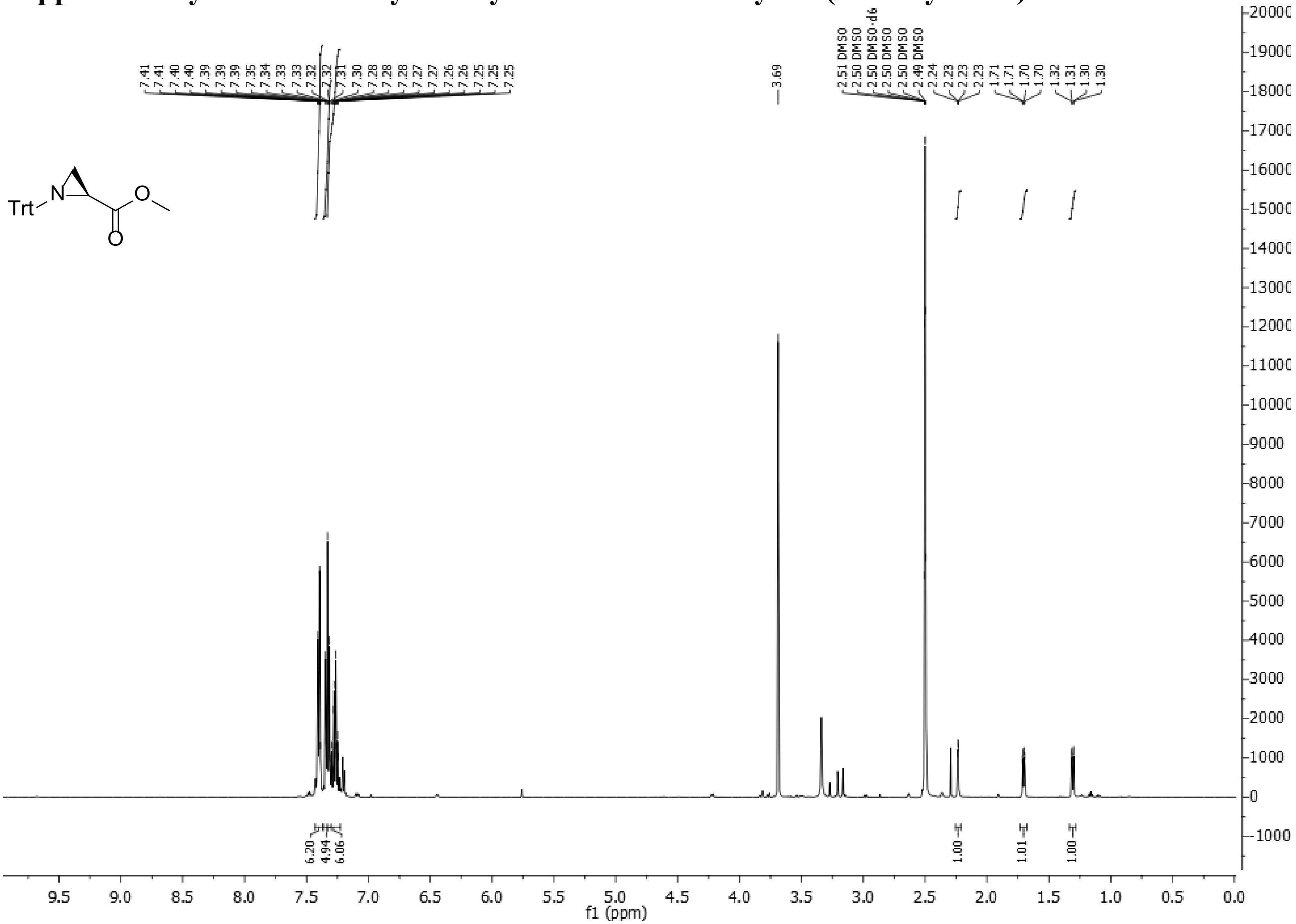

Figure S1 ^{1}H NMR spectrum of methyl 1-tritylaziridine-2-carboxylate (DMSO-*d6*, 500 MHz).

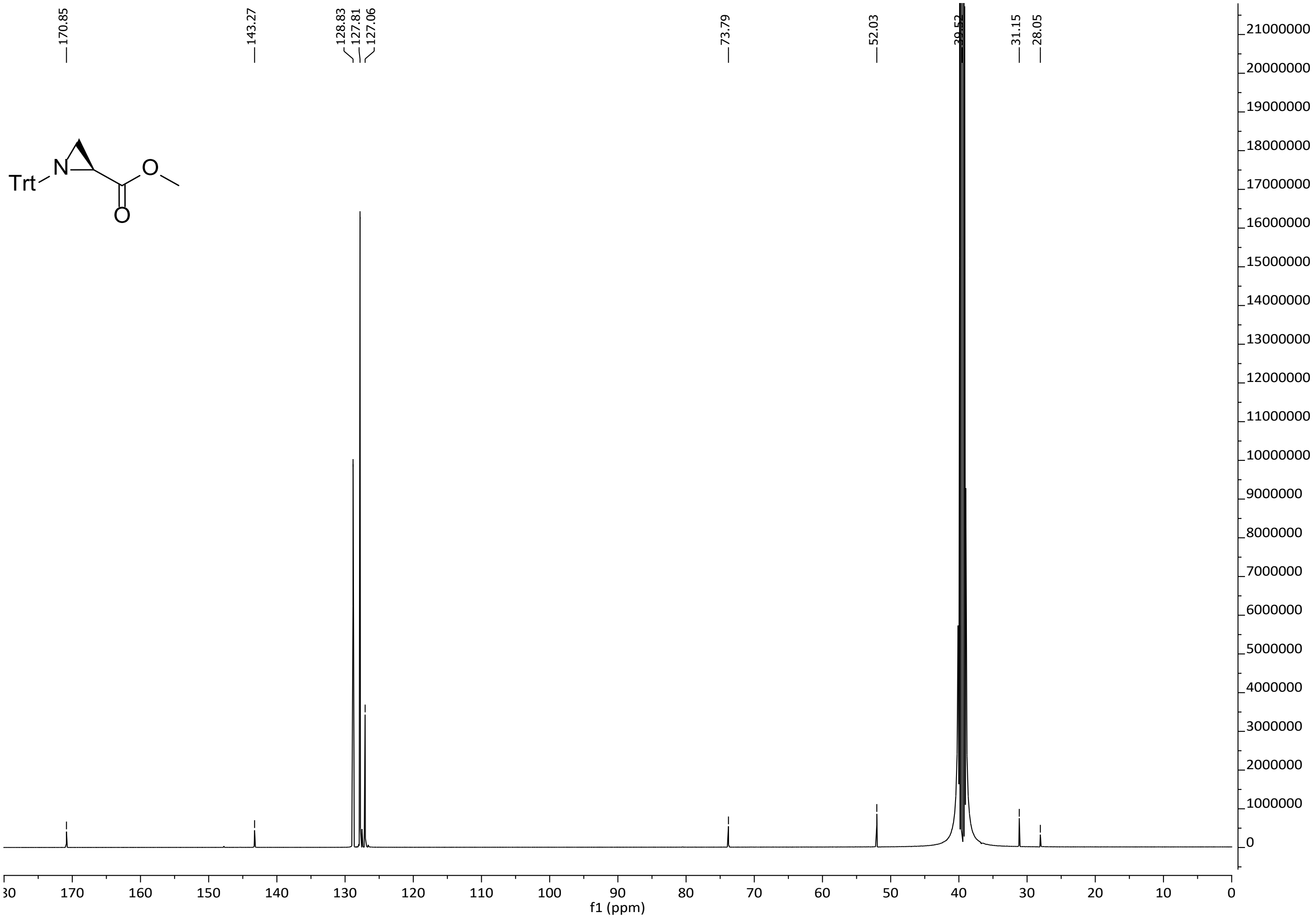

Figure S2 ^{13}C NMR spectrum of 1-tritylaziridine-2-carboxylate (DMSO-*d6*, 126 MHz)

Supplementary data for 1-(9*H*-fluoren-9-ylmethyl) 2-methyl aziridine-1,2-dicarboxylate (Fmoc-Azy-OMe)

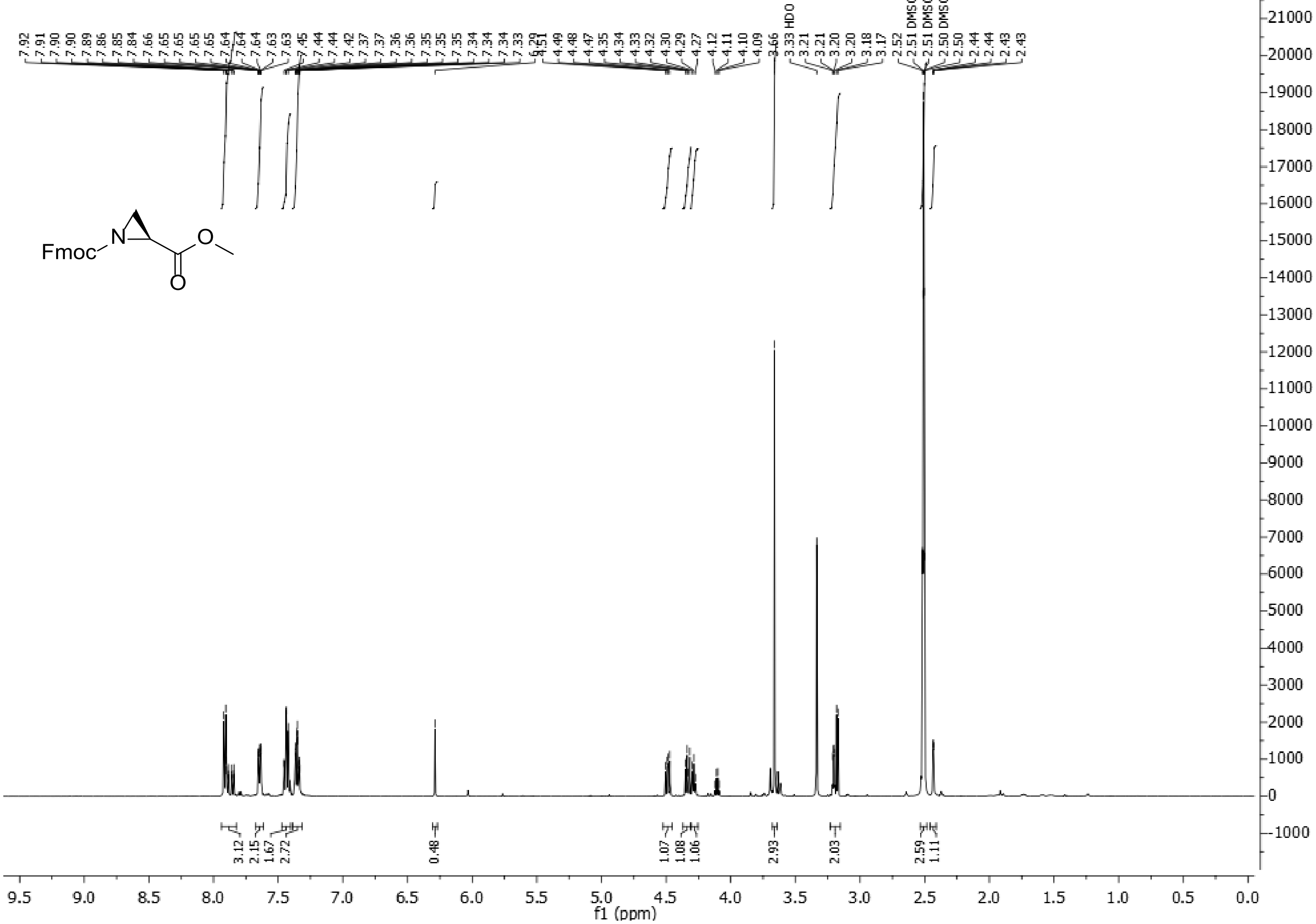

Figure S3 ^{1}H NMR spectrum of 1-(9*H*-fluoren-9-ylmethyl) 2-methyl aziridine-1,2-dicarboxylate (DMSO-*d*6, 500 MHz).

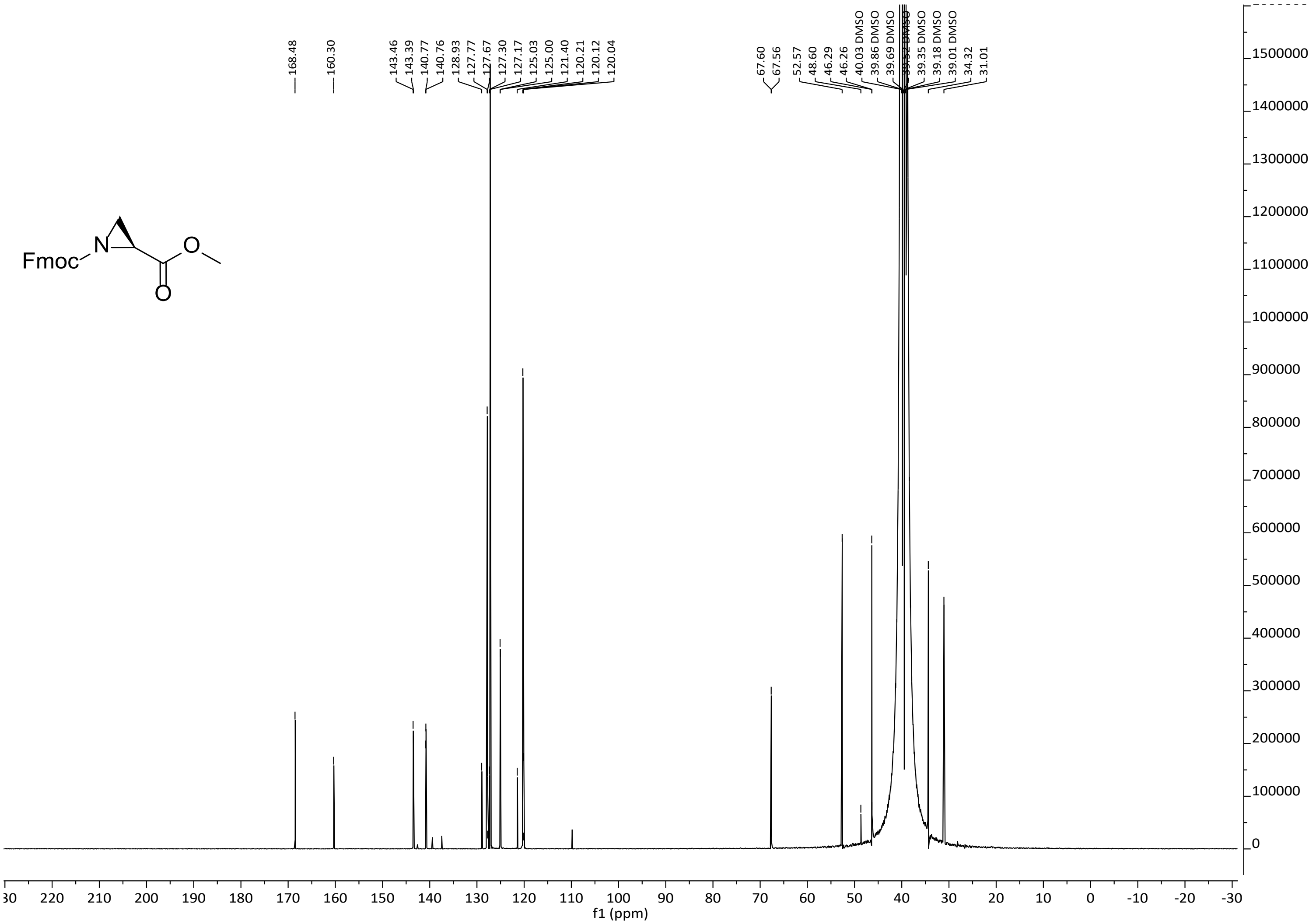

Figure S4 ^{13}C NMR spectrum of 1-(9*H*-fluoren-9-ylmethyl) 2-methyl aziridine-1,2-dicarboxylate (DMSO-*d*6, 126 MHz).

Supplementary data for methyl (2S)-2-(((9*H*-fluoren-9-ylmethoxy)carbonyl)amino)-3-((2-methylbut-3-en-2-yl)oxy)propanoate (Fmoc-Ser(rPr)-OMe)

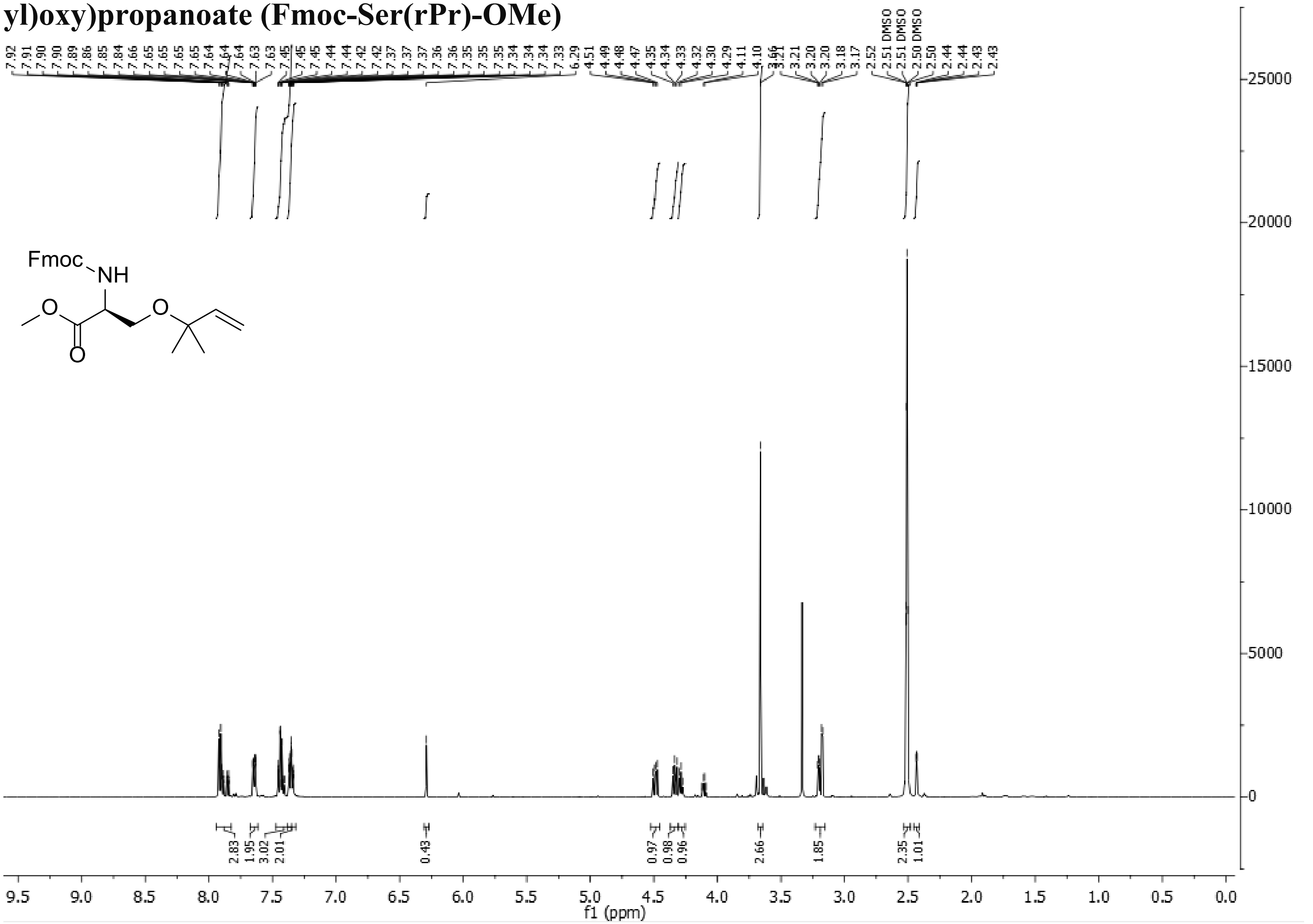

Figure S5 ^{1}H NMR spectrum of methyl (2*S*)-2-(((9*H*-fluoren-9-ylmethoxy)carbonyl)amino)-3-((2-methylbut-3-en-2-yl)oxy)propanoate (DMSO-*d*6, 500 MHz).

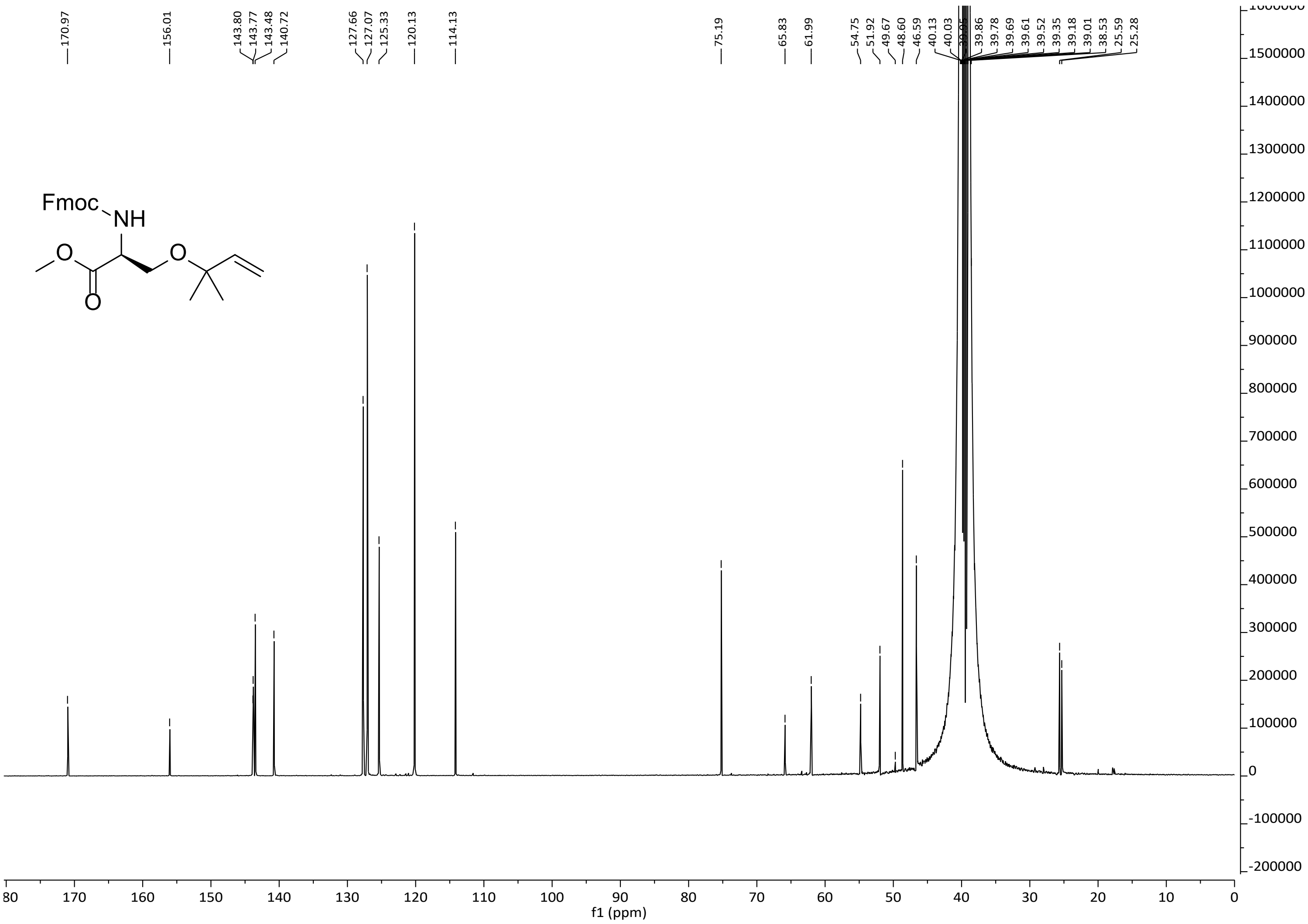

Figure S6 ^{13}C NMR spectrum of methyl (*2S*)-2-(((9*H*-fluoren-9-ylmethoxy)carbonyl)amino)-3-((2-methylbut-3-en-2-yl)oxy)propanoate (DMSO-*d6*, 126 MHz).

Supplementary data for ((2S)-2-(((9*H*-fluoren-9-ylmethoxy)carbonyl)amino)-3-((2-methylbut-3-en-2-yl)oxy)propanoic acid (Fmoc-Ser(rPr)-OH)

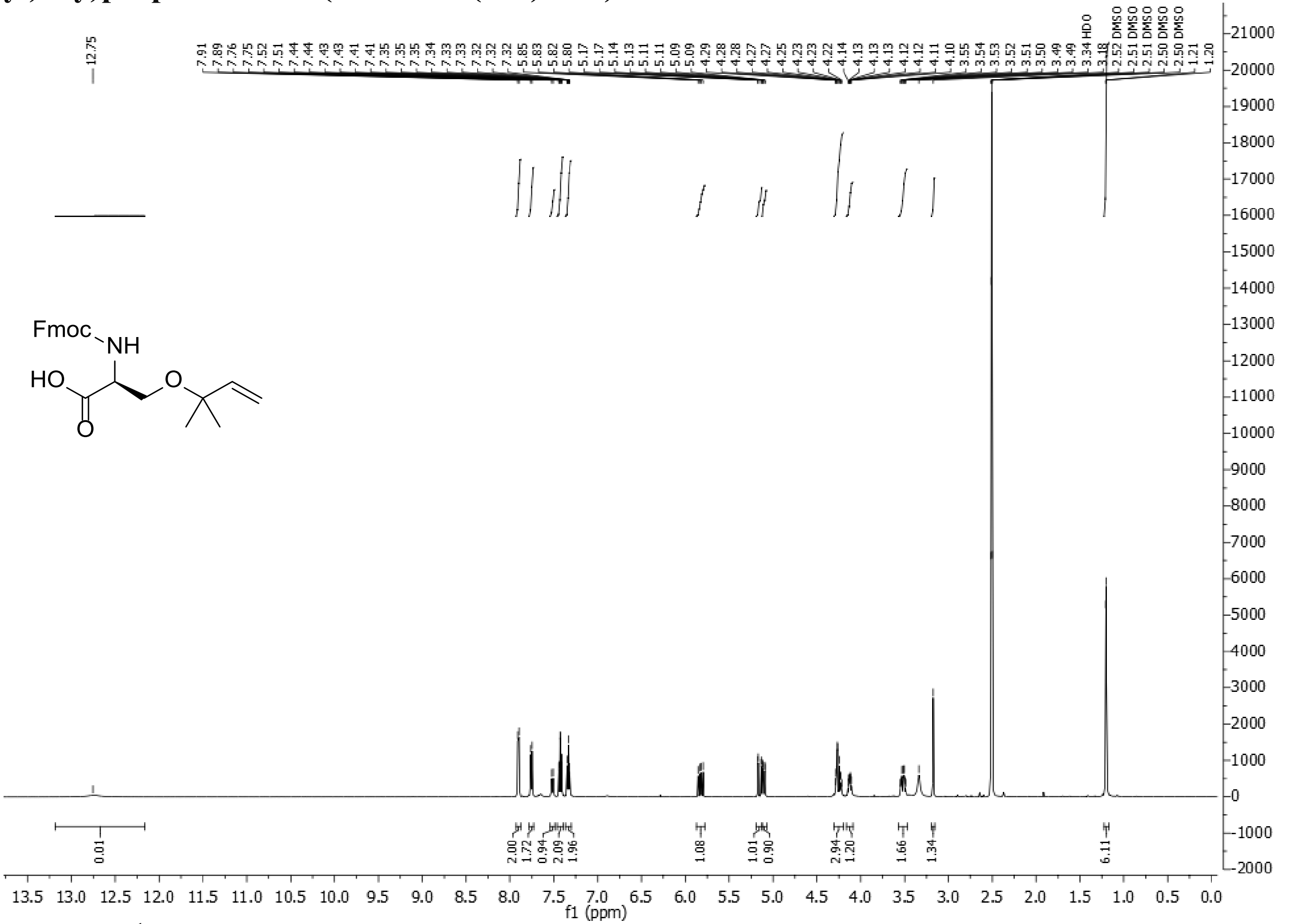

Figure S7 ^{1}H NMR spectrum of ((2*S*)-2-(((9*H*-fluoren-9-ylmethoxy)carbonyl)amino)-3-((2-methylbut-3-en-2-yl)oxy)propanoic acid (DMSO-*d6*, 500 MHz).

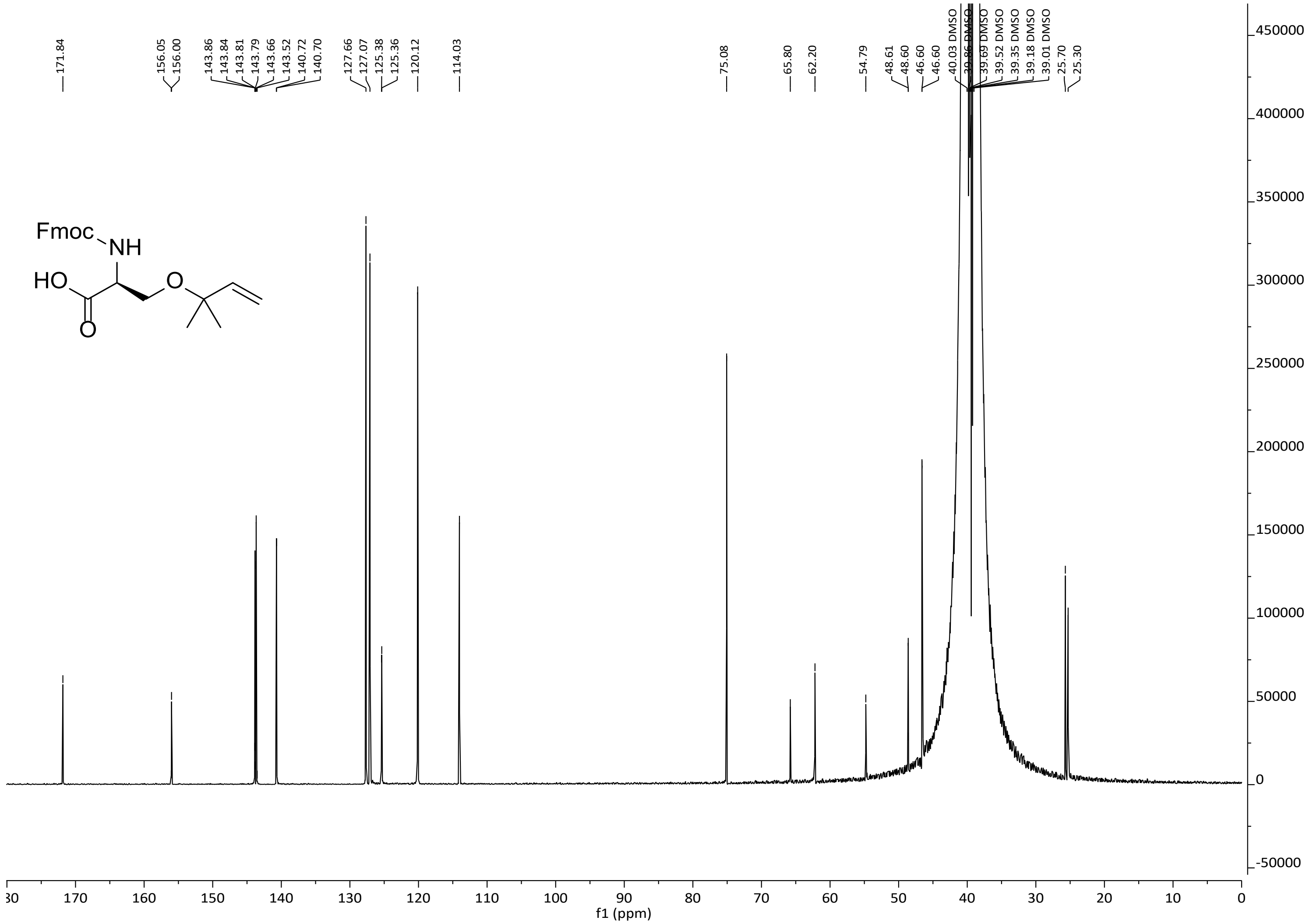

Figure S8 ^{13}C NMR spectrum of ((*2S*)-2-(((9*H*-fluoren-9-ylmethoxy)carbonyl)amino)-3-((2-methylbut-3-en-2-yl)oxy)propanoic acid (DMSO-*d6*, 126 MHz)

Supplementary data for cyclo[Ser(rPr)-Leu-Tzn-Ile-Pro-Gly] (1, originally proposed structure of keenamide A)

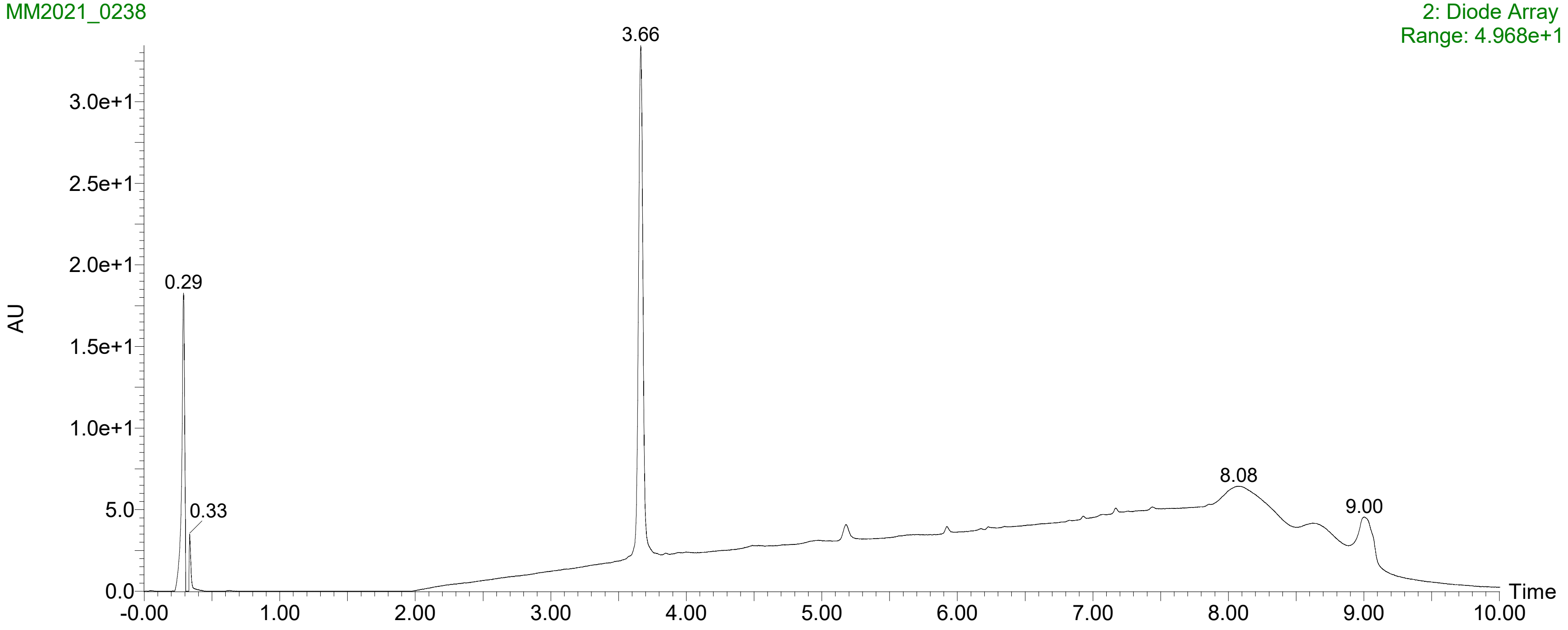

Figure S9 HPLC trace of **1**. 5-100% MeCN/H_2O + 0.1% formic acid / 10 min, Waters X Bridge C8 column (2.5 µm, 2.1 x 100 mm).

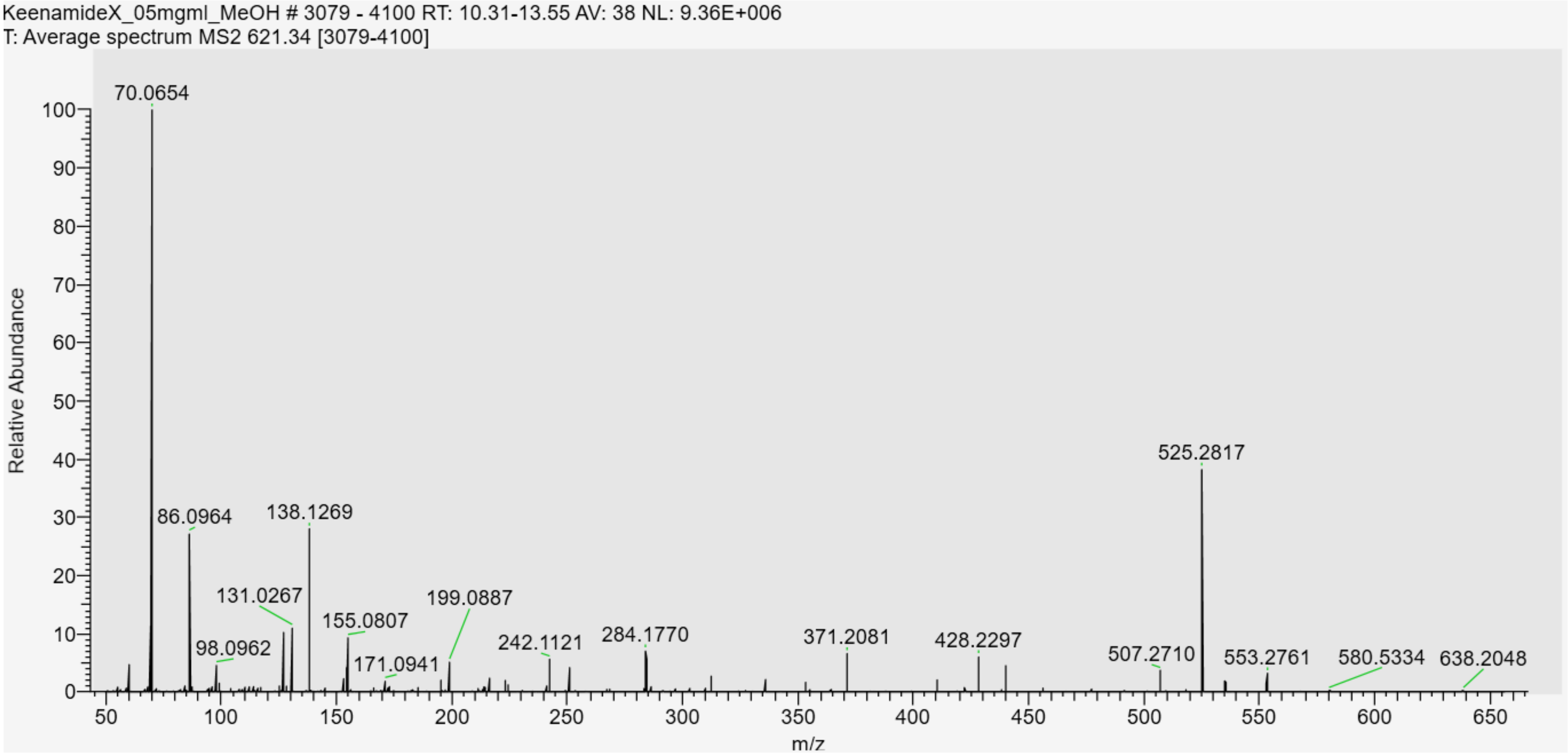

Figure S10 MS/MS spectrum of **1**. Spectrum was recorded on a Q Exactive Plus Orbitrap mass spectrometer; precursor selection window was set to 1.5 m/z; CE was set to 25, 50, 75, resolution was set to 35,000.

Table S1 $^1H^a$- and $^{13}C^b$-NMR Data ($CDCl_3$) for **1**

position		δ_C	δ_H (*J* in Hz)
Gly	1	169.2	
	2a	43.1	4.48, dd (17.4, 9.6)
	2b		3.45, m
	NH		7.54, dd, (9.6, 3.6)
Pro	1	172.1	
	2	62.5	4.13, dd (9.6, 7.0)
	3a	29.0	2.22, m
	3b		2.00, m
	4a	25.9	2.12 dt, (12.3, 6.3)
	4b		1.80, m
	5a	48.0	3.67, t (8.9)
	5b		3.57, td (10.3, 6.2)
Ile	1	169.8	
	2	55.3	4.57, dd (7.0, 3.9)
	3	39.1	1.85, m
	4	15.9	0.85, t (7.3)
	5a	24.3	1.98, m
	5b		1.31, m
	6	11.9	0.91, d (6.9)
	NH		6.87, d (7.0)
Tzn	1	170.2	
	2	78.7	5.12, dd (10.5, 5.0)
	3a	37.8	3.73, t (11.0)
	3b		3.47, m
Leu	1	178.1	
	2	49.5	5.37, td (9.1, 6.5)
	3	42.5	1.61, m
	4	25.2	1.67, m
	5	22.7	0.96, d (6.5)
	6	22.3	0.93, d (6.5)
	NH		8.14, d (9.3)
Ser	1	170.7	
	2	51.8	4.62, ddd (11.5, 7.6, 4.6)
	3a	61.5	3.55, dd (8.8, 4.8)
	3b		3.16, d (8.8, 11.2)
	NH		8.34, d (7.6)
prenyl	1	77.1	
	2	25.9	1.35, s
	3	25.8	1.32, s
	4	142.1	5.85, dd (17.5, 10.8)
	5a	115.4	5.18, d (17.5)
	5b		5.16, d (10.8)

^{a} at 700 MHz, $CHCl_3$ signal at 7.26 ppm, 293.2 K; ^{b}at 176 MHz, $CDCl_3$ signal at 77.16 ppm, 293.2 K

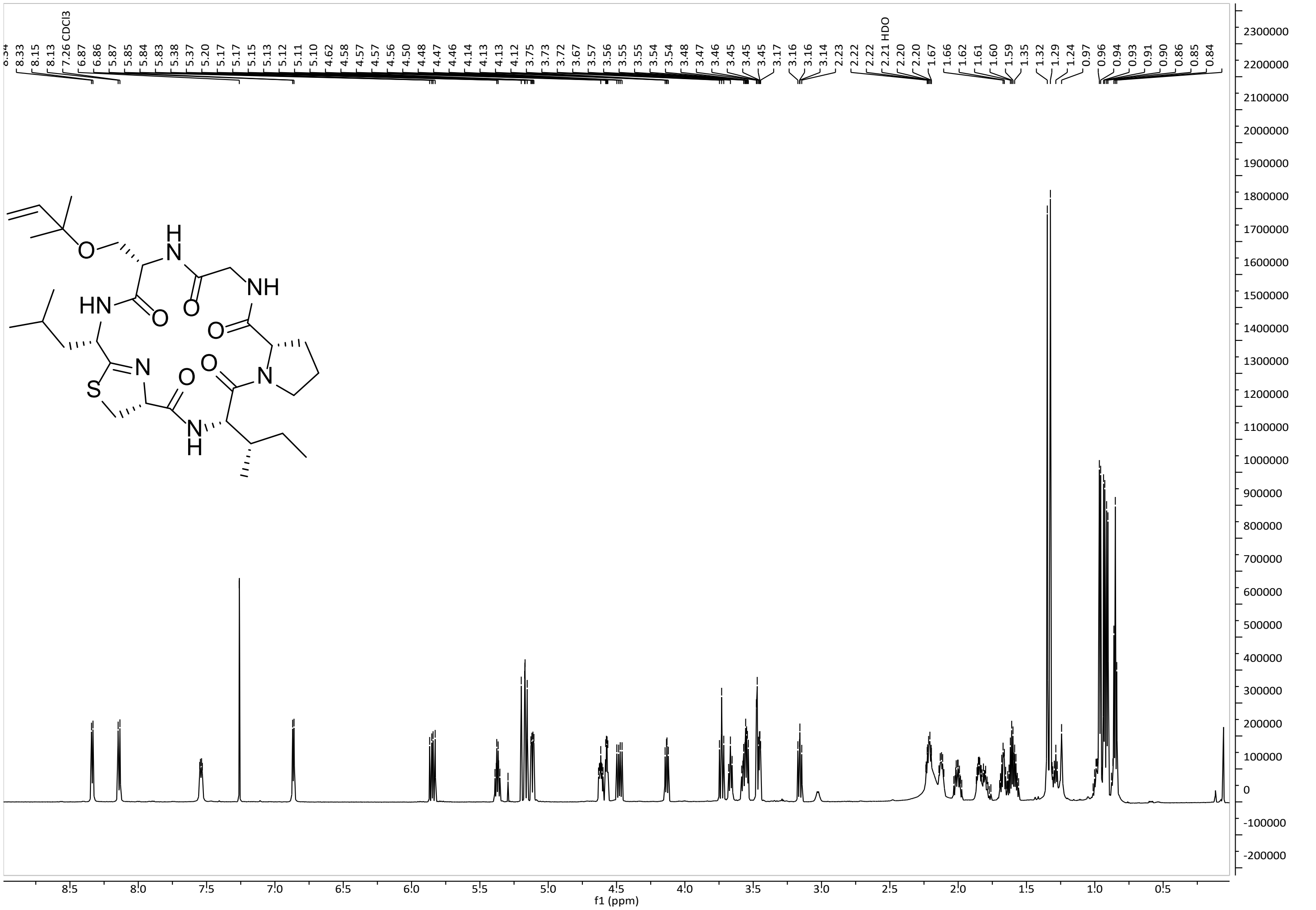

Figure S11 ¹H NMR spectrum of **1**. ($CDCl_3$, 700 MHz).

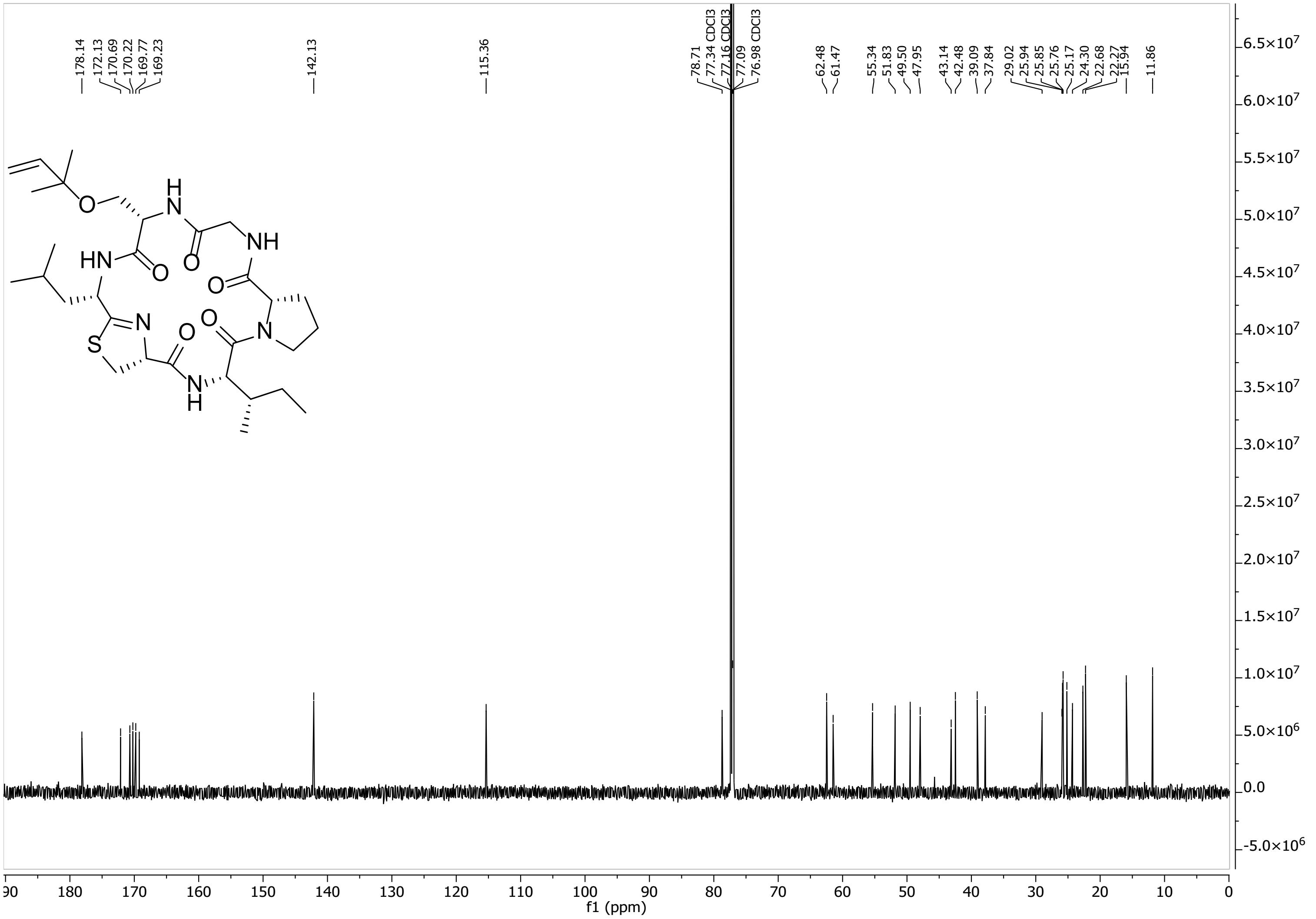

Figure S12 ^{13}C NMR spectrum of **1** ($CDCl_3$, 176 MHz).

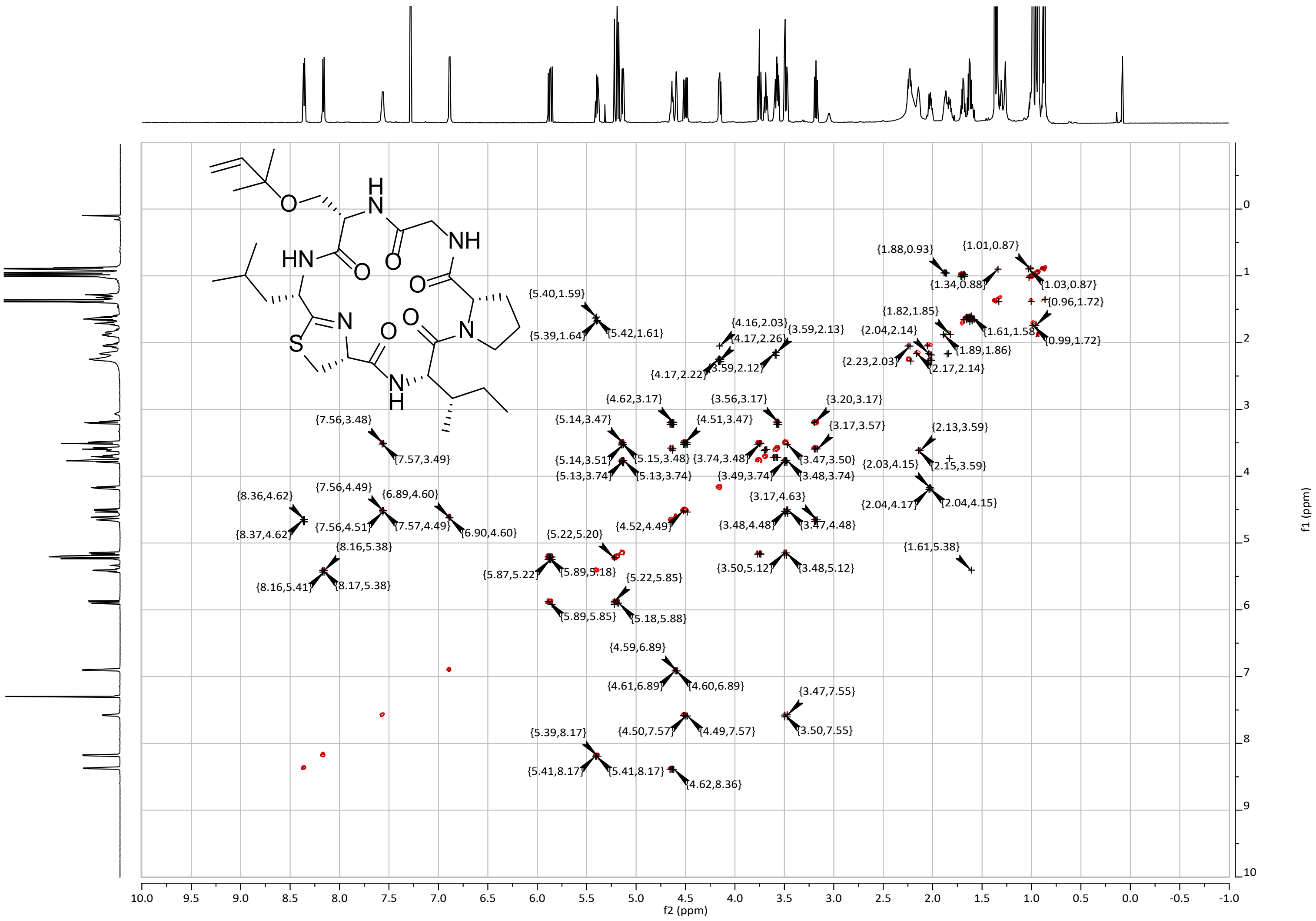

Figure S13 1H-1H COSY spectrum of **1** ($CDCl_3$, 700 MHz).

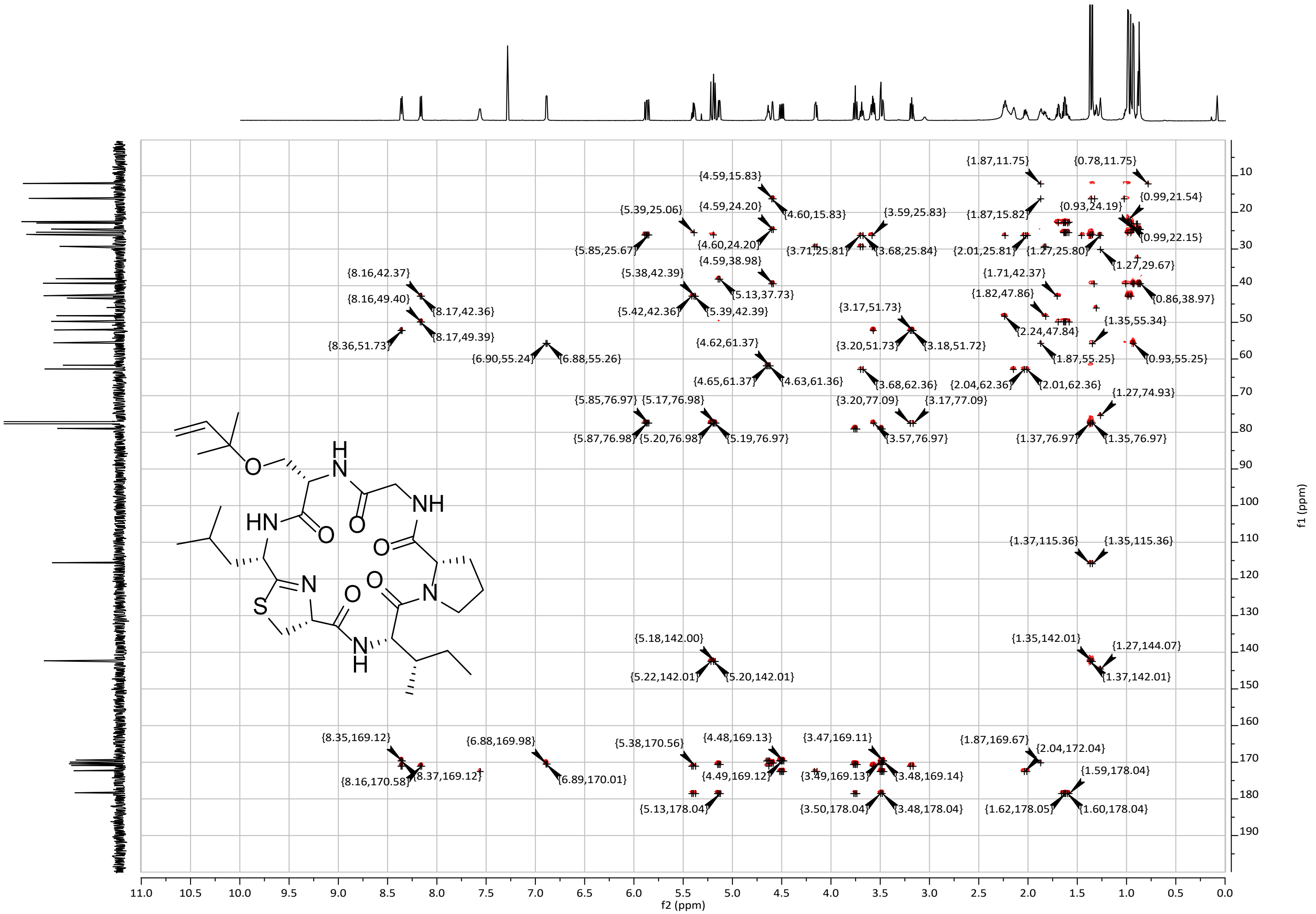

Figure S14 ¹H-¹³C HMBC spectrum of **1** ($CDCl_3$, 700 MHz).

Supplementary data for cyclo[Ser(rPr)-Leu-D-Tzn-Ile-Pro-Gly] (2, keenamide A)

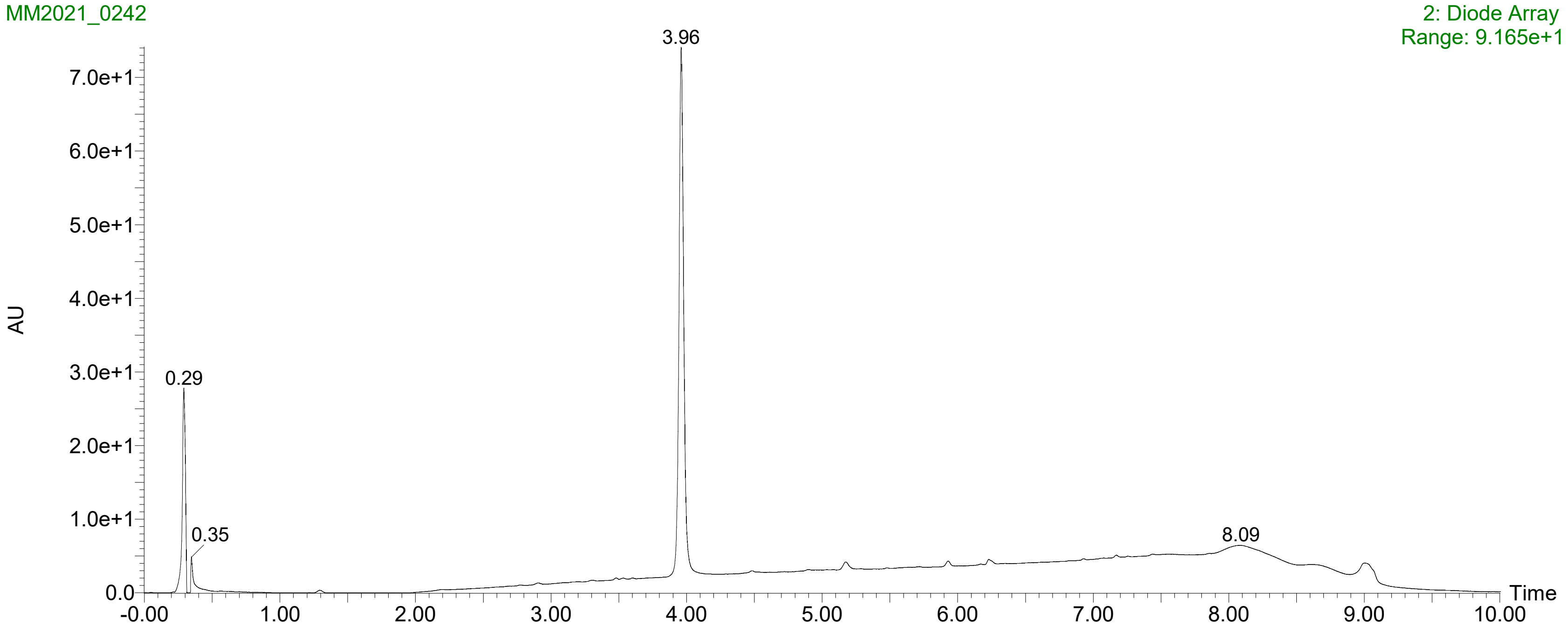

Figure S15 HPLC trace of **2**. 5-100% MeCN/H_2O + 0.1% formic acid / 10 min, Waters X Bridge C8 column (2.5 µm, 2.1 x 100 mm).

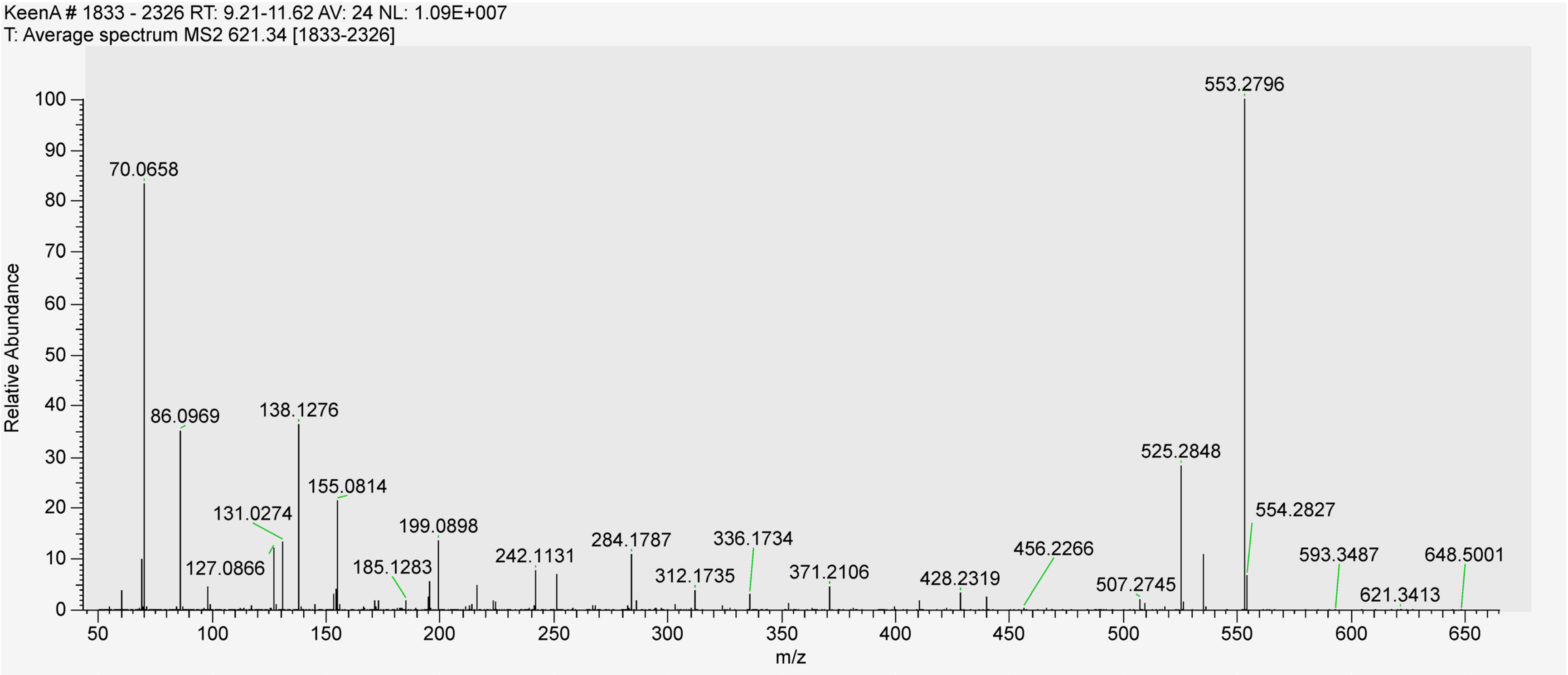

Figure S16 MS/MS spectrum of **2** (keenamide A). Spectrum was recorded on a Q Exactive Plus Orbitrap mass spectrometer; precursor selection window was set to 1.5 m/z; CE was set to 25, 50, 75, resolution was set to 35,000.

Table S2 $^{1}H^{a}$- and $^{13}C^{b}$-NMR ($CDCl_3$) Data for **2** (keenamide A)

position		δ_C	δ_H (*J* in Hz)
Gly	1	169.0	
	2a	43.14	4.56, ddd, (17.4, 10.0, 3.2)
	2b		3.43, m
	NH		7.10[c], ddd (17.4, 10.0, 2.6)
Pro	1	171.7	
	2	62.8	4.1, dd (10.1, 6.7)
	3a	29.0	2.19, m
	3b		2.03, m
	4a	25.9	2.14, m
	4b		1.87, m
	5a	47.7	3.67, m
	5b		3.58, m
Ile	1	170.0	(NH)
	2	55.5	4.66, dd (8.9, 3.3)
	3	37.3	1.90, m
	4	16.4	1.02, d (6.7)
	5	23.0	1.29, m
			0.98, m
	6	11.7	0.86, t (7.3)
	NH		7.70, d (8.9)
Tzn	1	171.2	
	2	78.1	5.03, t (9.7)
	3	36.3	3.67, m
			3.58, m
Leu	1	175.9	(NH)
	2	51.0	4.82, m
	3	42.6	1.62, m
			1.53, m
	4	25.4	1.78, m
	5	23.4	0.97, d (6.7)
	6	21.4	0.94, d (6.5)
	NH		8.17, d (8.5)
Ser	1	171.1	(NH)
	2	50.8	4.77, m
	3	62.6	3.45, m
	NH		8.59, d (8.8)
prenyl	1	77.1	
	2	26.4	1.32, s
	3	25.3	1.34, s
	4	142.7	5.90, dd (17.5, 10.8)
	5a	115.1	5.18, d (17.5)
	5b		5.14, d (10.8)

[a]at 700 MHz, $CHCl_3$ signal at 7.26 ppm, 293.2 K; [b]at 176 MHz, $CDCl_3$ signal at 77.16 ppm, 293.2 K; [c]variable chemical shifts were observed in different preparations.

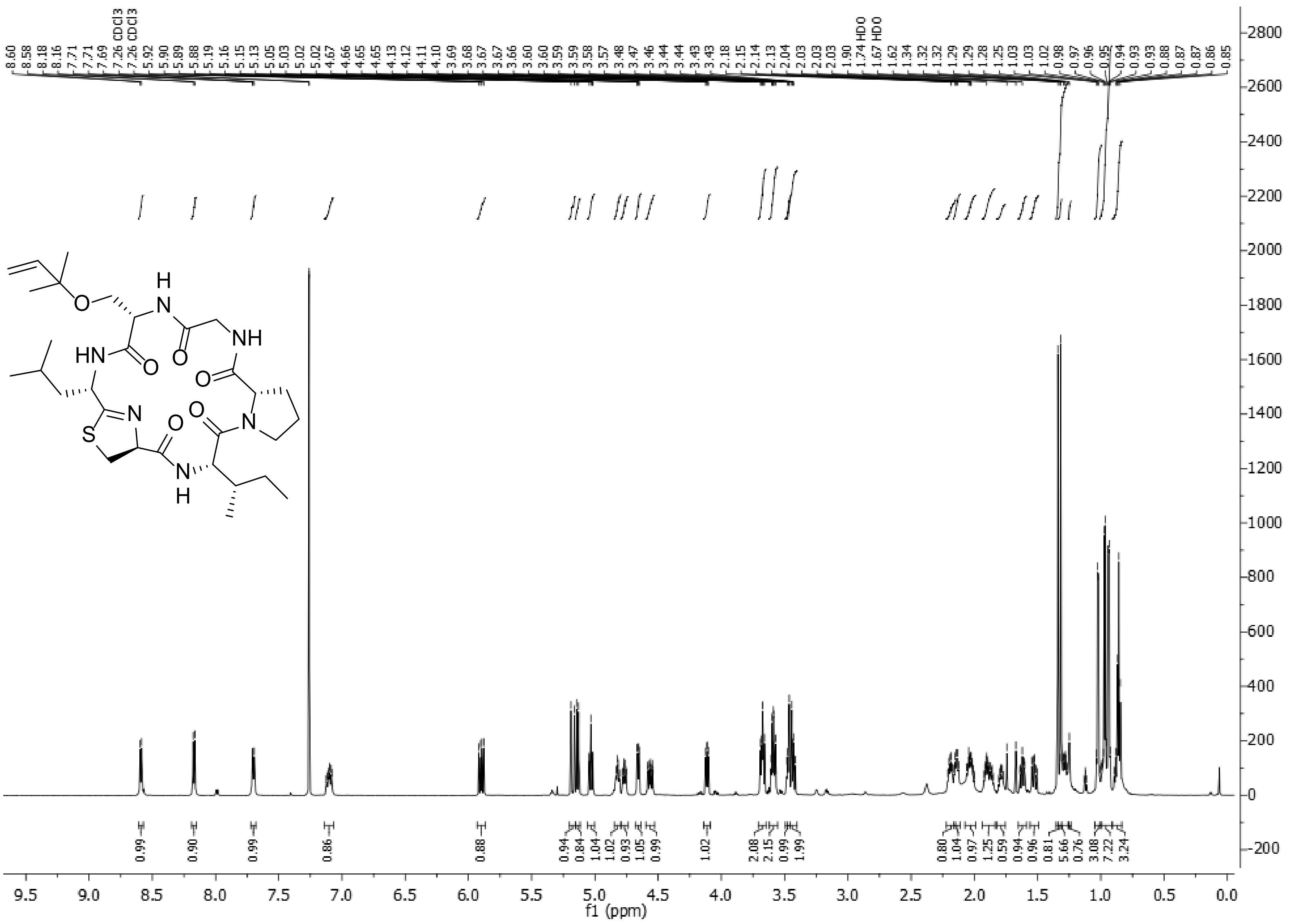

Figure S17 ^{1}H NMR spectrum of **2**, prepared by macrocyclization/desulfhydration ($CDCl_3$, 500 MHz).

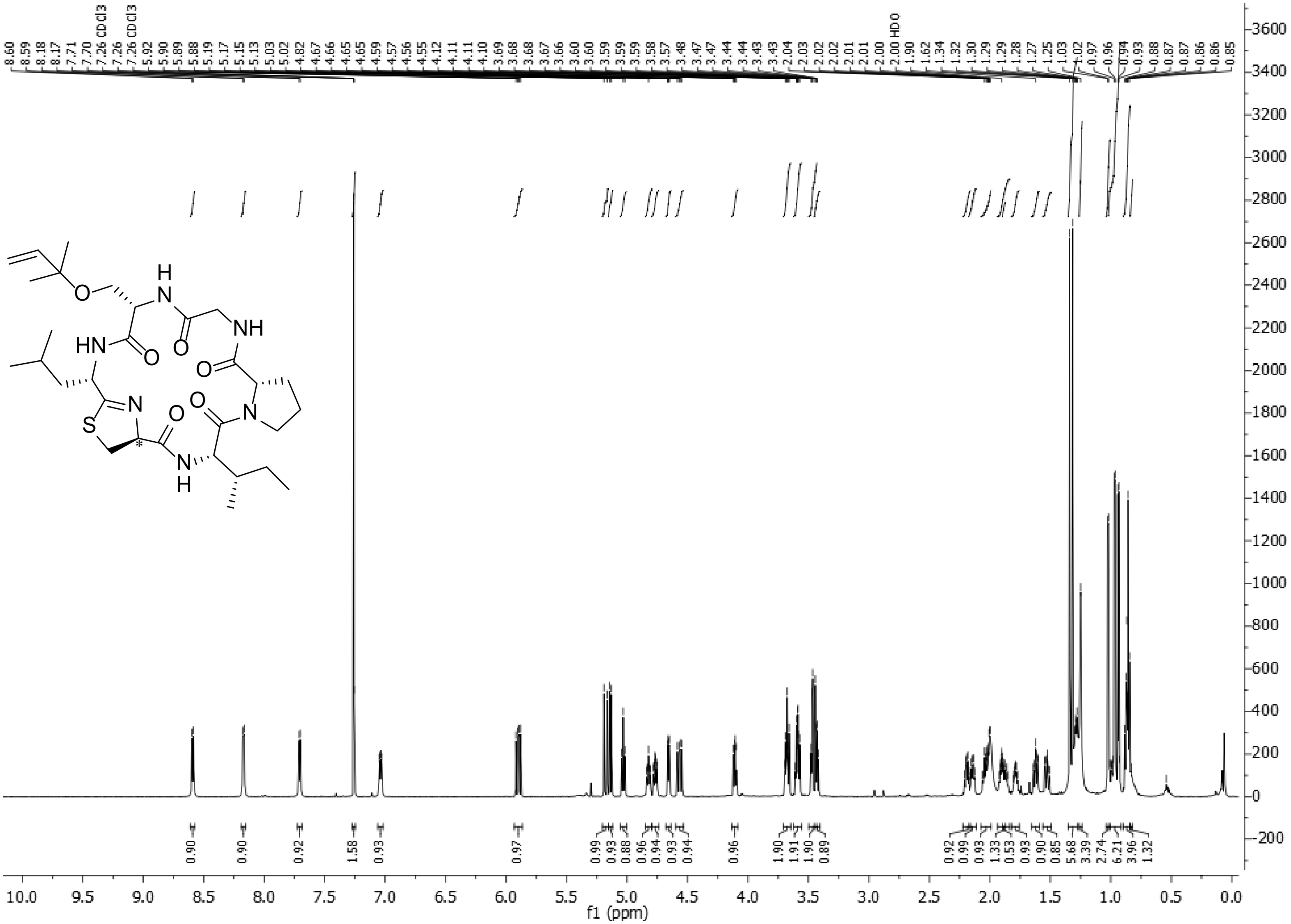

Figure S18 ^{1}H NMR spectrum of **2**, prepared by epimerization of **1** with DBU ($CDCl_3$, 700 MHz). The epimerized stereocenter is marked with an asterisk.

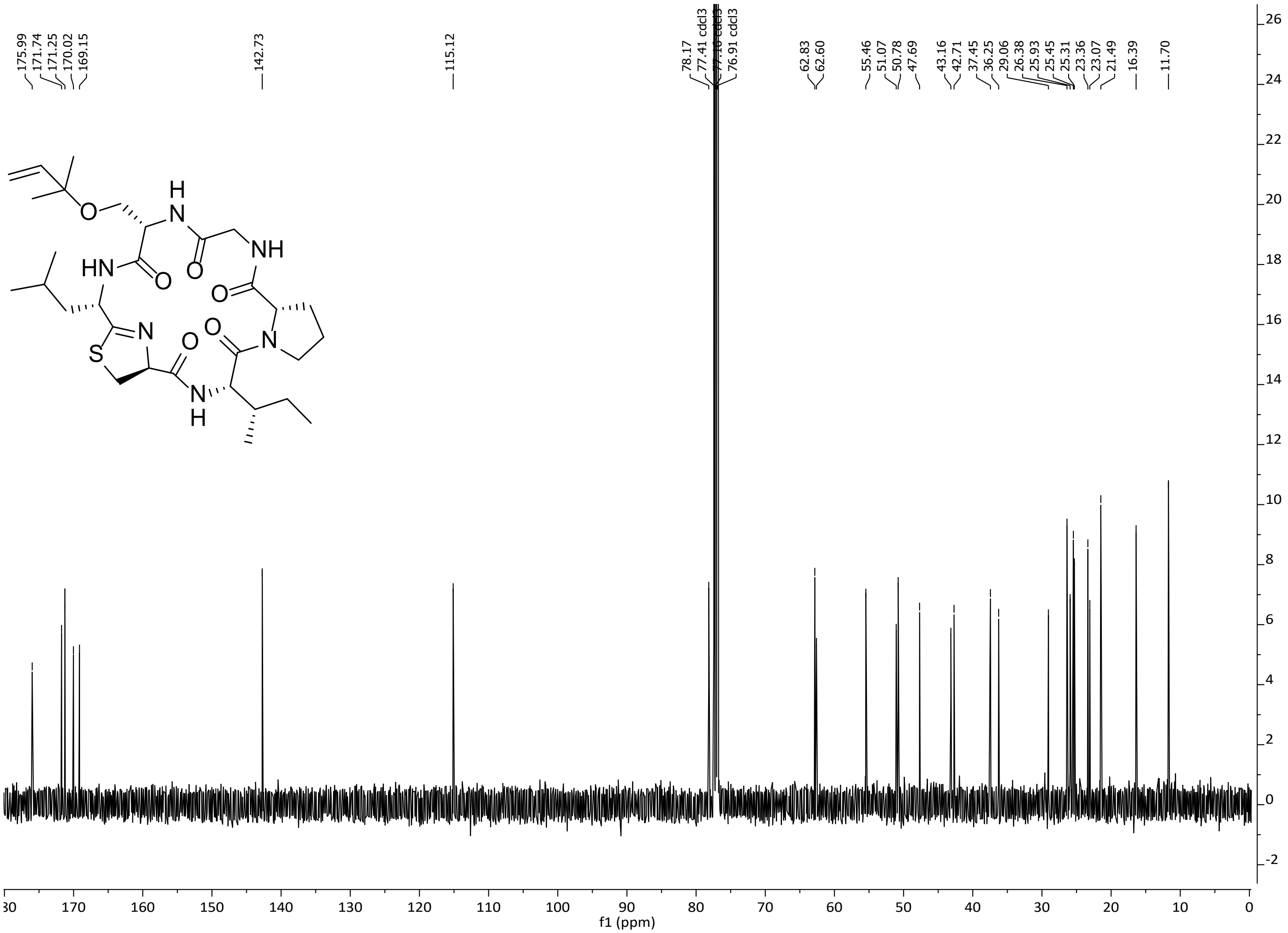

Figure S19 ^{13}C NMR spectrum of **2**, prepared by macrocyclization/desulfhydration ($CDCl_3$, 125 MHz).

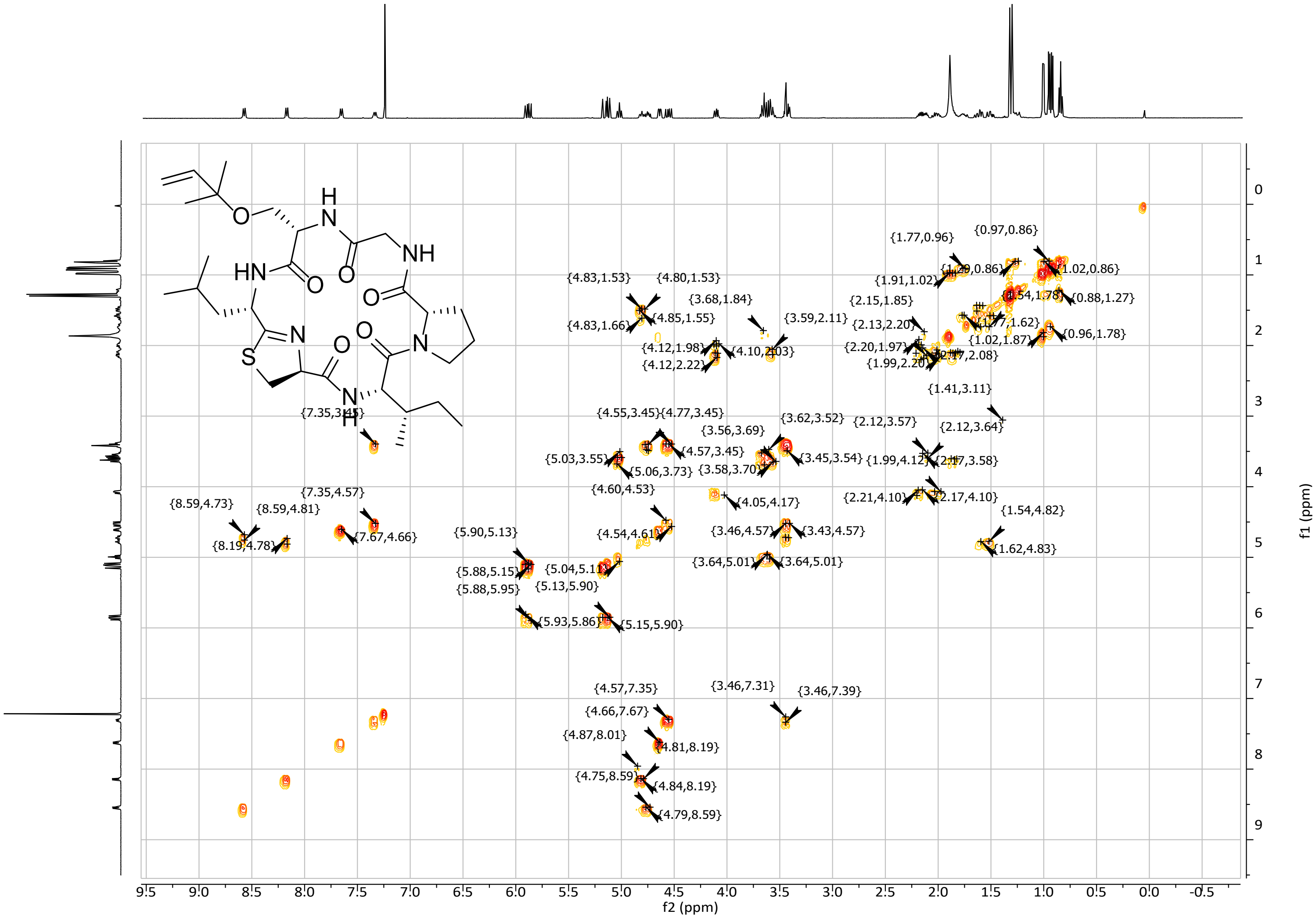

Figure S20 ^{1}H-^{1}H COSY spectrum of **2** ($CDCl_3$, 500 MHz).

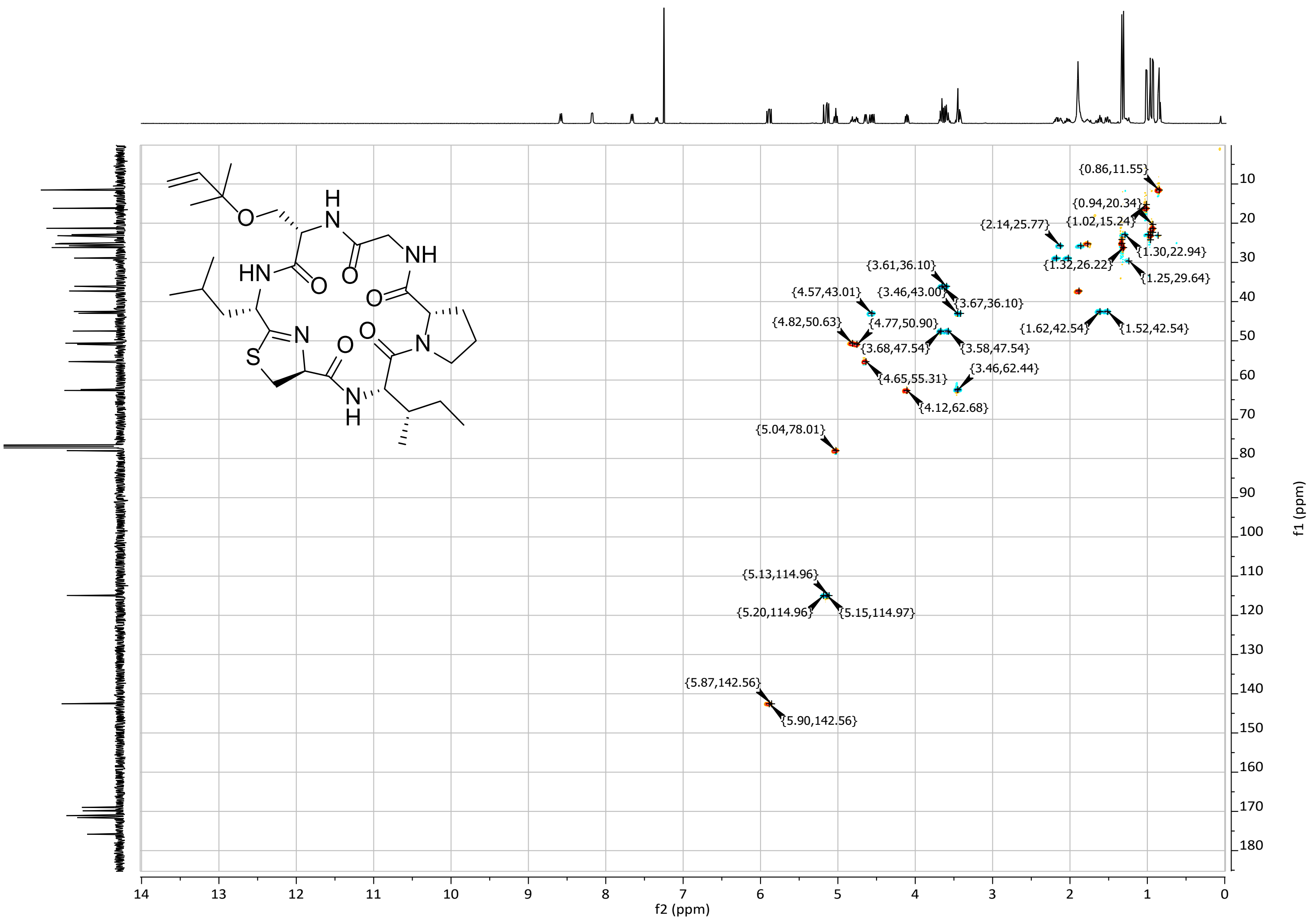

Figure S21 ^{1}H-^{13}C HSQC spectrum of **2** ($CDCl_3$, 500 MHz).

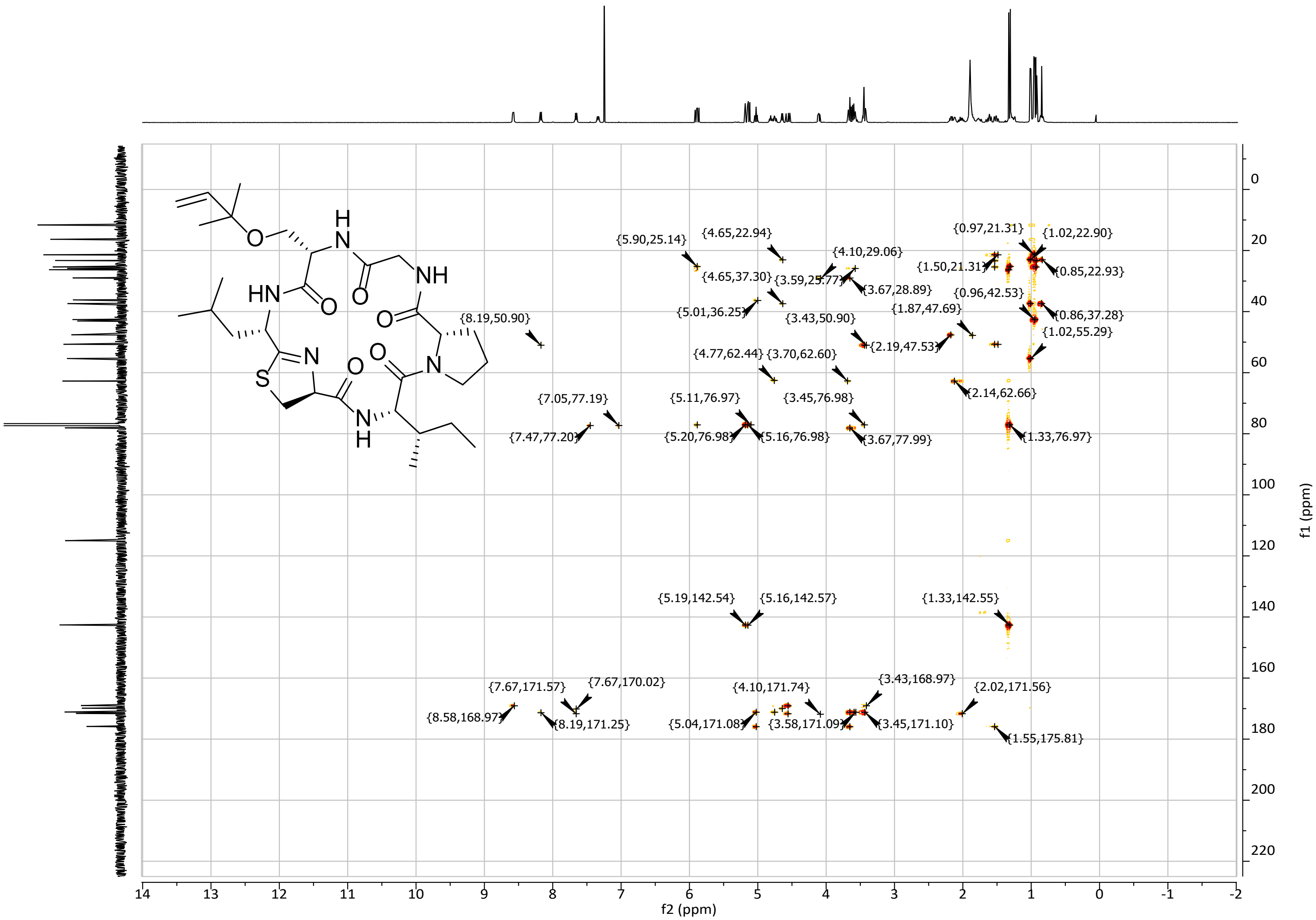

Figure S22 ^{1}H-^{13}C HMBC spectrum of **2** ($CDCl_3$, 500 MHz).

Supplementary data for cyclo[Ser(rPr)-D-Leu-Tzn-Ile-Pro-Gly] (3)

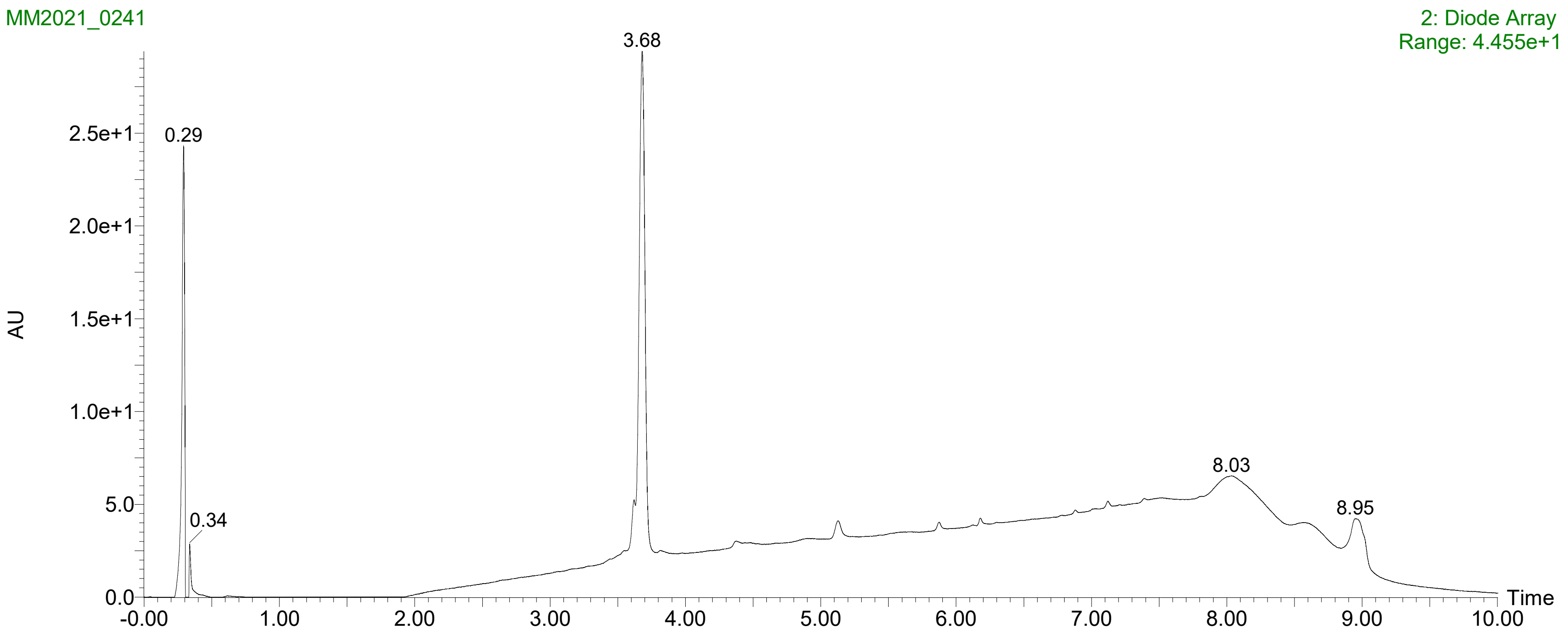

Figure S23 HPLC trace of **3**. 5-100% MeCN/H_2O + 0.1% formic acid / 10 min, Waters X Bridge C8 column (2.5 µm, 2.1 x 100 mm)

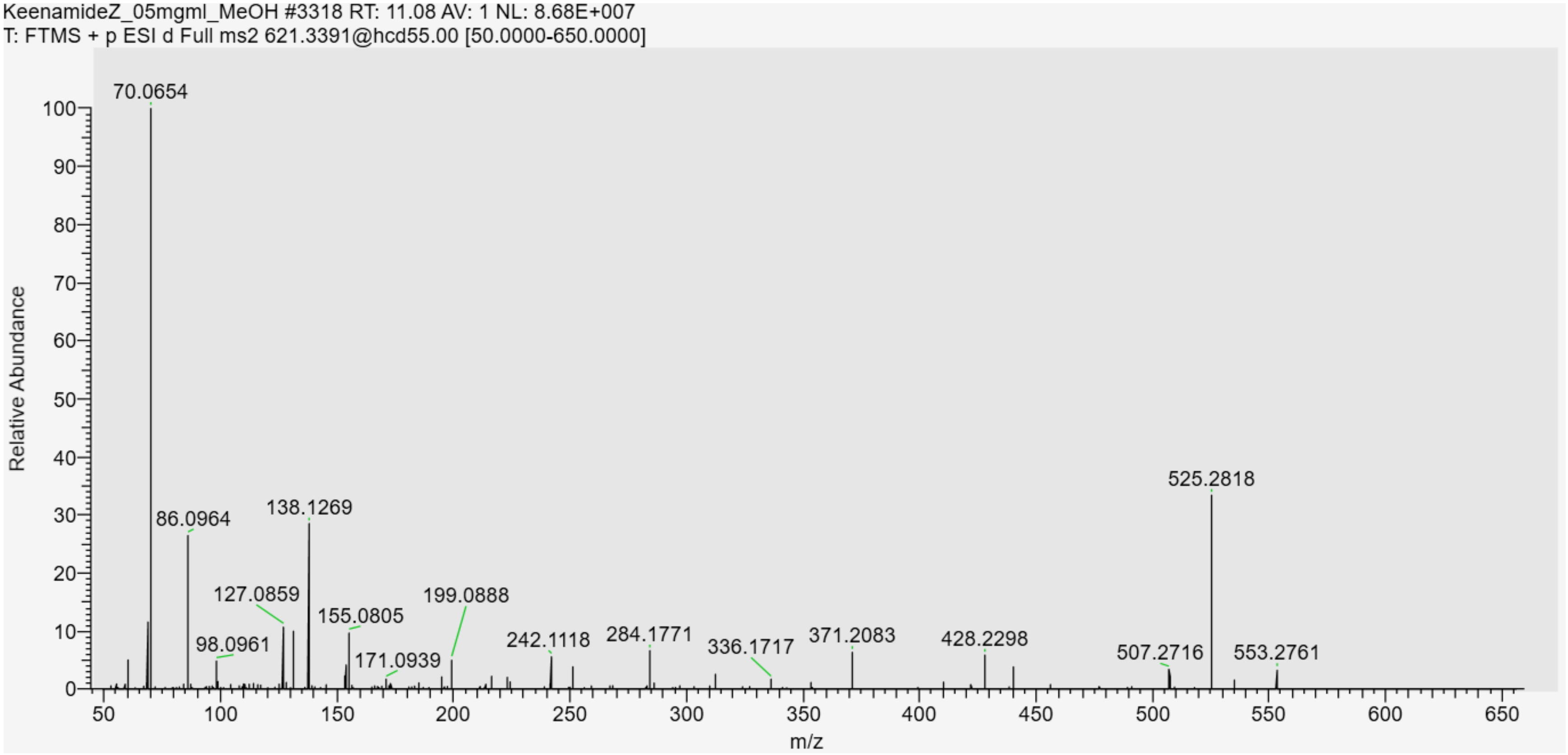

Figure S24 MS/MS spectrum of **3**. Spectrum was recorded on a Q Exactive Plus Orbitrap mass spectrometer; precursor selection window was set to 1.5 m/z; CE was set to 25, 50, 75, resolution was set to 35,000.

Table S3 $^1H^a$- and $^{13}C^b$-NMR Data ($CDCl_3$) for **3**

position		δ_C	δ_H (*J* in Hz)
Gly	1	171.6	
	2a	44.2	4.54 dd (15.8, 9.9)
	2b		3.54, m
	NH		7.08, br d (9.5)
Pro	1	170.5	
	2	63.0	3.85, dd (10.1, 6.8)
	3a	28.5	2.13, m
	3b		2.03, m
	4a	26.3	2.12, m
	4b		1.78, m
	5a	47.8	3.68, m
	5b		3.56, m
Ile	1	171.6	
	2	56.0	4.64, dd (9.0, 4.6)
	3	37.1	1.96, m
	4	16.1	0.95, d (6.4)
	5a	23.4	1.39, qd (7.4, 3.4)
	5b		1.19, m
	6	12.1	0.88, t (7.3)
	NH		7.23, d (9.0)
Tzn	1	170.8	
	2	78.6	5.06, dd (10.1, 3.8)
	3a	38.0	3.66, dd (10.1, 11.5)
	3b		3.59 (11.5, 3.8)
Leu	1	179.0	
	2	51.9	4.50, m
	3a	42.3	1.81, m
	3b		1.71, m
	4	25.0	1.89, m
	5	21.6	0.95, d (6.7)
	6	23.3	0.98, d (6.7)
	NH		7.38, d (6.0)
Ser	1	170.6	(NH) 7.25
	2	55.2	4.50
	3a	63.0	3.71, dd (8.7, 4.7)
	3b		3.32 t, (8.7)
	NH		4.50, m[c]
prenyl	1	76.2	
	2	25.8	1.27, s
	3	26.0	1.23, s
	4	143.4	5.81, dd (17.6, 10.8)
	5a	114.3	5.12, d (17.6)
	5b		5.10, d (10.8)

[a]at 700 MHz, $CHCl_3$ signal at 7.26 ppm, 293.2 K; [b]at 176 MHz, $CDCl_3$ signal at 77.16 ppm, 293.2 K; [c] signal partially covered by residual solvent peak

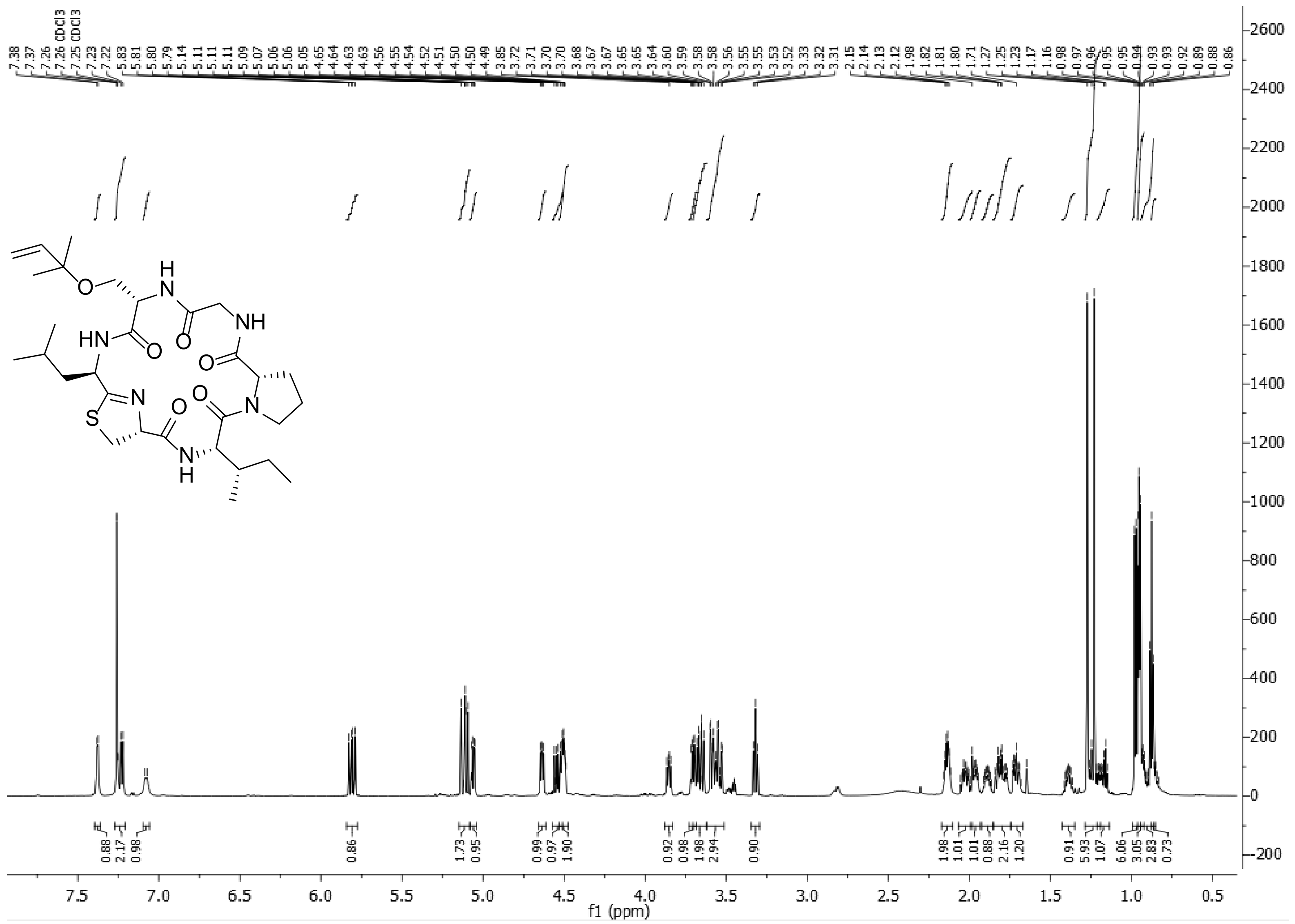

Figure S25 ^{1}H NMR spectrum of **3** ($CDCl_3$, 700 MHz).

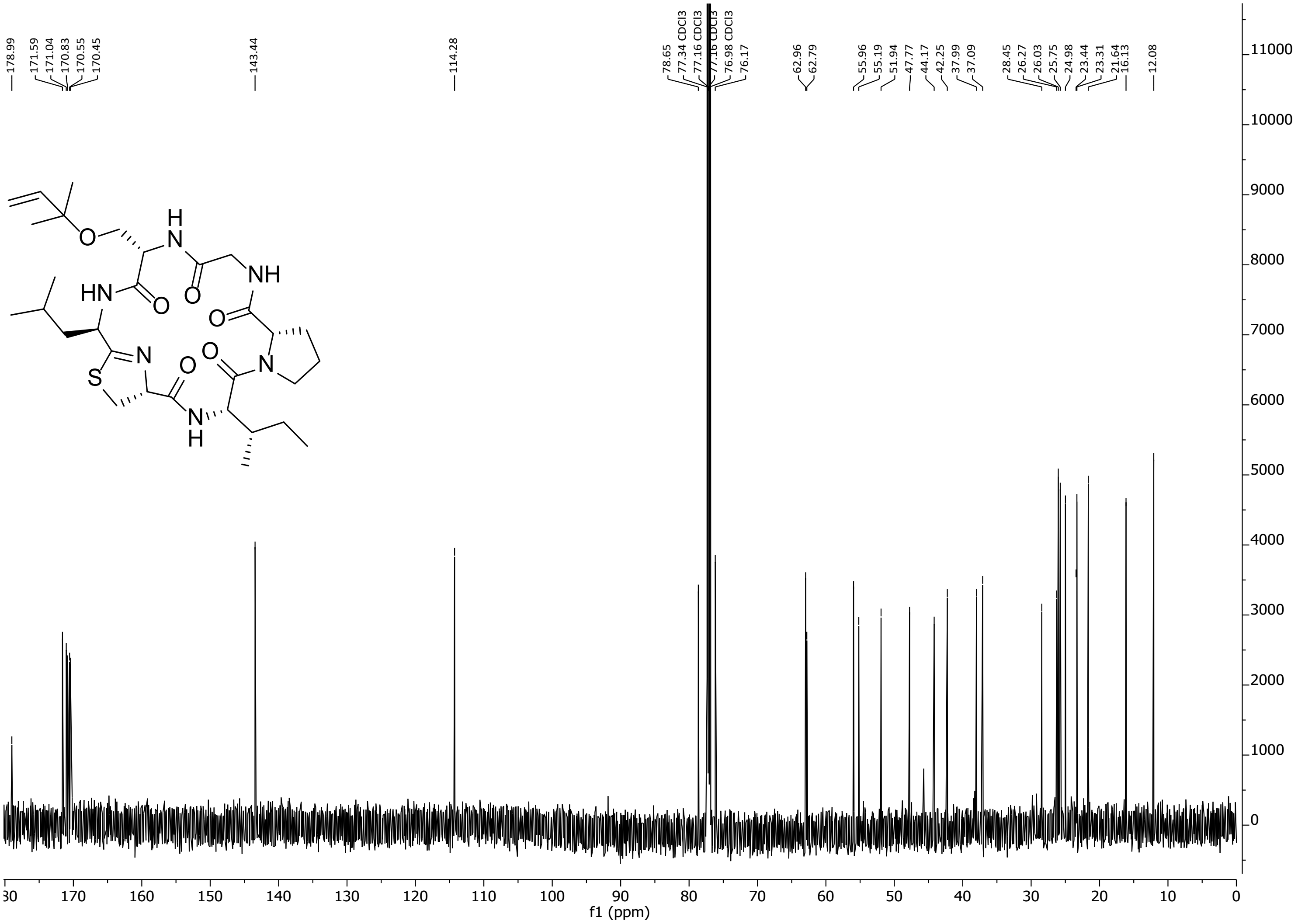

Figure S26 ^{13}C NMR spectrum of **3** ($CDCl_3$, 176 MHz).

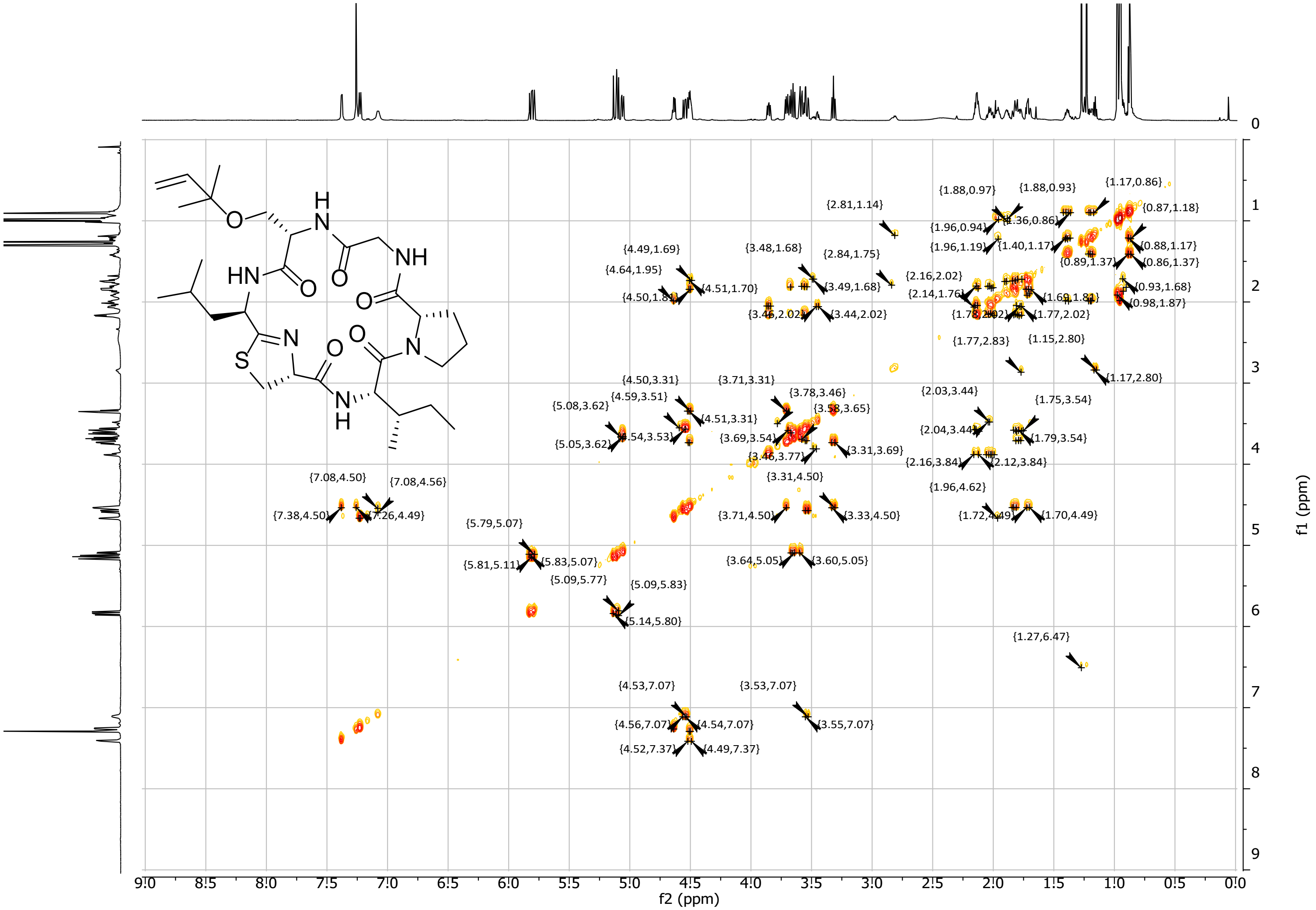

Figure S27 ^{1}H-^{1}H COSY spectrum of **3** ($CDCl_3$, 700 MHz).

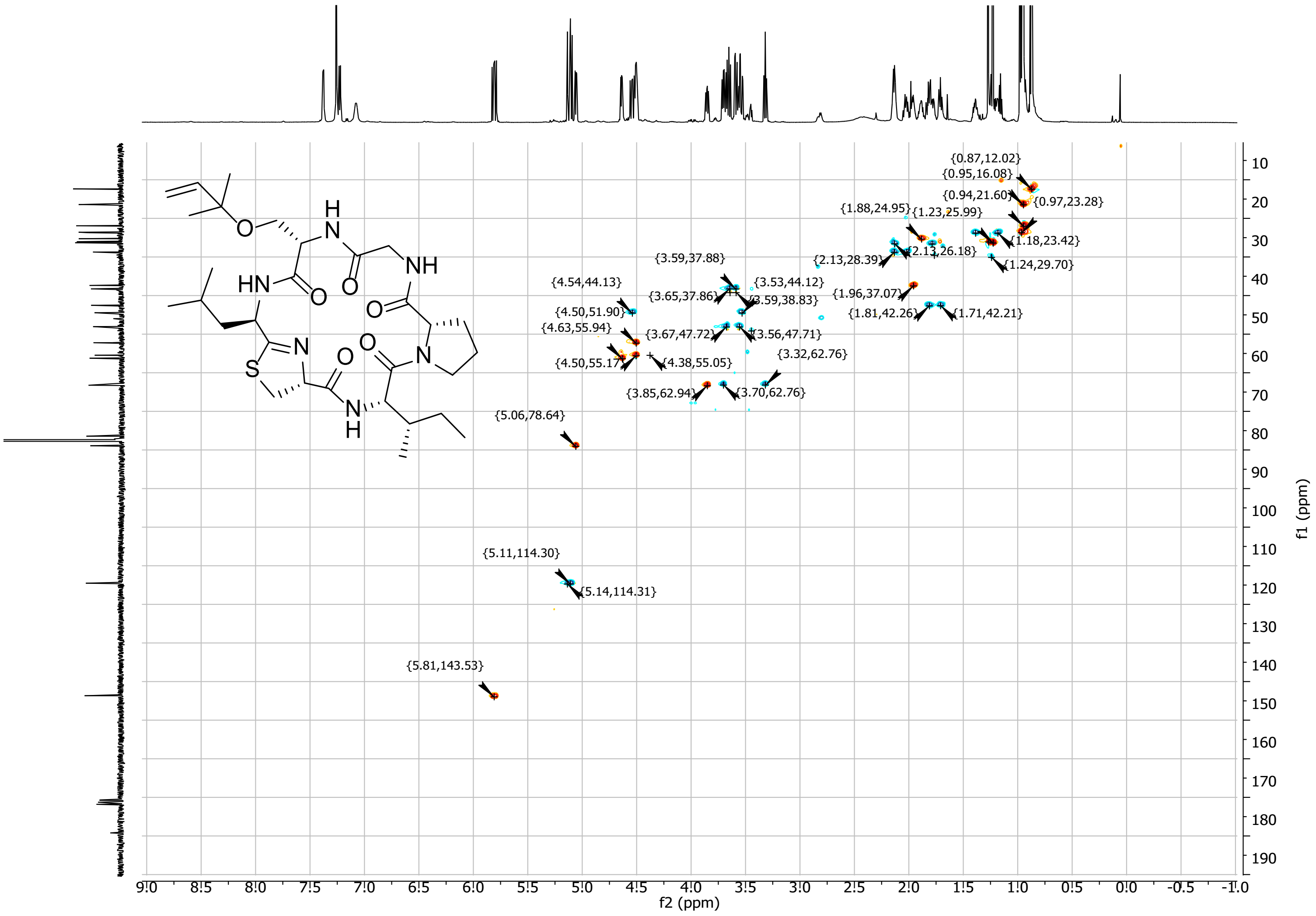

Figure S28 ^{1}H-^{13}C HSQC spectrum of **3** ($CDCl_3$, 700 MHz).

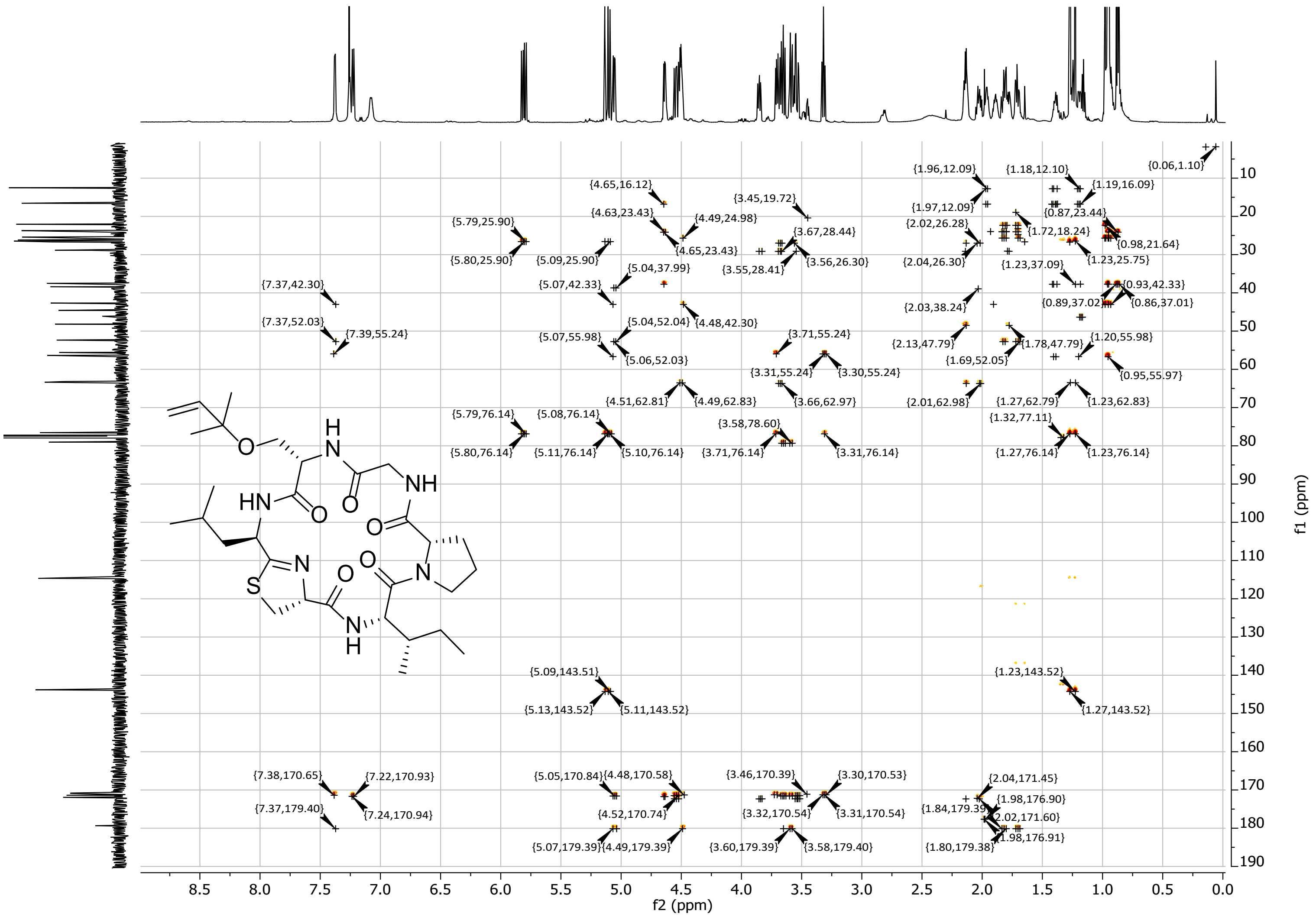

Figure S29 ^{1}H-^{13}C HMBC spectrum of **3** ($CDCl_3$, 700 MHz).

Supplementary data for cyclo[Ser(rPr)-D-Leu-D-Tzn-Ile-Pro-Gly] (4)

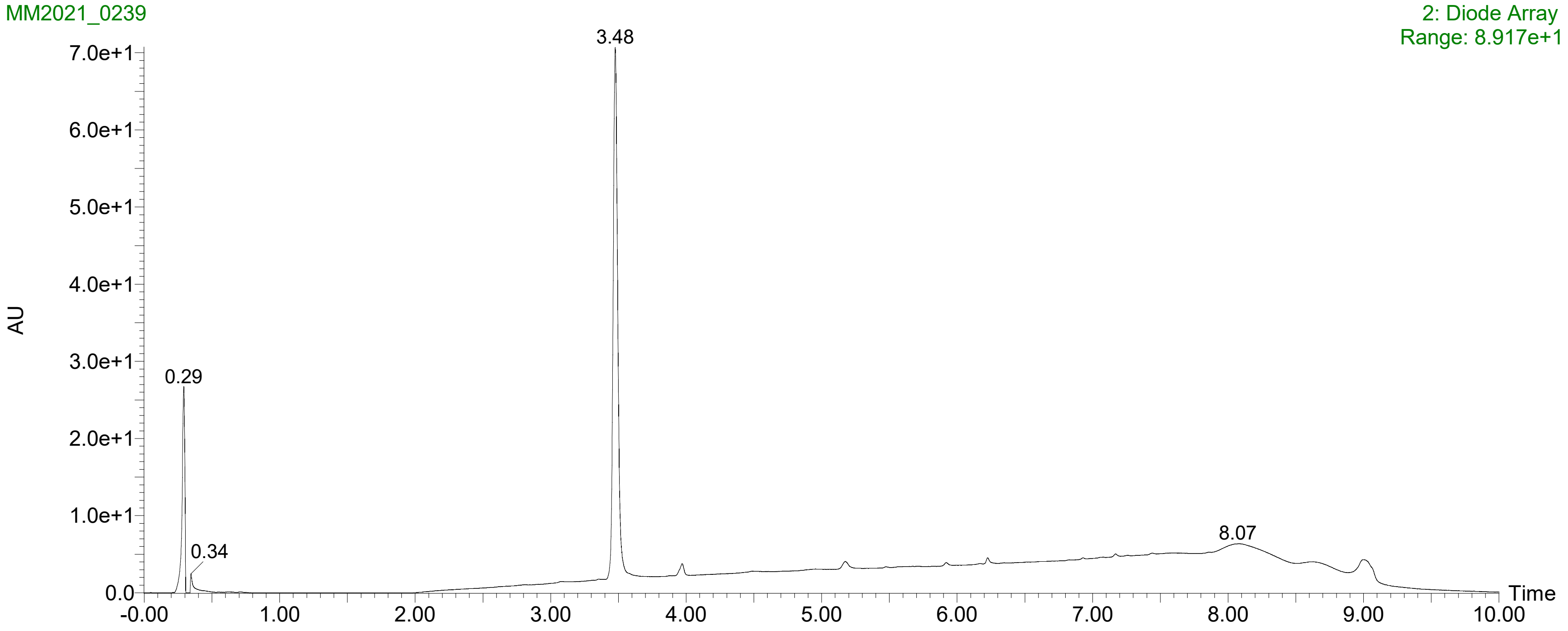

Figure S30 HPLC trace of **4**. 5-100% MeCN/H_2O + 0.1% formic acid / 10 min, Waters X Bridge C8 column (2.5 µm, 2.1 x 100 mm).

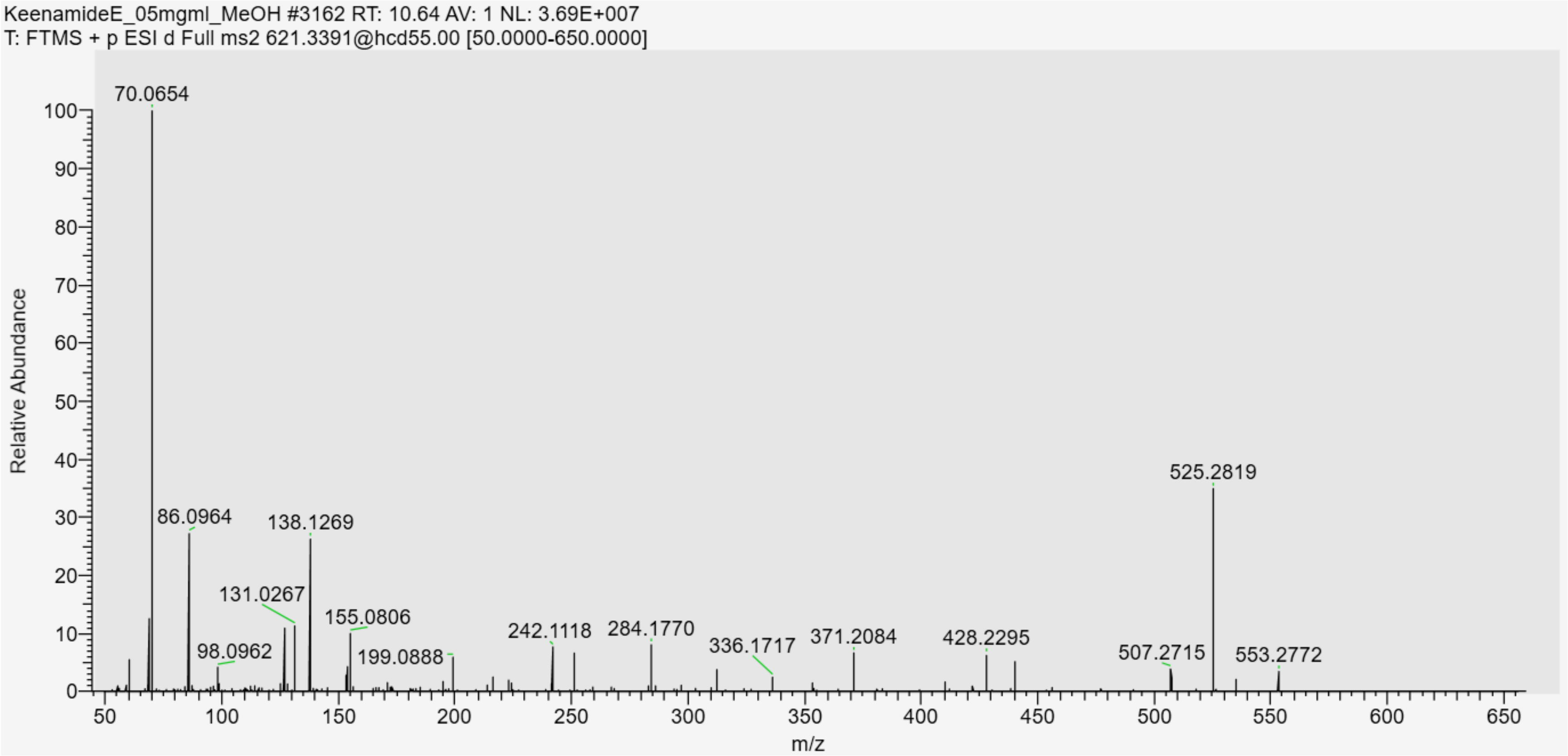

Figure S31 MS/MS spectrum of **4**. Spectrum was recorded on a Q Exactive Plus Orbitrap mass spectrometer; precursor selection window was set to 1.5 m/z; CE was set to 25, 50, 75, resolution was set to 35,000.

Table S4 $^{1}H^{a}$- and $^{13}C^{b}$-NMR Data ($CDCl_3$) for **4**

position		δ_C	δ_H (*J* in Hz)
glycine	1	169.2	
	2a	43.4	4.58, dd (10.0, 17.3)
	2b		3.46, dd (17.3, 3.2)
	NH		6.59, m
proline	1	171.7	
	2	63.1	4.06, dd (10.1, 6.6)
	3a	28.9	2.16, m
	3b		2.05, m
	4a	26.0	2.16, m
	4b		1.88, m
	5a	47.6	3.71, br t (8.7)
	5b		3.59, m
isoleucine	1	170.5	
	2	53.6	4.66, m
	3	36.9	1.94, m
	4	16.3	1.02, d (6.8)
	5a	23.2	1.33, m
	5b		1.05, m
	6	11.8	0.86
	NH		7.69, d (9.5)
thiazoline	1	171.5	
	2	78.6	5.05, td (9.7, 2.4)
	3a	36.1	3.62, br t (10.4)
	3b		3.52, dd (9.3, 11.4)
leucine	1	157.6	
	2	53.6	4.11
	3a	40.4	2.46, m
	3b		1.48, m
	4	25.2	1.69, m
	5	23.4	0.96, d (6.6)
	6	21.3	0.92, d (6.6)
	NH		7.71, d (6.8)
serine	1	170.6	8.46 (NH)
	2	52.4	4.66, m
	3a	62.8	3.50, dd (4.6, 8.7)
	3b		3.37, dd (10.2, 8.7)
	NH		8.46, d (8.1)
isoprene	1	76.9	
	2	25.3	1.31, s
	3	26.5	1.28, s
	4	143.0	5.86, dd (17.5, 10.8)
	5a	114.8	5.15, d (17.5)
	5b		5.11, d (10.8)

^{a}at 700 MHz, $CHCl_3$ signal at 7.26 ppm, 293.2 K; ^{b}at 176 MHz, $CDCl_3$ signal at 77.16 ppm, 293.2 K

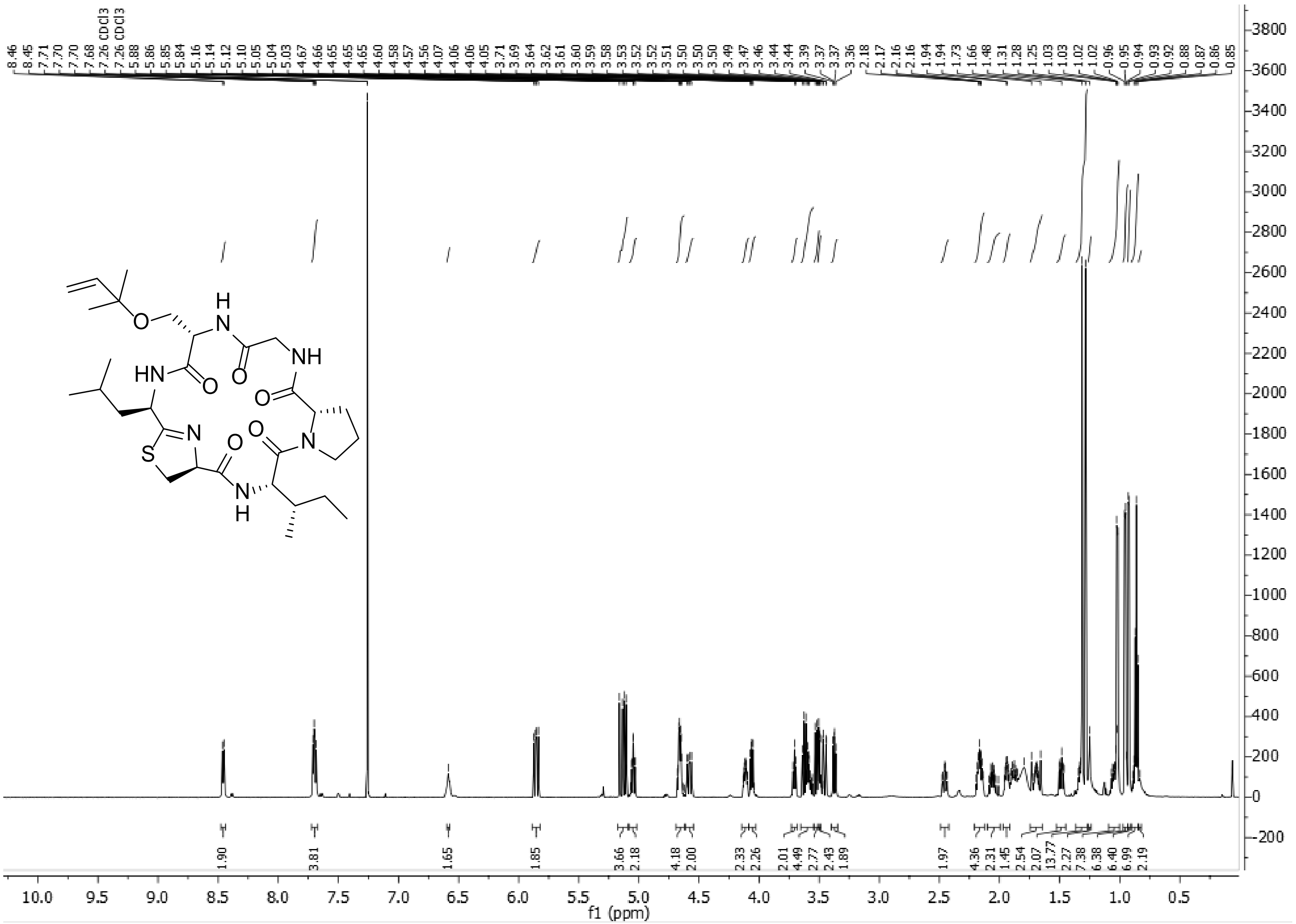

Figure S32 ^{1}H NMR spectrum of **4** ($CDCl_3$, 700 MHz).

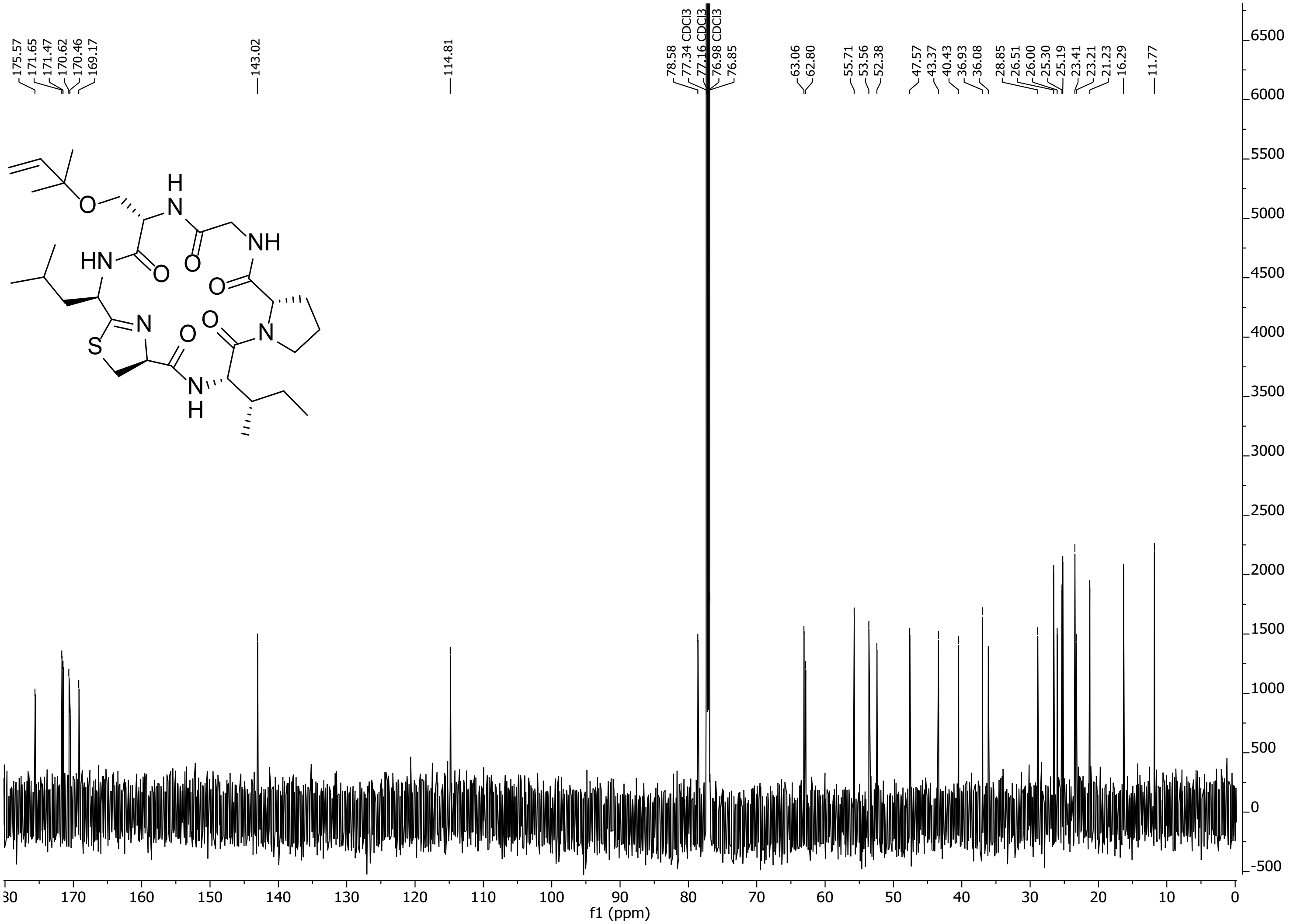

Figure S33 ^{13}C NMR spectrum of **4** ($CDCl_3$, 176 MHz).

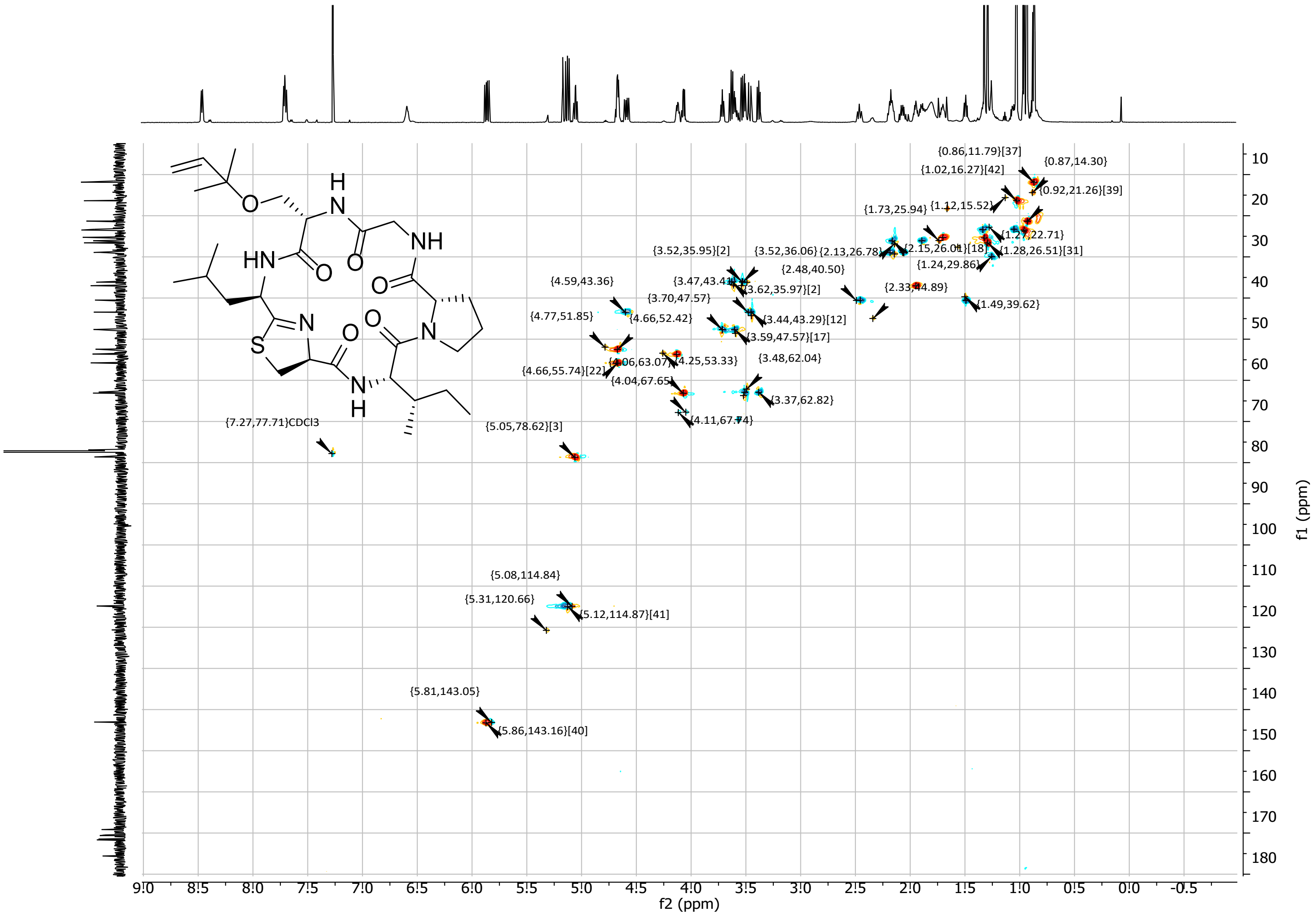

Figure S34 ^{1}H-^{13}C HSQC NMR spectrum of **4** ($CDCl_3$, 700 MHz).

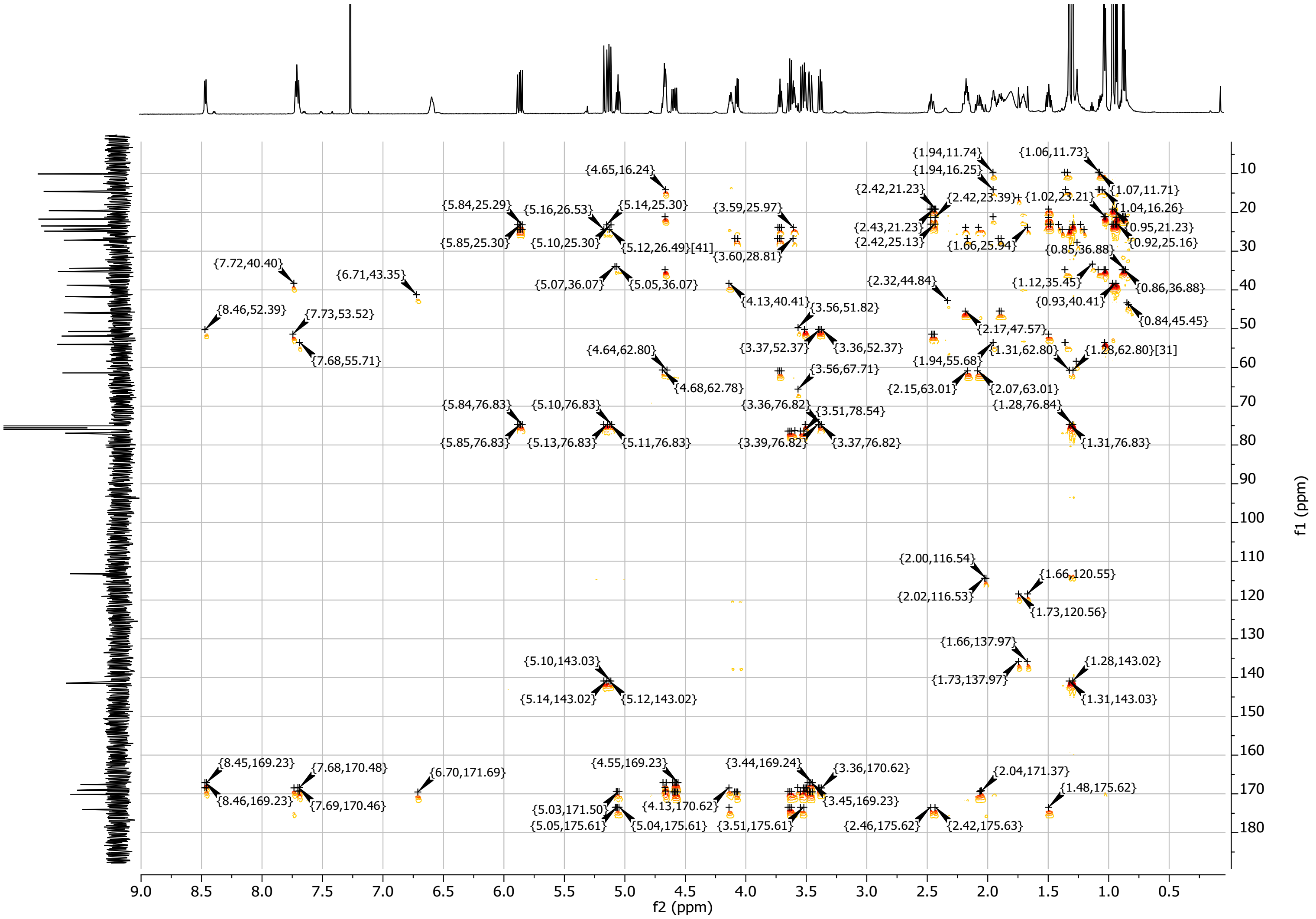

Figure S35 ^{1}H-^{13}C HMBC NMR spectrum of **4** ($CDCl_3$, 700 MHz).

Supplementary data for cyclo[Ser(rPr)-Leu-Tzl-Ile-Pro-Gly] (mollamide C)

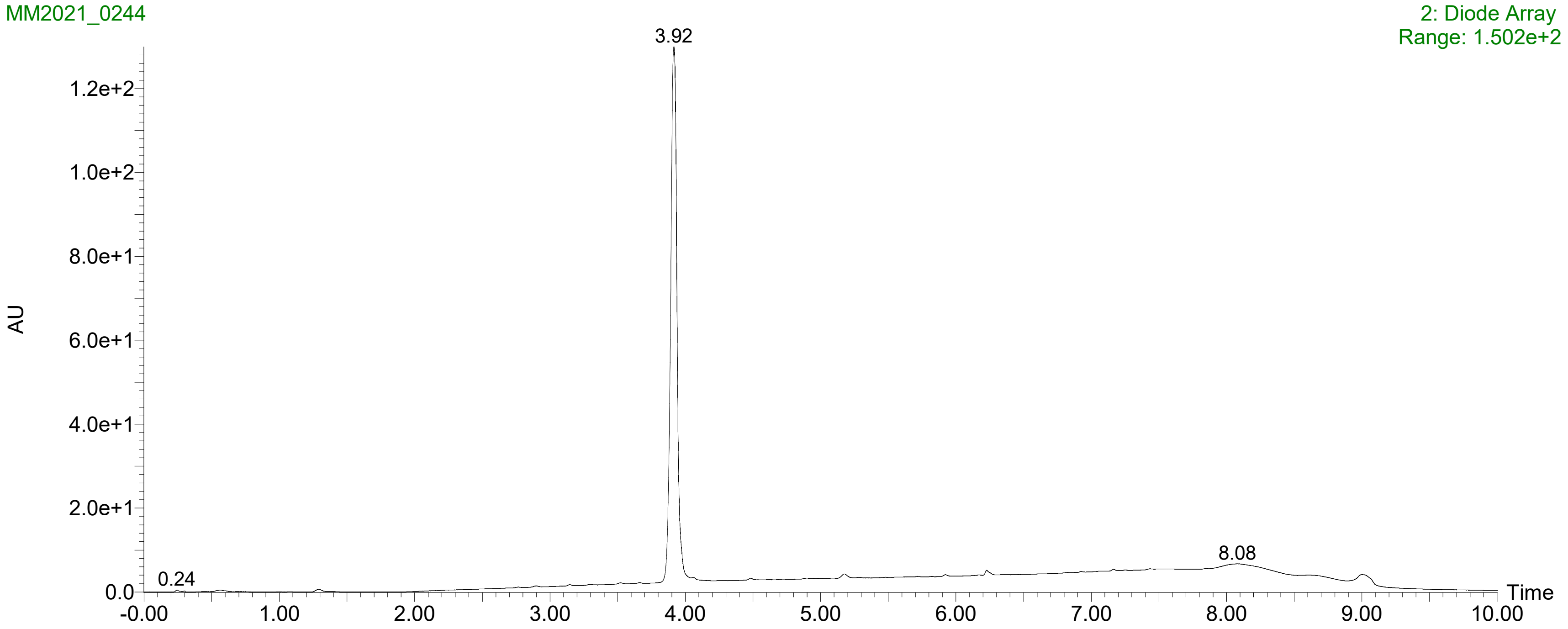

Figure S36 HPLC trace of cyclo[Ser(rPr)-Leu-Tzl-Ile-Pro-Gly] (mollamide C). 5-100% MeCN/H_2O + 0.1% formic acid / 10 min, Waters X Bridge C8 column (2.5 µm, 2.1 x 100 mm).

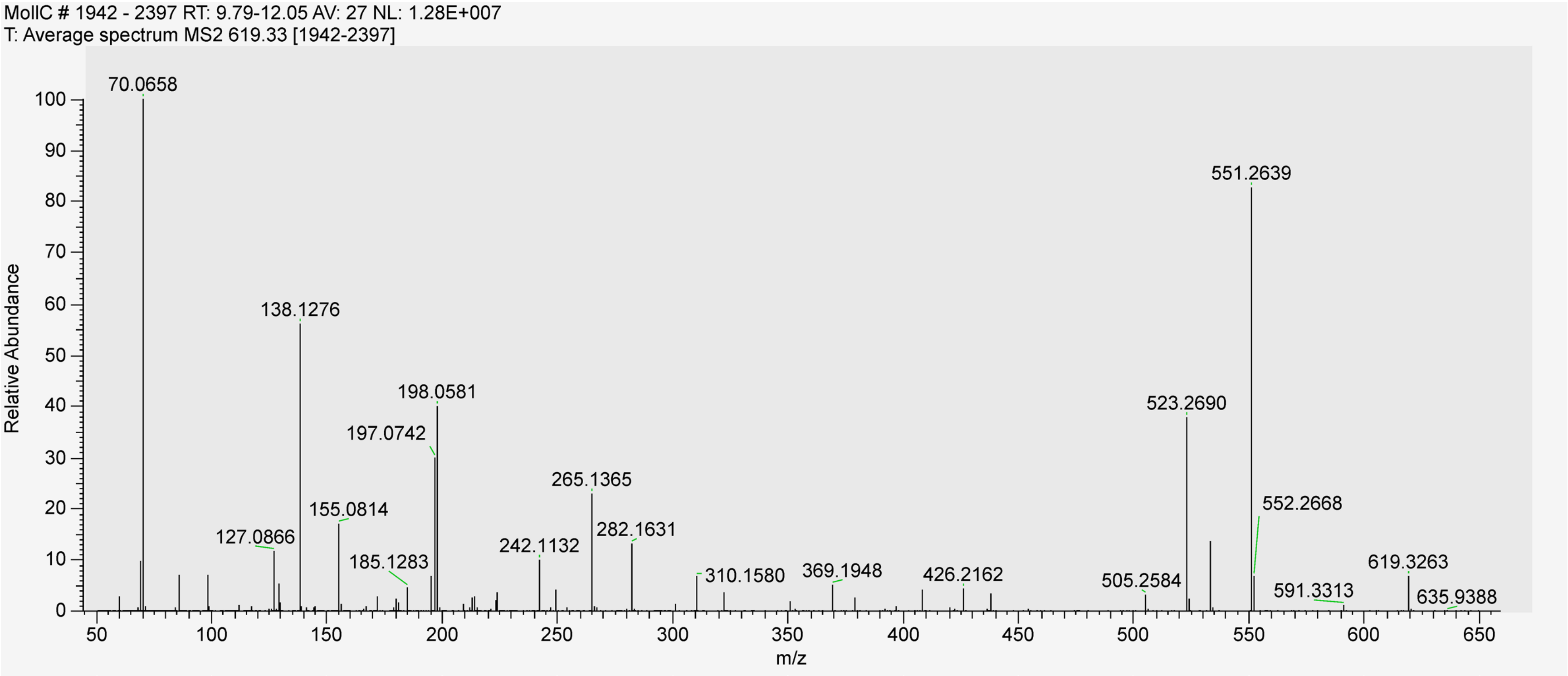

Figure S37 MS/MS spectrum of mollamide C. Spectrum was recorded on a Q Exactive Plus Orbitrap mass spectrometer; precursor selection window was set to 1.5 m/z; CE was set to 25, 50, 75, resolution was set to 35,000.

Table S5 $^{1}H^{a}$- and $^{13}C^{b}$-NMR Data ($CDCl_3$) for mollamide C

position		δ_C	δ_H (*J* in Hz)
Gly	1	169.2	
	2	43.2	4.60, dd (17.6, 10.0)
			3.43, dd (17.6, 3.1)
		NH	7.06, br d (9.9)
Pro	1	171.7	
	2	63.4	4.05, dd (10.1, 6.6)
	3	28.8	2.18, m
			2.08, m
	4	26.1	2.17, m
			1.86, m
	5	47.4	3.70, br t (8.8)
			3.62, m
Ile	1	161.7	
	2	56.9	4.58
	3	36.9	1.98
	4	16.7	1.10, d (6.7)
	5	23.1	1.33, m
			1.03, m
	6	11.8	0.8, t (7.4)
	NH		7.70, d (8.9)
Tzl	1	170.8	
	2	122.9	7.96, s
	3	149.2	
Leu	1	170.6	
	2	50.6	5.24, ddd (15.2, 8.4, 4.8)
	3	44.4	1.82, m
	4	25.5	1.93, m
	5	21.4	1.03, d (6.5)
	6	23.6	1.03, d (6.7)
	NH		8.17, d (8.5)
Ser	1	172.4	
	2	51.1	4.96, ddd (10.8, 9.3, 4.3)
	3a	62.9	3.62, m
	3b		3.46, dd (8.9, 4.4)
	NH		8.56, d (9.3)
prenyl	1	77.1	
	2	26.4	1.26, s
	3	25.3	1.30, s
	4	142.4	5.74, dd (17.5, 10.8)
	5	115.2	5.13, d (17.5)
			5.07, d (10.8)

[a]at 500 MHz, $CHCl_3$ signal at 7.26 ppm, 293.2 K; [b]at 126 MHz, $CDCl_3$ signal at 77.16 ppm, 293.2 K

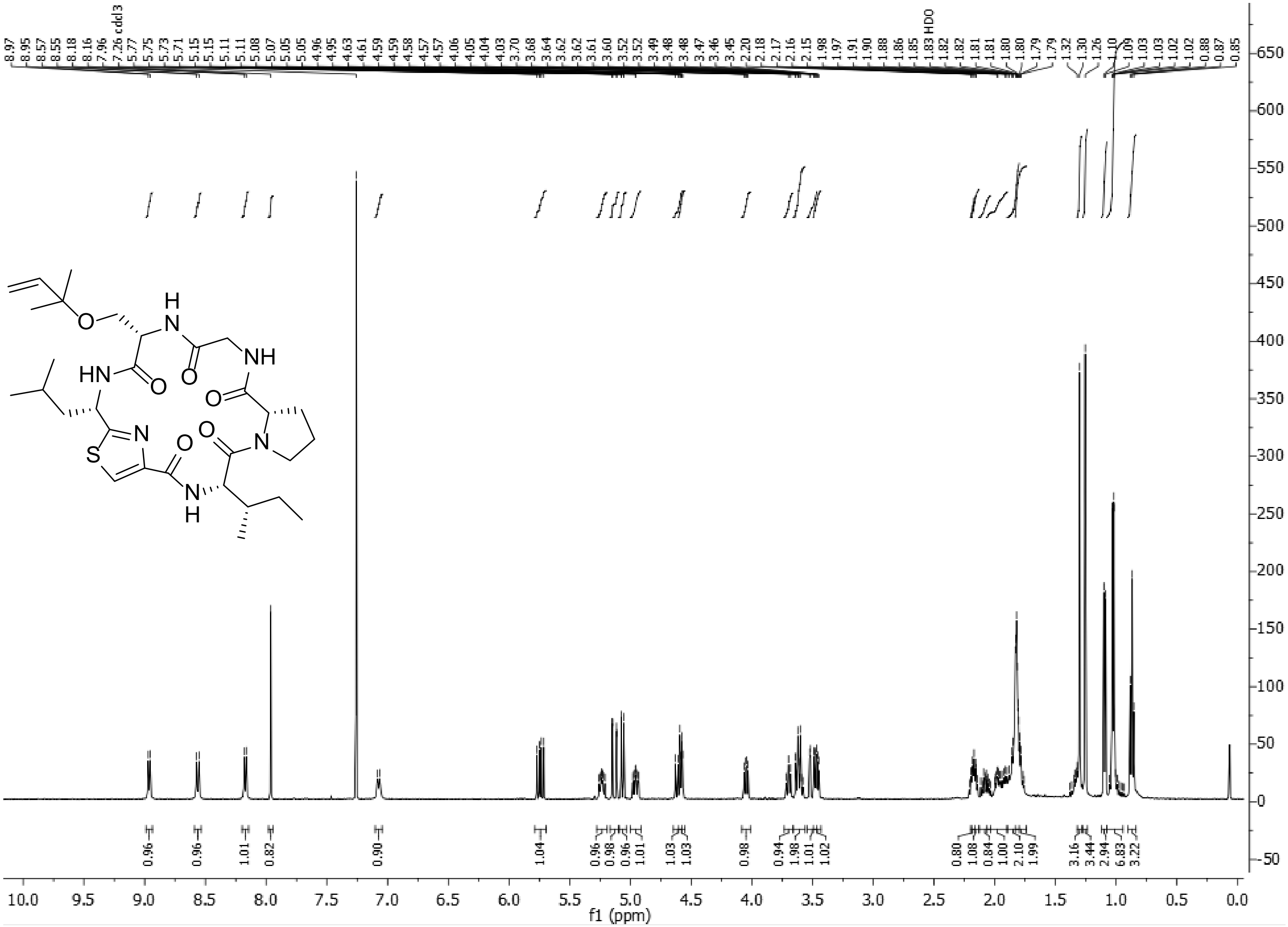

Figure S38 ^{1}H NMR spectrum of mollamide C ($CDCl_3$, 500 MHz).

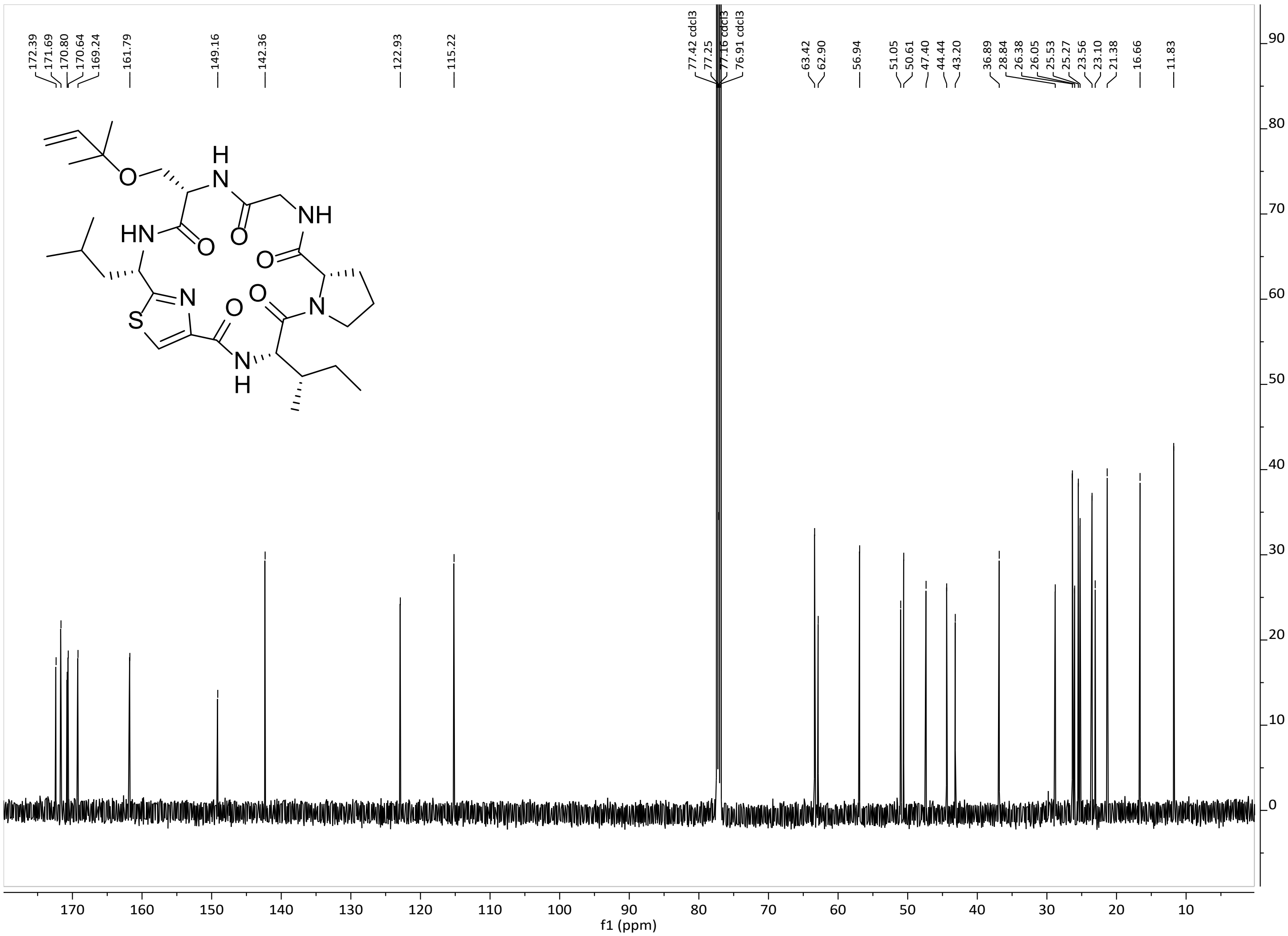

Figure S39 ^{13}C NMR spectrum of mollamide C ($CDCl_3$, 125 MHz).

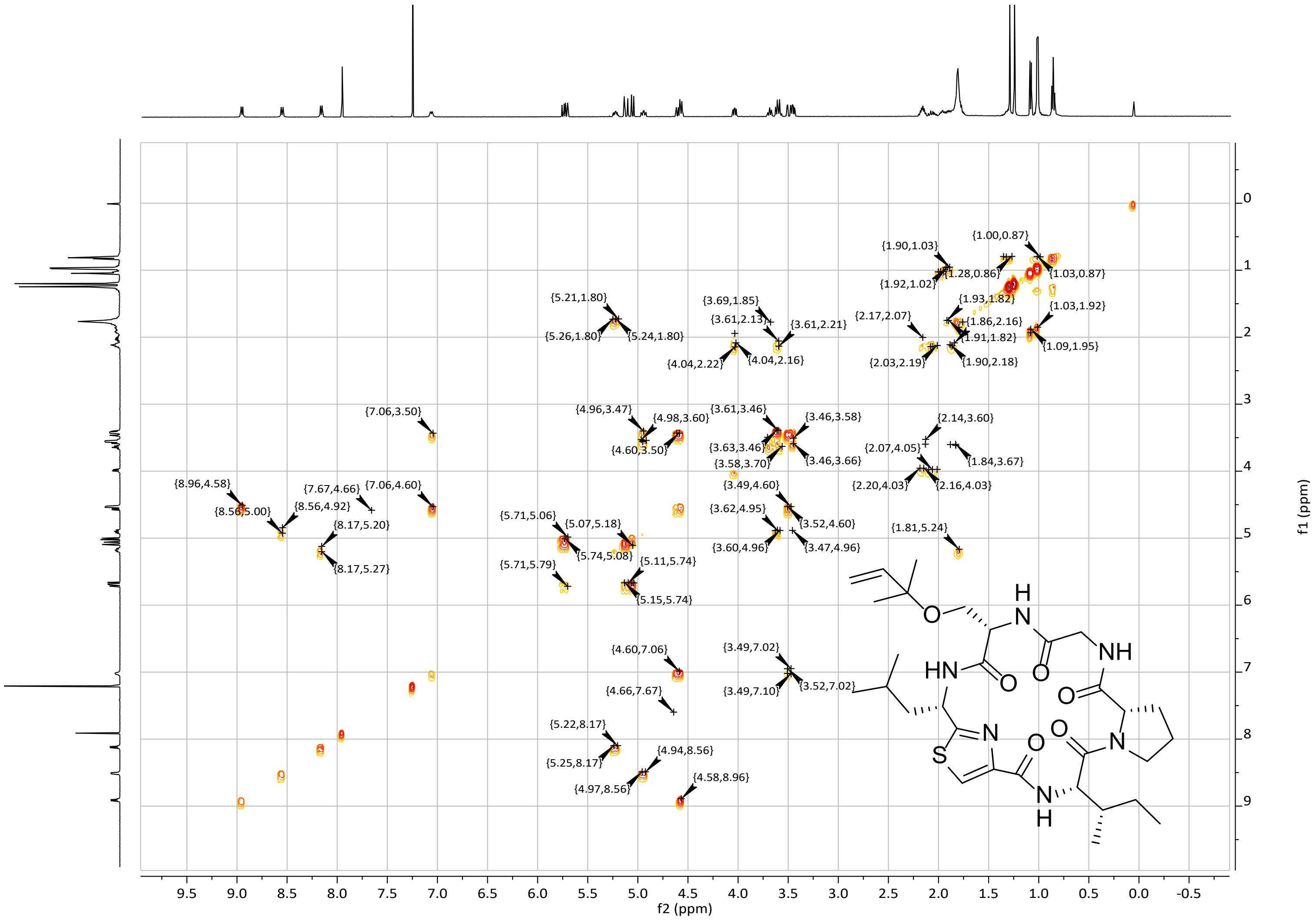

Figure S40 ^{1}H-^{1}H COSY spectrum of mollamide C ($CDCl_3$, 500 MHz).

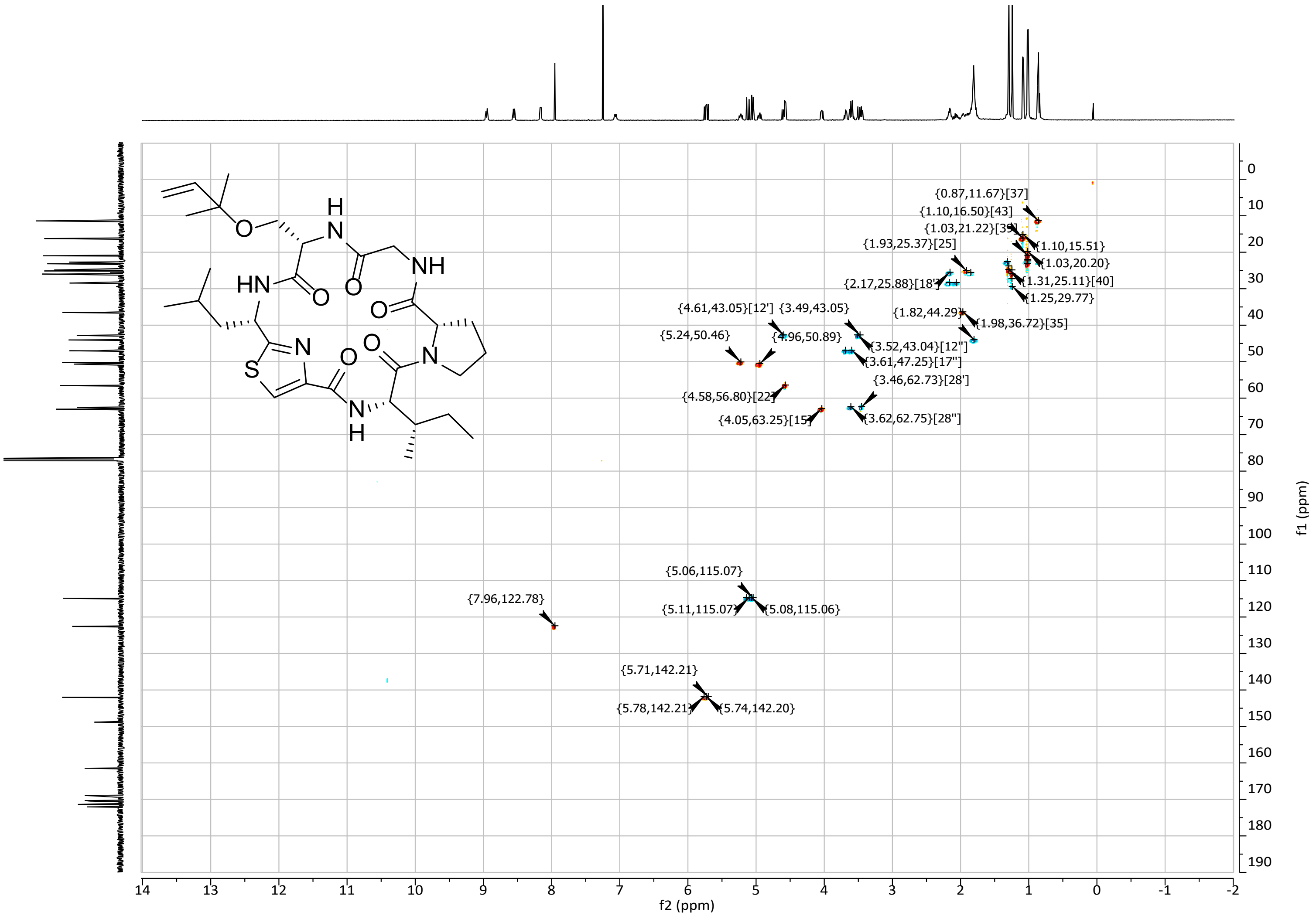

Figure S41 ^{1}H - ^{13}C HSQC NMR spectrum of mollamide C ($CDCl_3$, 500 MHz).

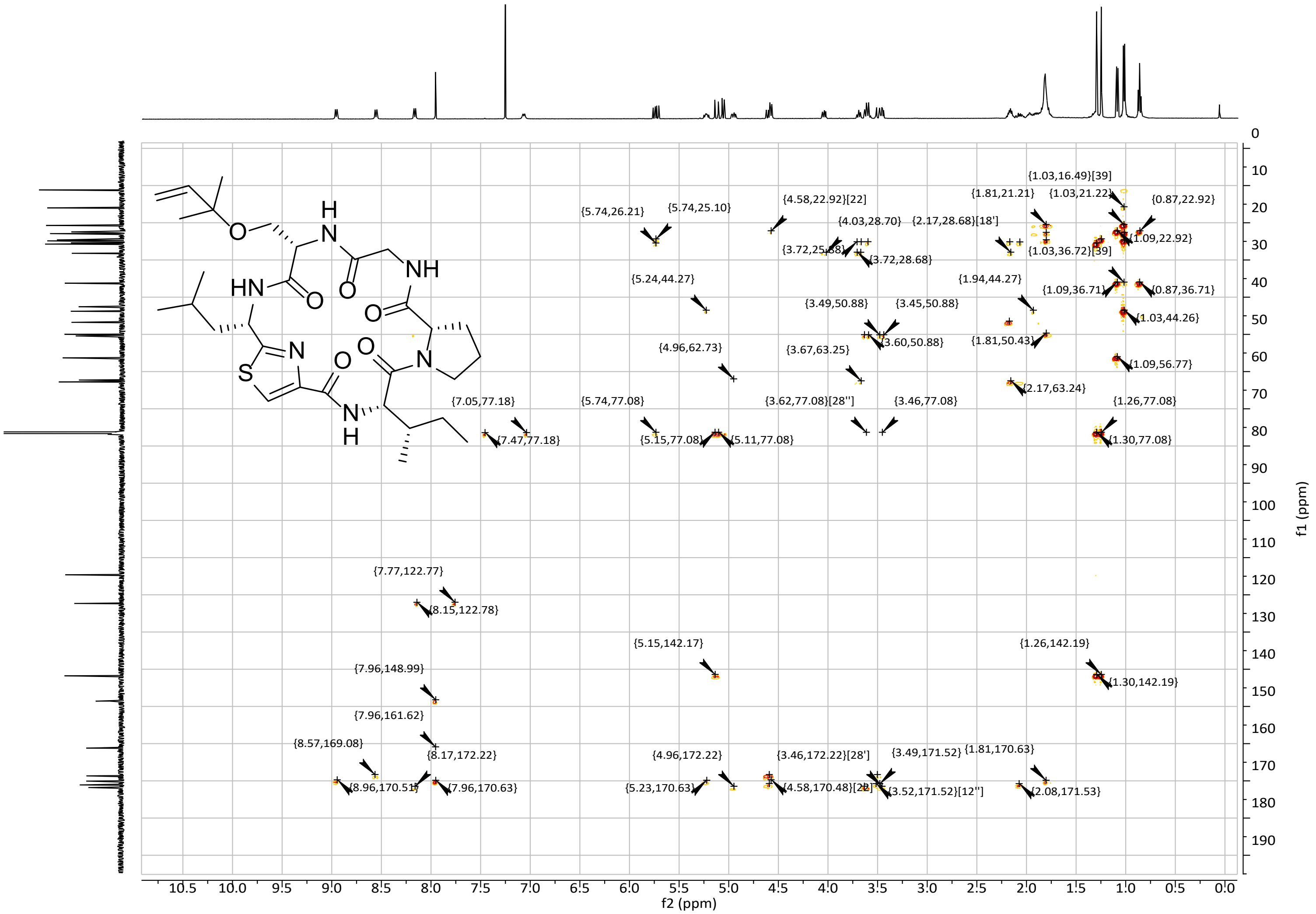

Figure S42 ^{1}H - ^{13}C HMBC NMR spectrum of mollamide C ($CDCl_3$, 500 MHz).

Comparison of chemical shifts of keenamide stereoisomers with original spectral data

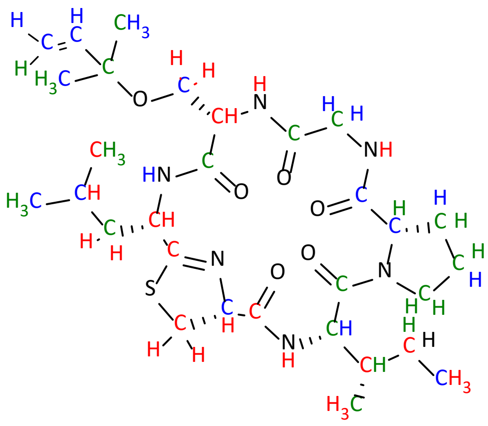

1 (L-Leu / L-Tzn)

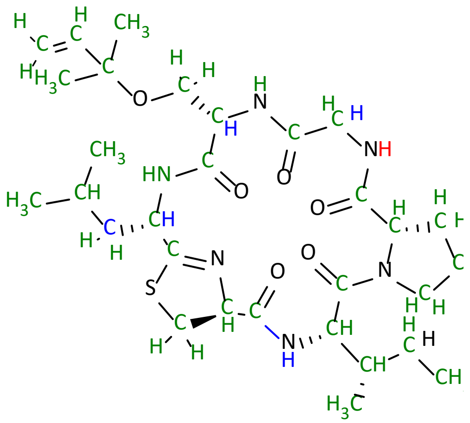

2 (L-Leu / D-Tzn)

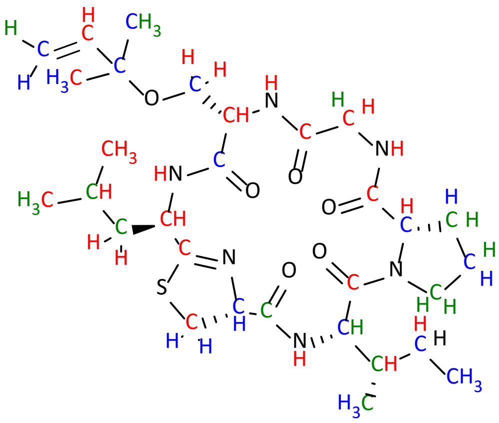

3 (D-Leu / L-Tzn)

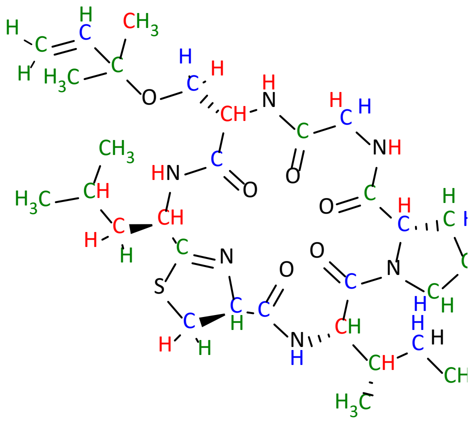

4 (D-Leu / D-Tzn)

Figure S43 Structures of keenamide A stereoisomers coloured by NMR chemical shift difference to natural keenamide A. Hydrogen and carbon atoms are coloured according to their chemical shift difference Δ to the values reported by Wesson and Hamann.[1] (hydrogen: green: $\Delta \leq 0.03$ ppm, blue: $0.03 \text{ ppm} < \Delta < 0.06$ ppm, red: $\Delta \geq 0.06$ ppm; carbon: green: $\Delta \leq 0.3$ ppm, blue: $0.3 \text{ ppm} < \Delta < 0.9$ ppm, red: $\Delta \geq 0.9$ ppm). For one isoleucine H_γ (black) no chemical shift was reported by Wesson and Hamann (1996).[1]

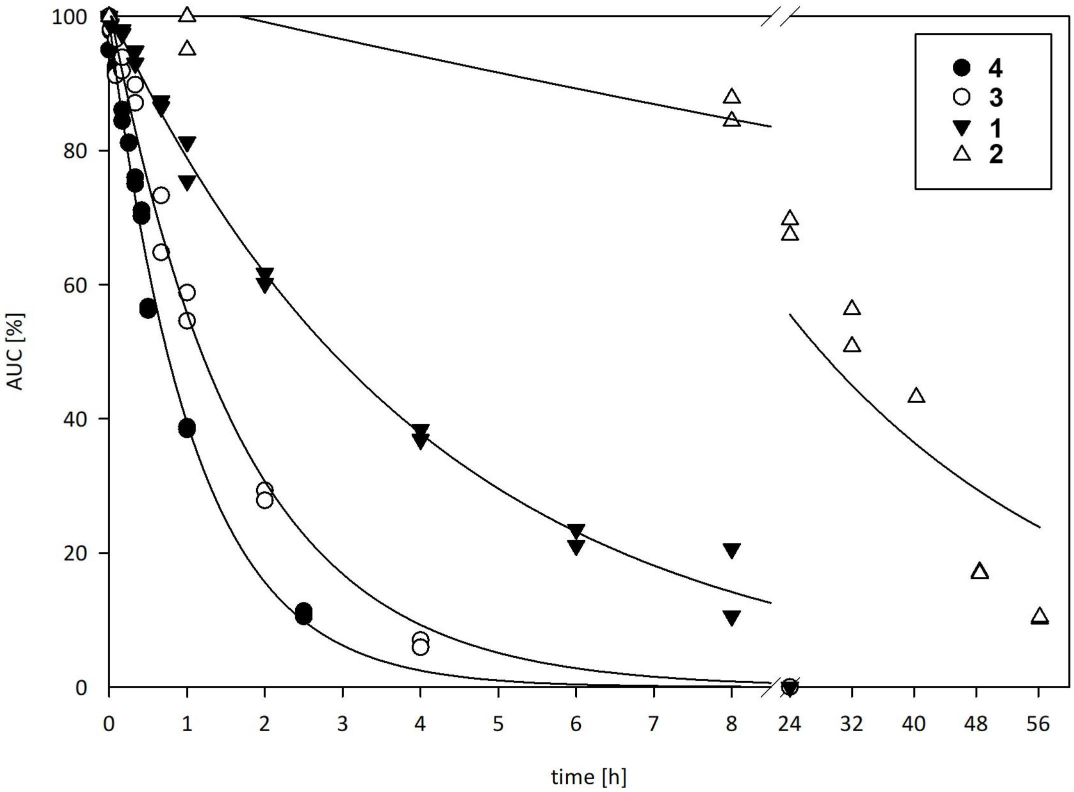

Figure S44 Stability of keenamide A stereoisomers. **1**-**4** were dissolved in a 50 mM HCl/KCl buffer (pH 2.0, 50% MeCN/H_2O, 1% DMF) at room temperature. At indicated time points, aliquots (10 µl) were diluted in triethylammonium acetate buffer (50 µl, 500 mM, pH 7.0 50% MeCN/H_2O) and analyzed with analytical HPLC. Percentages of residual starting material were calculated based on area under curve (AUC) at a detection wavelength of 220 nm and plotted against reaction time. Half-life times were calculated using a first-order exponential decay model: **1**, $t_{1/2}$ = 169 min; **2**, $t_{1/2}$ = 28 h; **3**, $t_{1/2}$ = 69 min; **4**, $t_{1/2}$ = 44 min.

Supplementary crystallographic data

Table S6 Crystal data and refinement details for keenamide A · 1.5 H_2O and mollamide C · H_2O.

	**keenamide A** · 1.5 H_2O	**mollamide C** · H_2O
Empirical formula	$C_{30}H_{48}N_6O_{7.50}S$	$C_{30}H_{48}N_6O_7S$
M_r	644.80	636.80
T (K)	100(2)	101(2)
λ (Å)	1.54184	1.54184
Crystal system, space group	monoclinic, $P2_1$	orthorhombic, $P2_12_12_1$
a (Å)	10.41956(19)	10.7789(2)
b (Å)	10.15633(15)	12.3287(3)
c (Å)	17.1392(3)	25.5913(6)
β (°)	107.225(2)	90
V (Å^3)	1732.40(5)	3400.82(13)
Z, ρ (g cm^{-3})	2, 1.236	4, 1.244
μ (mm^{-1})	1.272	1.278
F(000)	692	1368
Crystal size (mm)	0.560 × 0.070 × 0.040	0.036 × 0.027 × 0.015
θ range for data collection (°)	2.699 to 75.262	3.454 to 76.740
Reflections collected / unique	17762 / 5641	35193 / 7079
R_{int}	0.0216	0.0908
Completeness to θ = 67.684° (%)	99.6	100.0
Data / restraints / parameters	5641 / 273 / 462	7079 / 169 / 443
Goodness-of-fit on F^2	1.054	1.105
$R1$ [$I > 2\sigma(I)$]	0.0603	0.0658
$wR2$ (all data)	0.1803	0.1827
Flack x parameter	0.018(9)	–0.009(16)
$\Delta\rho_{max}$, $\Delta\rho_{min}$ (eÅ^{-3})	0.621 and -0.294	0.320, -0.367

References

(1) Wesson, K. J.; Hamann, M. T. Keenamide A, a Bioactive Cyclic Peptide from the Marine Mollusk Pleurobranchus forskalii. *J. Nat. Prod.* **1996**, *59* (6), 629–631. DOI: 10.1021/np960153t.
